# A mixed methods systematic review on the effects of arts interventions for children and young people at‐risk of offending, or who have offended on behavioural, psychosocial, cognitive and offending outcomes: A systematic review

**DOI:** 10.1002/cl2.1377

**Published:** 2024-01-03

**Authors:** Louise Mansfield, Norma Daykin, Neil E. O'Connell, Daniel Bailey, Louise Forde, Robyn Smith, Jake Gifford, Garcia Ashdown‐Franks

**Affiliations:** ^1^ Life Sciences Brunel University London Uxbridge UK; ^2^ Health and Social Wellbeing UWE Bristol UK; ^3^ Department of Health Sciences, Centre for Health and Wellbeing Across the Lifecourse Brunel University London Uxbridge UK; ^4^ Life Sciences Brunel University London London UK; ^5^ Brunel Law School Brunel University London Uxbridge UK; ^6^ Department of Life Sciences Brunel University London Uxbridge UK

## Abstract

**Background:**

Young people who enter the justice system experience complex health and social needs, and offending behaviour is increasingly recognised as a public health problem. Arts interventions can be used with the aim of preventing or reducing offending or reoffending.

**Objectives:**

1. To evaluate evidence on the effectiveness and impact of arts interventions on keeping children and young people safe from involvement in violence and crime. 2. To explore factors impacting the implementation of arts interventions, and barriers and facilitators to participation and achievement of intended outcomes. 3. To develop a logic model of the processes by which arts interventions might work in preventing offending behaviours.

**Search Methods:**

We searched AMED, Academic Search Complete; APA PsycInfo; CINAHL Plus; ERIC; SocIndex; SportDiscus, Medline, CENTRAL, Web of Science, Scopus, PTSDPubs and Performing Arts Periodicals Database, Sage, the US National Criminal Justice Reference Service, the Global Policing and British Library EThOS databases, and the National Police Library from inception to January 2023 without language restrictions.

**Selection Criteria:**

We included randomised and non‐randomised controlled trials and quasi‐experimental study designs. We included qualitative studies conducted alongside intervention trials investigating experiences and perceptions of participants, and offering insight into the barriers and facilitators to delivering and receiving arts interventions. We included qualitative and mixed methods studies focused on delivery of arts interventions. We included studies from any global setting. We included studies with CYP (8–25 years) who were identified as at‐risk of offending behaviour (secondary populations) or already in the criminal justice system (tertiary populations). We included studies of interventions involving arts participation as an intervention on its own or alongside other interventions. Primary outcomes were: (i) offending behaviour and (ii) anti‐pro‐social behaviours. Secondary outcomes were: participation/attendance at arts interventions, educational attainment, school attendance and engagement and exclusions, workplace engagement, wellbeing, costs and associated economic outcomes and adverse events.

**Data Collection and Analysis:**

We included 43 studies (3 quantitative, 38 qualitative and 2 mixed methods). We used standard methodological procedures expected by The Campbell Collaboration. We used GRADE and GRADE CERQual to assess the certainty of and confidence in the evidence for quantitative and qualitative data respectively.

**Main Results:**

We found insufficient evidence from quantitative studies to support or refute the effectiveness of arts interventions for CYP at‐risk of or who have offended for any outcome. Qualitative evidence suggested that arts interventions may lead to positive emotions, the development of a sense of self, successful engagement in creative practices, and development of positive personal relationships. Arts interventions may need accessible and flexible delivery and are likely to be engaging if they have support from staff, family and community members, are delivered by professional artists, involve culturally relevant activity, a youth focus, regularity and a sustainable strategy. We found limited evidence that a lack of advocacy, low funding, insufficient wider support from key personnel in adjacent services could act as barriers to success. Methodological limitations resulted in a judgement of very low confidence in these findings.

**Authors' Conclusions:**

We found insufficient evidence from quantitative studies to support or refute the effectiveness of arts interventions for CYP at‐risk of offending or who have offended for any outcome. We report very low confidence about the evidence for understanding the processes influencing the successful design and delivery of arts interventions in this population of CYP and their impact on behavioural, psychosocial, cognitive and offending outcomes.

## 1 PLAIN LANGUAGE SUMMARY

### 1.1 There is limited evidence for the effects and impacts of arts interventions for at‐risk and offending children and young people (CYP)

There is no clear evidence for the effectiveness of arts interventions for CYP who are at‐risk of or who have offended, for behavioural (actions), psychosocial (emotional and cultural), cognitive (logic/thought) and offending outcomes.

There is limited evidence for individual positive personal experiences from arts interventions for CYP in this population. There is limited evidence that successful arts interventions need to be accessible and flexible in their timing and access to facilities, supported by staff, family and communities, with culturally relevant activity, youth‐focused, and sustainable. Lack of support, limited funding, and ineffectual support outside the justice system could be barriers to successful arts interventions for CYP.

### 1.2 What is this review about?

This review is about the effect and impact of arts interventions in preventing youth violence in CYP at‐risk of offending or who have offended.

**What is the aim of this review?**

This review examines the effects of arts interventions on behavioural (actions), psychosocial (emotional and cultural), cognitive (logic/thought) and offending behaviours in CYP at‐risk of offending or who have offended.

### 1.3 What studies are included?

We included 43 studies (three quantitative, two mixed‐methods and 38 qualitative). The studies spanned the period 2002–2022 and were mostly carried out in the UK and USA.

### 1.4 What are the main findings of this review?

We found insufficient evidence (few studies; poor quality) to support or refute the effectiveness of arts interventions for CYP at‐risk of offending or who have offended for any outcome.

Limited evidence suggests that arts interventions of different types may need to include accessible delivery sites, support from staff, family and community members, expert delivery by professional artists to whom participants could relate, culturally relevant creative activity, a youth focus, consistency, regularity and a sustainable strategy.

### 1.5 How has this intervention worked?

Arts interventions may lead to positive emotions, the development of a good sense of self, successful engagement in creative practices, and development of positive personal relationships with peers, family, prison staff and communities for CYP who are at‐risk of offending or who have offended.

### 1.6 What do the findings of this review mean?

There is insufficient evidence (few studies; poor quality) to support or refute the effectiveness of arts interventions for CYP at‐risk of offending or who have offended, and no evidence for our primary outcome ‘offending behaviour’.

The qualitative data illustrate some consensus about best practice even in the absence of outcomes evidence and the overall poor methodological quality and limited detail in the analysis within studies.

### 1.7 How up‐to‐date is this review?

The review authors searched for studies up to 2023.

## 2 BACKGROUND

### 2.1 The problem, condition or issue

Young people who enter the justice system experience complex health and social needs, and offending behaviour is increasingly recognised as a public health problem (WHO, 2015). Violent behaviours amongst young people ranging from bullying and fighting, through more severe sexual and physical assault, and to homicide are a growing concern in many countries. The pathways to offending and violence are complex and varied but often stem from poverty and disadvantage. Risk factors include material deprivation, poor educational experiences and low attainment, poor parental supervision and unstable family contexts (Daykin, 2017). Youth violence can lead to a range of problems including mental health issues and escalating risk, resulting in extensive health, social and criminal justice costs. This suggests that effective prevention programmes focused on young people are needed to address a broad range of health, education and social outcomes, and that these could deliver substantial economic savings.

In the UK there is growing concern about the increase in more serious offences involving violence committed by CYP and about growing disparities (particularly racial) in the justice system. Youth Justice Board (YJB) data for England and Wales shows that in the year April 2019 to March 2020, 19,000 children aged 10 years and upwards were cautioned or sentenced in England and Wales. Children from Black and Minority Ethnic backgrounds accounted for 32% of arrests. There has been a reduction in some forms of offending such as theft and motoring offences. However, offences relating to possession of weapons, drugs and violence have all increased, with offences involving possession of a weapon now making up 19% of all offences committed by CYP who are first‐time entrants to the justice system (YJB/Ministry of Justice, 2021). Arts interventions have been used to divert young people from offending or other undesirable behaviours. Participatory arts programmes in community and youth justice settings can offer supportive and safe interventions that can appeal to young participants (Frater, 2019). International evidence on music interventions was reviewed by Daykin (2012): these seek to improving health and social outcomes amongst young people by fostering expression, skills and confidence, building resilience and addressing problematic attitudes and beliefs.

Arts programming in youth justice settings around the world differs in type and scope. It is likely to be influenced by variations in penal policy that shape delivery, funding arrangements, experiences and outcomes from arts programmes. These include variations in the age of criminal responsibility, which ranges from age 7 in India and certain US states to 18 in Belgium (HAQ Centre for Child Rights). Different sentencing practices in different countries are also likely to influence arts provision. For example, in England, where the age of criminal responsibility is 10 years old, children between 10 and 17 years are dealt with by separate youth courts and are not sent to adult prisons. The scope and success of arts provision is also likely to be influenced by the political and cultural framing of youth crime and the extent to which different countries focus on welfare, retributive or restorative models of justice. For example, in the Finnish system, which focused on prevention, there is considerable overlap between the criminal justice system and the child welfare system resulting in joint decision‐making at a political and policy level (Marttunen, 2004). While there seems to be increasing interest in the use of arts programmes for young people in justice settings in several countries, evidence will vary in terms of scale and reporting practices.

### 2.2 The intervention

Arts interventions are diverse, and this review includes interventions focused on participant involvement in artistic and creative activities such as painting, sculpting, music, drama and dance. These types of arts interventions may be delivered as one‐off experiences or as a series of activities taking place over a few weeks, months or years. Arts participation may be delivered as an intervention on its own or as a ‘hook’ for other interventions, such as mentoring or education. Arts interventions may also use art as therapy (a form of psychotherapy) and as a medium to address emotional difficulties. Arts interventions will vary in terms of the settings in which they take place and will include those delivered in young offenders institutions or secure training centres (UK) juvenile correctional facilities (USA), prisons, other residential settings, dance and music studios, theatres and other community settings, schools and workplaces. Arts interventions can be delivered by a range of instructors and this review includes implementation by trained professionals, volunteers, and peers.

Examples of arts interventions included in this review are:

- Music making
- Arts and craft, that is, necklace making, decoupage
- Dancing
- Drama
- Film
- Podcasting
- Theatre
- Creative writing and poetry
- Photography
- Painting
- Pottery
- Sculpture
- New media/digital arts

### 2.3 How the intervention might work

Table 1 provides a preliminary logic model describing the potential chains of causes and effects of arts interventions on preventing offending and anti‐social behaviour (primary outcomes), and supporting secondary outcomes including attendance, educational attainment and psychological well‐being. It includes consideration of intermediary outcomes associated with the costs of arts interventions and of adverse events. Arts interventions are expected to bring about positive changes in primary and secondary outcomes through a combination of active ingredients including appropriate resources (inputs), planning and intervention design activities and delivery outputs. We consider funding models/imperatives to ensure that attention is paid to how these might impact on whether and how outcomes are successfully achieved and sustained. The logic model has been developed through discussion with the project Advisory Board and will be elaborated as the findings of the systematic review are reported and in further Advisory Board meetings. It is intended to inform future theory of change approaches to arts interventions for CYP at‐risk of offending or who have offended.

**Table 1:** A logic model for arts interventions for at risk and offending children and young people (8–25 years).

Resources	Planning and intervention	Outputs	Outcomes (to be measured in quantitative studies; qualitative studies to examine processes by which interventions achieve outcomes)	Impacts
Staffing, involvement of professional artists or artist educators and volunteers; supportive youth service staff; financial resources; appropriate facilities including in prisons, communities, youth justice settings; materials and equipment for arts. Appropriately designed activities (including therapeutic, educational, creative, and stand alone, integrated arts) for young people considering age, gender/sex, ethnicity, socioeconomic status, educational experiences, family and community context.	Operational factors in various settings. Programme planning: one‐off activities and activities of longer duration; pattern, regularity and consistency of delivery, codesigned programmes, sustainability, cultural relevance Project management, budget, programme planning, venue, facilities and equipment, staffing, training and supervision.	Arts‐based activities including instruction, learning and discussion workshops, performances, exhibitions.	Primary: Offending behaviour, for example, violence/aggression, weapon carrying/use, any other criminal activity (e.g., theft, drug offences); sexual offences, drug use/misuse; gang involvement; vandalism Anti‐social behaviours, for example, aggression, bullying, alcohol use/misuse, problem gambling, delinquency, victimisation/harassment Secondary and intermediate outcomes Participation/attendance at arts interventions Educational attainment, attendance and engagement (school), exclusions at school Psychological and emotional wellbeing (e.g., mood, self‐esteem, confidence, autonomy, social connections, loneliness, resilience) Costs and associated economic outcomes Adverse events (e.g., negative experiences and emotions associated with arts participation)	Longer term: Enhanced understanding of best practice, increased availability and access to include arts interventions for young people at risk and in contact with criminal justice settings and attention to sustainability of programmes. Wider awareness and understanding leading to policy change and systemic improvements in the justice system, education, social care and health.

### 2.4 Why it is important to do this review

Understanding what works in arts intervention programmes for preventing serious crime, violence and disruptive behaviours in CYP (8–25 years) at‐risk of offending or who have offended can support policy and intervention development. There is a need to develop an understanding of the effectiveness and impact of arts interventions on keeping CYP safe from involvement in violence and crime. Reviewing evidence on factors impacting the implementation of arts interventions, and barriers and facilitators to participation and achievement of intended outcomes is also important. This work, and the logic model proposed, can inform a theory‐of‐change approach to ensure the development of an evidence‐led framework of the processes by which arts interventions might work in preventing offending behaviours. This will support the translation of evidence into accessible, useful and useable information for a range of diverse stakeholders seeking to make decisions about arts interventions, young people and offending behaviour. In this way, the work will support policy and practice to prevent young people from becoming involved in violent crime.

To date, research has been characterised by a preponderance of small‐scale (limited number of participants, locally‐focused), short‐term studies that reveal the complexity of interventions and a variety of activities, styles and delivery formats (Anderson, 2010; Chen, 2016; Daykin, 2012). However, these are often not shared and have had limited impact on arts programming in the youth justice sector. There have been few attempts to synthesise evidence across art forms, regions and countries. This review is needed because, despite the plethora of arts interventions and associated evaluation studies, there is currently no existing up‐to‐date systematic review on the effects of a full range of arts interventions for CYP (8–25 years) at‐risk of offending or who have offended on behavioural (actions), psychosocial (emotional and cultural), cognitive (logic/thought) and offending outcomes. This review will help develop an understanding of the effectiveness of arts interventions in reducing risk and offending behaviours and build evidence on the contextual factors about how effective interventions can be best designed and implemented. It will provide an evidence‐led foundation for ongoing strategic decision‐making about young people, arts interventions and offending, informing policy development and practice guidelines.

## 3 OBJECTIVES

The proposed systematic review question is: What is the effectiveness of arts interventions for at‐risk and offending CYP (8–25 years)?

There are three objectives

- To evaluate evidence on the effectiveness and impact of arts interventions on keeping CYP safe from involvement in violence and crime.
- To synthesise evidence on factors impacting the implementation of arts interventions, and barriers and facilitators to participation and achievement of intended outcomes.
- To develop a logic model/theory‐of‐change approach to ensure the development of an evidence‐led framework of the processes by which arts interventions might work in preventing offending behaviours.

## 4 METHODS

### 4.1 Criteria for considering studies for this review

This review is based on the previously published protocol (Mansfield, 2023). This section, except for specifically mentioned updates or changes, draws on the protocol.

#### 4.1.1 Types of studies

We included randomised and non‐randomised controlled trials and quasi‐experimental study designs. We did not include quantitative studies that did not employ a control or comparator group. We included qualitative studies that were conducted alongside intervention trials that investigated the experiences and perceptions of participants, and that offered insight into the barriers and facilitators associated with delivering and receiving arts interventions. We included qualitative and mixed methods studies that were focused on the delivery of an arts intervention. We included those which explored aspects of the process of intervention delivery from the perspectives of those delivering and those who were participants in the intervention and/or their carers/family members or significant agents (e.g., probation officers). We included studies from any global setting.

#### 4.1.2 Types of participants

We included studies that included CYP (8–25 years) who were either identified as at‐risk of offending behaviour (secondary populations) or already in the criminal justice system (tertiary populations).

#### 4.1.3 Types of interventions

We included studies of interventions involving arts participation. Arts participation included involvement in artistic and creative activities. Studies which included arts participation as an intervention on its own or alongside other interventions, such as mentoring, were included. We included studies that used art as therapy (a form of psychotherapy) and as a medium to address emotional difficulties.

Examples of arts interventions in included studies are:

- Music making
- Arts and crafts, that is, necklace making, decoupage
- Dancing
- Drama
- Film
- Podcasting
- Theatre
- Creative writing and poetry
- Photography
- Painting
- Pottery
- Sculpture
- New media/digital arts/multimedia

We included studies that compared arts interventions to either no intervention, usual care, other types of arts intervention or non‐arts control. The intervention had to involve organised arts interventions targeted to the CYP population. We did not include associational studies between arts participation and offending behaviour.

#### 4.1.4 Types of outcome measures

Briefly describe the types of outcome measures that will be included and excluded.

##### Primary outcomes

List primary outcomes.

- Offending behaviour, for example, violence/aggression, weapon carrying/use, any other criminal activity (e.g., theft, drug offences); drug use/misuse; gang involvement, vandalism, sexual offences all including rates of recidivism, sexual and rearrests
- Anti‐social or pro‐social behaviours (e.g., aggression, bullying, alcohol use/misuse, problem gambling, delinquency, victimisation/harassment; sense of teamwork, belonging, worthwhileness, positive behaviours from engagement)

##### Secondary outcomes

List secondary outcomes.

- Participation/attendance at arts interventions
- Educational attainment, attendance and engagement (school), exclusions at school
- Workplace engagement
- Psychological and emotional wellbeing (e.g., mood, self‐esteem, confidence, autonomy, social connections, loneliness, resilience)
- Costs and associated economic outcomes
- Adverse events (e.g., negative experiences and emotions associated with arts participation)

Our review synthesised evidence on factors impacting the implementation of arts interventions, and barriers and facilitators to participation and achievement of intended outcomes.

#### 4.1.5 Duration of follow‐up

We considered outcomes at the following time points: short‐term immediately post‐intervention to <3 months; medium‐term 3 to <12 months post intervention, long‐term >1 year post intervention. Where studies reported multiple follow‐ups within a single time‐point range we preferentially extracted as follows: short‐term, the closest follow‐up point to the end of the intervention. Medium‐ and long‐term: the latest time point reported.

#### 4.1.6 Types of settings

We included studies employing arts interventions in any setting including (i) for example, youth offender institutions (UK) or juvenile correctional facilities (USA), prisons, other residential settings; (ii) community and workplace settings; and (iii) schools.

### 4.2 Search methods for identification of studies

#### 4.2.1 Electronic searches

We consulted Campbell guidance on searching for studies (Kugley, 2016). Our search strategy included expert advice from information services experts at Brunel University London Library. We searched key databases including AMED (via EBSCOHost NHS Open Athens), Academic Search Complete; APA PsycInfo; CINAHL Plus; ERIC; SocIndex; SportDiscus (via EbscoHost), Medline (via Ovid), CENTRAL, Web of Science, Scopus, PTSDPubs and Performing Arts Periodicals Database (via ProQuest), Sage, the US National Criminal Justice Reference Service, the Global Policing and the British Library EThOS databases, and the National Police Library to January 2023.

We used a combination of controlled vocabulary, that is, medical subject headings (MeSH), and free text terms to identify published articles. In addition, we checked reference lists of reviews and retrieved articles for additional studies. Search strategies can be found in Supporting Information: Appendices 1–9. We included separate search strings for identifying quantitative and qualitative studies. To identify the population of interest we used the search filter proposed by the Canadian Health Libraries Association (CHLA, 2022). We used and adapted the Cochrane highly sensitive search filter to identify RCTs (Higgins, 2021), a validated filter for identifying non‐randomised controlled studies (Waffenschmidt, 2020) and the University of Texas School of Public Health (University of Texas, 2022) filter for identifying qualitative studies which have been demonstrated to show good performance in sensitivity and specificity (Wagner, 2020).

Our searches were worldwide and included studies from any country. We agreed the provision and support for translation of potentially relevant papers into English.

Titles and abstracts were independently screened by two reviewers to identify potential sources of disagreement. These were discussed and reviewed by a third senior author in the team. Two reviewers then screened the full texts of potentially relevant studies and applied the inclusion and exclusion criteria, with recourse to a third reviewer for any records where there was uncertainty.

We checked the reference lists of relevant systematic reviews found in our searches.

#### 4.2.2 Searching other resources

In searching other sources we sought expert advise. We worked with an expert Advisory Board convened by Campbell and the Youth Endowment Foundation for this purpose. We searched the WHO International Clinical Trials Registry Portal (ICTRP). We conducted a grey literature search of databases such as Arts and Humanities Citation Index and ProQuest using our search terms. In discussion and agreement with our Advisory Board, we conducted a selected website search including the UK's National Criminal Justice Arts Alliance and other websites with a specific focus on CYP and the criminal justice system. We conducted an Advanced Google Scholar search and sifted the first 100 returns using search terms from our search strategy as appropriate. We included reports that met our inclusion criteria. We used our inclusion and exclusion criteria to select grey literature. We revised our grey literature search alongside advice from experts on the project Advisory Board.

### 4.3 Data collection and analysis

#### 4.3.1 Description of methods used in primary research

The interventions involved organised arts interventions targeted to the population. We did not include associational studies between arts participation and offending behaviour. We included controlled study designs. Studies that compared arts interventions to either no intervention, usual care, or other types of arts intervention or non‐arts control were included. We included studies comparing one type of arts intervention to another. Studies using any qualitative research method to examine the context, intervention assumptions, implementation process (including barriers and facilitators), and mechanisms of impact and outcomes were included. Qualitative studies evaluating how an arts intervention works could be conducted as independent studies or alongside controlled study designs.

#### 4.3.2 Selection of studies

Two review authors independently assessed the titles and abstracts of potential included studies identified by the search strategy for their eligibility. We obtained the full text of studies we considered potentially eligible, or if the eligibility of a study was unclear from the title and abstract. We excluded studies that did not match the inclusion criteria (see ‘criteria for considering studies for this review’). We resolved disagreements between review authors regarding inclusion by discussion. If agreement could not be reached, a third review author assessed relevant studies, and a majority decision was made. We did not anonymise studies before the assessment.

#### 4.3.3 Data extraction and management

Two review authors independently extracted data from all included studies using a standardised and piloted data extraction form. They resolved discrepancies and disagreements by consensus. In cases where consensus could not be achieved, a third review author assessed the article, and a majority decision was made.

We extracted the following data from quantitative studies included in the review:

Study characteristics (aims/objectives, study design, sample size, description of the sample, country, recruitment years and procedure, conflict of interest, funding source).

Characteristics of the participants (gender/sex, age, ethnicity, socioeconomic status, education, at risk or in contact/conflict with the criminal justice system).

Description of the interventions (experimental and control), context and setting, country/location, intervention assumptions/theoretical framing, implementation processes (human and financial resources), fidelity, dose, adaptation, reach, mechanisms of impact (participant response, mediators, unanticipated consequences).

Data collection methods including duration and timing of follow‐up/outcome assessment.

Results as outcome measures of interest to this review, including details of measurement scales and analysis methods.

Risks and biases.

Discussion including interpretations by authors, limitations and implications.

We extracted the following data from qualitative studies in the review:

Study characteristics and context (aims/objectives study design, sampling approach, description of the sample, country, recruitment years and procedure, conflict of interest, funding source).

Characteristics of the participants (gender/sex, age, ethnicity, socioeconomic status, education, at risk or in contact/conflict with the criminal justice system).

Description of the interventions, context and setting, country/location, theoretical framing, implementation processes (human and financial resources), processes of impact (funding context, design and delivery model, participants' responses, unanticipated consequences).

Data collection methods.

Findings as qualitative themes/processes including analysis methods.

Methodological limitations.

Discussion including interpretations by authors and implications.

As arts interventions are complex we extracted detailed information regarding the intervention guided by the MRC guidance on process evaluations of complex interventions (Moore, 2015) and items on the TiDiER (Hoffman, 2014) and Cert (Slade, 2016) checklists framed by a focus on why, what, who, where, when, how much, how well, tailoring and modifications.

#### 4.3.4 Assessment of risk of bias in included studies

Two reviewers independently assessed the risk of bias or study quality of included studies. We used the Cochrane Risk of Bias tool (Higgins, 2011) to evaluate included controlled trials. We assessed the following domains for each study: *Random sequence generation; Allocation concealment; Blinding of participants and providers; Blinding of assessors; Incomplete outcome data; Selective reporting*. For cross‐over studies only we assessed the domain ‘*free from carry‐over effects*’.

We took a risk‐to‐rigour approach to evaluate qualitative studies (Noyes, 2018) using the CASP tool for qualitative research (CASP UK) to appraise the rigour and significance of the sampling, data, collection, analysis and reporting of results.

#### 4.3.5 Measures of intervention effect

For continuous outcome measures we expressed the size of the intervention effect using the mean difference (MD) when all studies utilised the same measurement scale, or the standardised mean difference (SMD) when studies used different scales, with 95% CIs. If pooling from different scales for which the direction of interpretation varied, we planned to normalise the direction of the scales to a common direction. To aid the interpretation of the pooled effect size, we planned to back‐transform the SMD to the most commonly used outcome scale on the basis of the median standard deviation from trials using that scale when possible.

For dichotomous outcomes we planned to report the Relative Risk, Odds Ratio or Risk Difference where available from individual included studies. In the event that we pooled data in a new analysis, we planned to preferentially report the relative risk as the effect size of interest but to also report the risk difference.

#### 4.3.6 Unit of analysis issues

Unit‐of‐analysis issues refer to issues regarding clustering (individuals randomised/allocated in clusters), cross‐over designs, and studies with multiple outcome measurement time points.

For studies with more than two eligible active treatment groups that are included in a meta‐analysis as separate interventions, we planned to divide the number of participants in the control group between active treatment groups, to avoid double counting (Higgins, 2021). For cluster RCTs, we planned to seek direct estimates of the effect from an analysis that accounted for the cluster design. When the analysis in a cluster trial does not account for the cluster design, we planned to use the approximately correct analysis approach, presented in the Cochrane Handbook (Higgins, 2021). For cross‐over studies, we planned to only include data from the first phase of the study, when they are available due to the risk of carryover effects. However, as first‐phase, or phase‐by‐phase data were not available for any of the included cross‐over studies we took the decision to analyse these studies as presented.

#### 4.3.7 Criteria for determination of independent findings

Where we identify multiple reports for a single study we only included data from that study once in any given analysis. Where a study reported multiple outcome domains with some conceptual overlap that fit one of our stated outcome domains, the research team agreed which of the measures conceptually best matched our outcome of interest and only included that measure. This decision was not made on the basis of the results of these outcomes.

#### 4.3.8 Dealing with missing data

Where there were insufficient data presented in the study report to enter into an analysis, we requested the missing data from the study authors. We planned to preferentially calculate and extract effect sizes derived from intention‐to‐treat analyses. We evaluated the potential risk of bias introduced by missing data in our assessment of risk of bias, within the domain ‘Incomplete Outcome Data’ and planned to explore the impact of risk of bias through sensitivity analyses.

#### 4.3.9 Assessment of heterogeneity

We planned to deal with heterogeneity by only combining studies that examine similar interventions. To estimate statistical heterogeneity, we would calculate the *χ*² statistic, the between‐study variance (*τ* ^2^) and the proportion of this variance not due to sampling error (*I*²). We planned to use these measures, together with a visual inspection of the forest plots to form judgements about heterogeneity. If we identified substantial heterogeneity, we planned to report it and explore possible causes by prespecified subgroup analysis.

#### 4.3.10 Assessment of reporting biases

We planned to consider the potential influence of small study biases on review findings. We planned to use funnel plots to visually explore small study biases where there were at least 10 included studies in a meta‐analysis.

#### 4.3.11 Data synthesis

We conducted separate analyses of the quantitative evidence for the following comparisons: Arts intervention versus no intervention or usual practice; Arts interventions versus non‐arts control; Arts interventions versus other arts intervention.

We planned to pool studies of arts interventions in the primary analysis, including different types of arts, delivery modes and settings using a random‐effects model to account for the anticipated heterogeneity between studies. For each comparison of interest, we planned to conduct separate analyses at short, medium and long‐term follow‐up. For the primary analysis, we planned to pool data from studies regardless of the specific population. Where there were inadequate data to enable statistical pooling we planned to conduct a narrative synthesis of the evidence. For head‐to‐head comparisons of different types of arts intervention, we would only pool studies if the intervention and comparators are conceptually similar.

In the event that we conducted a narrative synthesis we planned to separately synthesise studies within the comparisons outlined above, guided by the SWiM guideline (Campbell, 2020). We planned to first summarise all arts interventions in the primary synthesis, including different types of arts, delivery mode and setting and explore the potential heterogeneity of treatment effects between studies by considering intervention settings (custodial, community or school‐based interventions) and population age (children and adolescents 8–18 years; young adults 18–25 years). We reported effect sizes for each reported outcome of interest with estimates of precision where available. We included all relevant studies for each comparison and outcome and documented the size and risk of bias of those studies in our reporting. We did not plan to present this synthesis in a tabular or graphical format.

See below for details of the synthesis of qualitative research.

#### 4.3.12 Subgroup analysis and investigation of heterogeneity

##### State the potential effect modifiers with rationale for each, if moderator analysis (subgroup or meta‐regression analyses) will be performed

Where there were adequate data and significant heterogeneity is observed in a meta‐analysis (*I* ^2^ ≥ 50%, *p* < 0.10), we planned to explore subgroup analyses of quantitative results by type of intervention. To explore whether there is a difference in effects between subgroups, we planned to use the test for subgroup differences (Deeks, 2020).

We prespecified the following subgroups.

Intervention setting: Custodial, community or school‐based interventions.

Population Age: Children and adolescents 8–18 years, young adults 18–25 years.

We also intended to conduct an inductive approach to narratively exploring other potentially important sources of heterogeneity, for example, group versus individual therapy, the use of specific intervention characteristics such as incentives to participation, and types of offending (e.g., violent, sexual, non‐violent).

#### 4.3.13 Sensitivity analysis

When sufficient data were available, we planned to explore the impact of risk of bias for the primary analyses, by repeating the analyses and excluding studies rated at high risk of bias.

#### 4.3.14 Treatment of qualitative research

We took a thematic approach to analysing and synthesising data from qualitative studies. This included line‐by‐line reading for extraction and preliminary coding, development of descriptive themes and refinement of analytical themes (Thomas, 2008). We conducted our thematic analysis with attention to the complexity of arts interventions for CYP at risk of offending or in the criminal justice system. We mapped themes from the findings of the qualitative studies to theoretical domains of complexity relating to the intervention itself, the population, the implementation of the intervention and the specific context that may impact the process of delivering and engaging with the interventions. Table 2 outlines the complexity framework for qualitative analysis. We took a reflexive approach and consulted with Advisory Board to seek advice about the relevance of themes for policy and practice.

**Table 2:** Framework for qualitative analysis.

Complexity domain	Potential components
Intervention complexity	Providers, Theoretical model/assumptions, Type of art, Delivery mode/setting, Time/equipment/costs, accessibility, youth‐focused
Contextual complexity	Residential status, family/carer/community support, Socioeconomic factors
Population/personal complexity	Secondary or tertiary population, values and choices, demographics, culure
Implementation complexity	Mode of delivery, Fidelity of intervention, Adherence, Local support structures

#### 4.3.15 Summary of findings and assessment of the certainty of the evidence

We used the GRADE system to rank the level of certainty of the evidence (Schünemann, 2020). The GRADE approach uses five considerations (risk of bias, consistency of effect, imprecision, indirectness, and publication bias) to assess the certainty of the body of evidence for each outcome, and uses the following criteria to describe the confidence in the evidence:

- high – we are very confident that the true effect lies close to that of the estimate of the effect
- moderate – we are moderately confident in the effect estimate; the true effect is likely to be close to the estimate of effect, but there is a possibility that it is substantially different
- low – our confidence in the effect estimate is limited; the true effect may be substantially different from the estimate of the effect
- very low – we have very little confidence in the effect estimate; the true effect is likely to be substantially different from the estimate of the effect.

We decreased the grade rating by one (−1), two (−2), or three (−3) levels, up to a maximum of −3, (or very low) for any criteria, based on the level of concern it raised.

To assess the methodological quality of the included qualitative studies, two review authors independently applied the CASP quality checklist for each qualitative study. The checklists were used to indicate if a specific study had been well‐designed, appropriately carried out and properly analysed. We then employed the CERQual schema (Confidence in the Evidence of Reviews of Qualitative Research) for judging how much confidence could be placed in the overall review findings developed through the synthesis. CERQual has four components (methodological limitations, relevance adequacy and coherence) and uses the following criteria for judging confidence in the body of literature (Lewin et al., 2015).

Methodological limitations – the extent to which there are problems in the design or conduct of primary studies that contributed evidence to the review.

Relevance – the extent to which evidence in the primary study is applicable (perspective, population, phenomenon of interest, setting).

Coherence – the degree to which primary studies provide a convincing explanation for patterns.

Adequacy – the degree of richness and quantity/scope of data

Confidence was decreased if there were serious or very serious limitations in the design or conduct of the study, the evidence was not relevant to the study objectives, the findings/conclusions were not supported by the evidence, or the data were of inferior quality and inadequate in supporting the findings. Confidence was increased if the study was well‐designed with few limitations, the evidence was applicable to the context specified in the objectives, the findings/conclusions were supported by evidence and provided a convincing explanation for any patterns found or the data supporting findings were rich and of high quality.

## 5 RESULTS

### 5.1 Description of studies

#### 5.1.1 Results of the search

**Figure 1:**
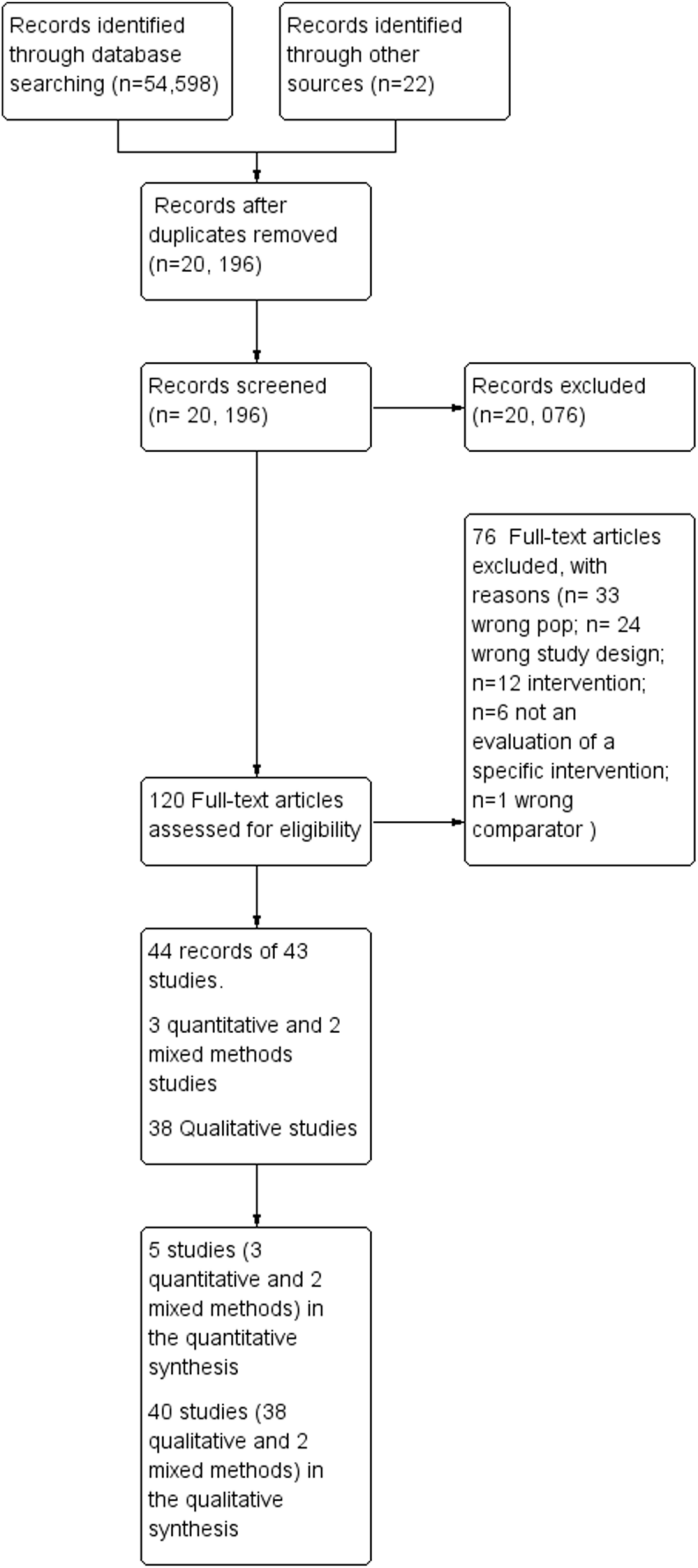
Flow diagram of the search screening process.

See Figure 1 for a summary of the search screening process. There were 54,598 initial records from database searches and 22 from other sources (reference searches and experts' contributions). After de‐duplication 20,196 records were retained. Forty‐four records of 43 studies met our eligibility criteria (3 quantitative, 38 qualitative and 2 mixed methods).

#### 5.1.2 Included studies

##### Quantitative studies

Five studies (3 quantitative and 2 mixed methods) were included in the quantitative synthesis (Anderson, 2010; Bittman, 2009; Caulfield, 2022; DeCarlo, 2004; Tyson, 2002) that combined included 304 participants.

##### Qualitative studies

40 studies (38 qualitative and 2 mixed methods) were included in the qualitative synthesis (Anderson, 2010; Atherton, 2022; Baker, 2007; Barrett, 2015; Bowey, 2006; Caulfield, 2022; Cesar, 2020; Chong, 2020; Clennon, 2015; Daykin, 2017; de Roeper, 2009; Flores, 2016; Fullchange, 2018; Gann, 2010; Gowland‐Pryde, 2016; Hadland, 2010; Hanrahan, 2017; Hickey, 2018; Howard, 2022; Jordan, 2015; Lazzari, 2005; Lea, 2019; Lotter, 2015; Massó‐Guijarro, 2020; Morgan, 2020; Nicklin, 2017; Parker, 2018; Podkalicka, 2009; Pope, 2022; Ruggiero, 2013; Seroczynski, 2011; Tett, 2012; Thompson, 2015, 2022; Varley, 2019; Winn, 2010, 2011; Zlotowitz, 2016) that included approximately *n* = 620 participants. Five studies either did not report participant numbers or not report them accurately (Hickey, 2018; Lea, 2019; Podkalicka, 2009; Thompson, 2015; Winn, 2010).

#### 5.1.3 Country of origin and setting

##### Quantitative studies

Three quantitative studies were conducted in the UK (Anderson, 2010; Bittman, 2009; Caulfield, 2022) and 2 in the USA (DeCarlo, 2004; Tyson, 2002). These studies were conducted in a Young Offenders Institution (Anderson, 2010), a secure residential children's home (Bittman, 2009), a Young Offenders service (Caulfield, 2022), a residential youth services centre (Tyson, 2002) and an urban classroom environment (DeCarlo, 2004).

##### Qualitative studies

Most of the qualitative research was undertaken in the UK and the US. Sixteen qualitative studies were conducted in the UK (Anderson, 2010; Atherton, 2022; Bowey, 2006; Caulfield, 2022; Clennon, 2015; Daykin, 2017; Gowland‐Pryde, 2016; Hadland, 2010; Hanrahan, 2017; Howard, 2022; Morgan, 2020; Parker, 2018; Tett, 2012; Varley, 2019; Zlotowitz, 2016). Sixteen were conducted in the USA (Baker, 2007; Cesar, 2020; Fullchange, 2018; Gann, 2010; Hickey, 2018; Jordan, 2015; Lazzari, 2005; Lea, 2019; Nicklin, 2017; Pope, 2022; Ruggiero, 2013; Seroczynski, 2011; Thompson, 2015, 2022; Winn, 2010, 2011). Four studies were conducted in Australia (Barrett, 2012, 2015; de Roeper, 2009; Podkalicka, 2009) with two studies conducted in South Africa (Flores, 2016; Lotter, 2015) and one study conducted in Spain (Massó‐Guijarro, 2020), and in South Korea (Chong, 2020).

Studies were conducted in a range of custodial and non‐custodial settings. There were 13 studies in prison or juvenile/young offenders detention settings (Anderson, 2010; Atherton, 2022; Baker, 2007; Barrett, 2012; Clennon, 2015; Daykin, 2017; Hickey, 2018; Lazzari, 2005; Lea, 2019; Tett, 2012; Thompson, 2015, 2022; Winn, 2010). Other settings include community youth services (Bowey, 2006; Caulfield, 2022; de Roeper, 2009; Gowland‐Pryde, 2016; Hadland, 2010; Hanrahan, 2017; Howard, 2022; Lotter, 2015; Massó‐Guijarro, 2020; Morgan, 2020; Podkalicka, 2009; Varley, 2019; Zlotowitz, 2016), education setting, for example, schools, colleges or universities (Barrett, 2015; Chong, 2020; Gann, 2010; Jordan, 2015; Nicklin, 2017; Parker, 2018; Seroczynski, 2011) and activity camps (Cesar, 2020; Fullchange, 2018; Ruggiero, 2013). One study took place in a young adult problem‐solving court (Pope, 2022) and another was conducted in a care setting (Flores, 2016).

#### 5.1.4 Study design

##### Quantitative studies

Three studies (Anderson, 2010; Caulfield, 2022; DeCarlo, 2004) were non‐randomised comparative studies with a parallel design, and 2 (Bittman, 2009; Tyson, 2002) were described as randomised studies of which Bittman (2009) employed a cross‐over design and Tyson (2002) a parallel design.

##### Qualitative studies

Twenty‐four studies used only one qualitative method for data collection. Of these, 11 employed ethnographic observation methods (Baker, 2007; Caulfield, 2022; Clennon, 2015; Gann, 2010; Howard, 2022; Massó‐Guijarro, 2020; Podkalicka, 2009; Winn, 2010, 2011; Zlotowitz, 2016). One study used auto ethnography (Thompson, 2015). Twelve studies employed interview techniques (Anderson, 2010; Atherton, 2022; Bowey, 2006; Cesar, 2020; Chong, 2020; Fullchange, 2018; Hadland, 2010; Hanrahan, 2017; Jordan, 2015; Parker, 2018; Varley, 2019). One study used focus groups (Tett, 2012).

Fifteen studies used mixed qualitative techniques. Of these, seven studies employed a mixture of observations and interviews (Barrett, 2012, 2015; de Roeper, 2009; Lazzari, 2005; Lea, 2019; Lotter, 2015; Pope, 2022). One study used a combination of feedback sheets, focus groups and interviews (Hickey, 2018). Four studies combined observations, interviews and focus groups (Daykin, 2017; Flores, 2016; Morgan, 2020; Thompson, 2022). Two studies used interviews, observations and journaling (Gowland‐Pryde, 2016; Nicklin, 2017), and one study each employed a mix of interviews and journaling (Ruggiero, 2013), reading, journaling and discussion groups (Seroczynski, 2011).

Four studies used mixed qualitative and quantitative methods. We included only the qualitative component from two of these (Varley, 2019; Gann, 2010) as the quantitative component did not meet our inclusion criteria (no control condition/group). We included the qualitative component and the quantitative component from Anderson (2010) and Caulfield (2022) but report only the qualitative findings in this section.

#### 5.1.5 Participants

##### Quantitative studies

Included participants were described as young offenders (Anderson, 2010; Caulfield, 2022), adolescent and teen residents of a secure children's home (Bittman, 2009), African American adolescents in an urban classroom (DeCarlo, 2004) and ‘Runaway, abused, abandoned, neglected, truant, and youth who are otherwise homeless’ (Tyson, 2002).

Two studies (Anderson, 2010; DeCarlo, 2004) included exclusively male participants. The three remaining studies included both male and female participants. Of the total number of recruited participants where sex was reported (*n* = 156), 87% were male and 13% were female. Mean age ranged from 14 to 18.2 years across studies with the youngest participant 12 and the oldest 22 years old.

Two UK‐based studies did not report information on the ethnicity of participants (Anderson, 2010; Caulfield, 2022). For the US studies, one (Bittman, 2009) simply described including African American, Asian, Caucasian and Puerto Rican participants without reporting specific numbers from each. DeCarlo (2004) only described their sample as representative of the community's demographic characteristics and one described including African American (55%), Hispanic (27%) and White (18%) participants.

Two US‐based studies reported further context regarding participants. Bittman (2009) reported that some participants had mental health disorders including but not limited to oppositional defiance disorder, post‐traumatic stress disorder, separation anxiety, mood disorder, depression disorder, anxiety disorder, ADHD, parent/child relational disorder, conduct disorder, cognitive disorder, panic disorder, and substance/alcohol abuse, and that participants would be placed in the secure home for reasons including running away, out‐of‐control behaviour, truancy, anger management, inappropriate sexual behaviour, aggression, abuse/neglect, suicidal ideation, substance abuse, vandalism, and assault. Tyson (2002) reported that some youth in the setting will have been exposed to some form of abuse or adversely affected by parents with addiction or other issues and that participants may have had or had minor involvement with the Department of Juvenile Justice, and most had current cases with the Department of Children and Families.

##### Qualitative studies

A wide range of terminology was used to describe study populations in the qualitative studies. Participants were described as inmates (Atherton, 2022), prisoners (Tett, 2012), young offenders or young people who have offended (Anderson, 2010; Caulfield, 2019, 2022; Ruggiero, 2013; Varley, 2019), juveniles or juvenile offenders (Barrett, 2012; Baker, 2007; Gowland‐Pryde, 2016; Jordan, 2015; Nicklin, 2017), youth in or associated with the justice system (Cesar, 2020; Chong, 2020), young people in custodial and community supervision (Daykin, 2017), children in care (Flores, 2016), young people in need (Zlotowitz, 2016), young people ‘at risk’ (de Roeper, 2009; Bowey, 2006; Gann, 2010; Howard, 2022; Parker, 2018; Podkalicka, 2009), youth in the justice system (Thompson, 2022), young people with low aspiration (Clennon, 2015), young people excluded from school (Hadland, 2010), court detailed juveniles (Hickey, 2018); delinquent youth (Seroczynski, 2011), young detainees or detained youth (Lazzari, 2005; Thompson, 2015), formerly incarcerated youth (Lea, 2019; Winn, 2010, 2011), vulnerable (Massó‐Guijarro, 2020), marginalised young people (Hanrahan, 2017; Morgan, 2020); young adults committing non‐violent crime (Pope, 2022); disadvantaged children (Barrett, 2015); probation participants (Fullchange, 2018); adolescent therapy clients (Lotter, 2015).

Fifteen studies did not report participant sex/gender (Barrett, 2012, 2015; Bowey, 2006; Caulfield, 2019; Chong, 2020; Flores, 2016; Gann, 2010; Hickey, 2018; Howard, 2022; Lotter, 2015; Nicklin, 2017; Podkalicka, 2009; Pope, 2022; Ruggiero, 2013). Of those reporting sex/gender of participants, the majority (*n* = 14) included both male and female included male and female participants (Atherton, 2022; Caulfield, 2022; Cesar, 2020; Daykin, 2017; de Roeper, 2009; Gowland‐Pryde, 2016; Hadland, 2010; Hanrahan, 2017; Jordan, 2015; Massó‐Guijarro, 2020; Parker, 2018; Seroczynski, 2011; Thompson, 2022; Zlotowitz, 2016). Eight studies included exclusively male participants (Anderson, 2010; Baker, 2007; Clennon, 2015; Fullchange, 2018; Lea, 2019; Morgan, 2020; Tett, 2012; Varley, 2019). Three studies included exclusive female participants (Lazzari, 2005; Winn, 2010, 2011).

Of the total number of recruited participants where sex/gender was reported clearly and accurately (*n* = 12/14 qualitative studies) (*n* = 300), 66% (*n* = 197) were male and 34% (*n* = 103) were female.

Age of participants ranged from the youngest 7 years to the oldest 25 years across included qualitative studies where age was reported. Eight studies did not report age (Atherton, 2022; Massó‐Guijarro, 2020; Nicklin, 2017; Podkalicka, 2009; Tett, 2012; Thompson, 2015; Winn, 2010, 2011).

Twenty‐five studies did not report on the ethnicity of participants (Anderson, 2010; Atherton, 2022; Barrett, 2012, 2015; Bowey, 2006; Caulfield, 2019, 2022; Cesar, 2020; Chong, 2020; Clennon, 2015; Daykin, 2017; de Roeper, 2009; Flores, 2016; Hadland, 2010; Howard, 2022; Lotter, 2015; Nicklin, 2017; Parker, 2018; Podkalicka, 2009; Pope, 2022; Ruggiero, 2013; Tett, 2012; Thompson, 2022; Varley, 2019).

A total of fifteen studies reported information on the ethnicity of participants. Ten of these studies used a range of simplified descriptions only including multi‐ethnic (Lazzari, 2005), Black (Baker, 2007; Lea, 2019; Thompson, 2015; Winn, 2010, 2011), African American and Caucasian (Gann, 2010) White and Roma (Massó‐Guijarro, 2020), White (Morgan, 2020), and ethnically diverse (Gowland‐Pryde, 2016). Five of these studies reported descriptive numerical data on ethnicity. Hickey (2018) described participants as 84% Black, 12% Latino, and 3% White. Seroczynski (2011) described participants as Caucasian American, 37.9% (*n* = 11) were African American, 6.9% (*n* = 2) were Hispanic American, and 13.8% (*n* = 4) were multiracial. Zlotowitz (2016) described residents as White (60%) and as being from Black and ethnic minority groups (40%). Fullchange (2018) described participants as Latino (*n* = 5), *n* = 1 as Black, and *n* = 1 as American Indian and White. Hanrahan (2017) described participants as British with a mixed ethnic profile: two were mixed race, two were Black.

Three studies provided further contextual information regarding participants, mentioning experiences such as school exclusion and additional challenging life experiences, such as unstable home environments, poverty, domestic violence, substance misuse, and involvement with the criminal justice system (Hanrahan, 2017), difficulties with education and traumatic life events (Lea, 2019), and severe emotional, social and behavioural difficulties and exposure to emotional, physical and/or sexual abuse and/or neglect (Flores, 2016).

#### 5.1.6 Interventions

##### Quantitative studies

All 5 quantitative studies evaluated a music‐based intervention. Anderson (2010) delivered either music classes or sculpture classes, Bittman (2009) evaluated a recreational music‐making programme, Caulfield (2022) evaluated a music programme including composition, production and performance skills and musical tuition, DeCarlo (2004) evaluated ‘Rap Therapy’, which used group listening and discussion of rap music, though not music‐making and similarly, Tyson (2002) evaluated ‘Hip‐hop Therapy’ in which participants were taught history of hip‐hop music, listened to rap music and engaged participants in group discussion of themes.

Four studies (Anderson, 2010; Bittman, 2009; DeCarlo, 2004; Tyson, 2002) delivered the interventions in groups over multiple sessions. Of these two reported group sizes of 8–10 (Anderson, 2010) or 6–12 (Bittman, 2009) with the other two studies not reporting group size. Caulfield (2022) delivered the intervention on a 1 to 1 basis.

All studies delivered their intervention over a number of sessions and a period of weeks. The number of sessions a week ranged from 1 to 3, and the number of weeks from 4 to 12.

##### Comparison groups

Two studies (Anderson, 2010; Bittman, 2009) reported using a control group of usual practice. This was described as classes in numeracy and maths or communication and literacy (Anderson, 2010) or not further detailed (Bittman, 2009). Caulfield (2022) compared outcomes in the intervention group with a cohort of children who did not attend the programme. DeCarlo (2004) included a control group who received ‘traditional psycho‐educational group therapy’ and Tyson (2002) included a control group who were instructed to concentrate on work efforts, self‐enhancement and peer relations.

#### 5.1.7 Outcomes

##### Primary outcomes

None of the quantitative studies reported offending behaviour.

For the outcome domain ‘anti or prosocial behaviour’, Anderson (2010) reported the number of behavioural incidents reported in the Young Offenders institution, Caulfield (2022) measured attitudes and behaviour using the Youth Music Attitudes and Behaviour Scale and Tyson (2002) reported peer relations, using the Index of Peer relations scale.

##### Secondary outcomes

Two studies (Anderson, 2010; Caulfield, 2022) reported attendance at the arts intervention.

Measures of psychological and emotional well‐being were reported by 4 studies (Anderson, 2010; Bittman, 2009; Caulfield, 2022; Tyson, 2002). Of these, Anderson (2010) measured self‐esteem using the Rosenberg Self‐Esteem Scale, and Locus of control using the Locus of Control Behavioural Scale. Bittman (2009) measured psychopathology with the Adolescent Psychopathology Scale, Anger using the Adolescent Anger Rating Scale and Depression using the Reynolds Adolescent Depression Scale. Caulfield (2022) measured well‐being using the Youth Music Well‐being Scale and Tyson (2002) measured self‐concept using the Self‐Concept Scale for Children.

Two studies used multidimensional composite outcome measures. Bittman (2009) used the Child and Adolescent Functional Assessment Scale and DeCarlo (2004) used the RAP therapy assessment scale. No studies reported measures of our specified outcomes educational engagement/attainment, workplace engagement, economic outcomes or adverse events.

##### Qualitative studies

Music, on its own or in combination with other art forms, was the most commonly reported intervention. Nineteen qualitative studies evaluated music‐based interventions. These included music technology (Clennon, 2015), music composition – rap (Hickey, 2018; Thompson, 2015), lyric writing, composing beats, recording, and/or performing music (Parker, 2018), songwriting, lyric writing and video recording (Massó‐Guijarro, 2020), variety of media and arts activities (Morgan, 2020), musical composition and computer‐based music sequencing (Baker, 2007), digital music, rapping and spray‐painting (de Roeper, 2009), Rap Therapy and Hip Hop Therapy (Gann, 2010), learning to play a (classical) musical instrument (Tett, 2012; Thompson, 2022) DJ‐ing and lyric writing (Zlotowitz, 2016), learning music or music‐making (Barrett, 2012, 2015; Caulfield, 2022; Daykin, 2017; Flores, 2016), and music therapy (Chong, 2020; Lotter, 2005, 2015).

Nine studies evaluated mixed arts interventions including visual arts, music production, dance and drama (Howard, 2022), paintings, sculptures, poems (Lazzari, 2005), writing, poetry, and music (Lea, 2019), recording original songs, producing artworks and radio content (Podkalicka, 2009), music theory, instrument and singing lessons (Anderson, 2010), arts drop‐in programme (Gowland‐Pryde, 2016) and a range of creative arts opportunities (Atherton, 2022; Caulfield, 2019; Hadland, 2010).

Eleven studies evaluated drama, theatre, literary or digital creation programmes (Bowey, 2006; Cesar, 2020; Fullchange, 2018; Jordan, 2015; Hanrahan, 2017; Nicklin, 2017; Pope, 2022; Seroczynski, 2011; Varley, 2019; Winn, 2010, 2011). One study evaluated a video game intervention (Ruggiero, 2013).

Twenty‐three studies reported interventions that were delivered to groups of participants over multiple sessions (Anderson, 2010; Atherton, 2022; Barrett, 2012, 2015; Bowey, 2006; Cesar, 2020; Clennon, 2015; Daykin, 2017; Flores, 2016; Fullchange, 2018; Gowland‐Pryde, 2016; Hadland, 2010; Hanrahan, 2017; Hickey, 2018; Howard, 2022; Jordan, 2015; Thompson, 2015, 2022; Varley, 2019; Winn, 2010, 2011; Zlotowitz, 2016). Fifteen studies included individual as well as group activities (Baker, 2007; Caulfield, 2019; Chong, 2020; de Roeper, 2009; Gann, 2010; Lazzari, 2005; Lea, 2019; Massó‐Guijarro, 2020; Morgan, 2020; Nicklin, 2017; Parker, 2018; Podkalicka, 2009; Pope, 2022; Ruggiero, 2013; Seroczynski, 2011; Tett, 2012). One study included one participant working as an individual in music therapy sessions (Lotter, 2015) and one study included 1:1 music‐making sessions (Caulfield, 2022).

All studies delivered their intervention over a number of sessions and a period of weeks, months or years. The duration of interventions programmes ranged from 6 weeks to 5 years.

#### 5.1.8 Excluded studies

There are 76 Excluded studies. Reasons for exclusion included incorrect population, study design, intervention, outcome and/or comparator.

### 5.2 Risk of bias in included studies

#### 5.2.1 Quantitative studies

**Figure 2:**
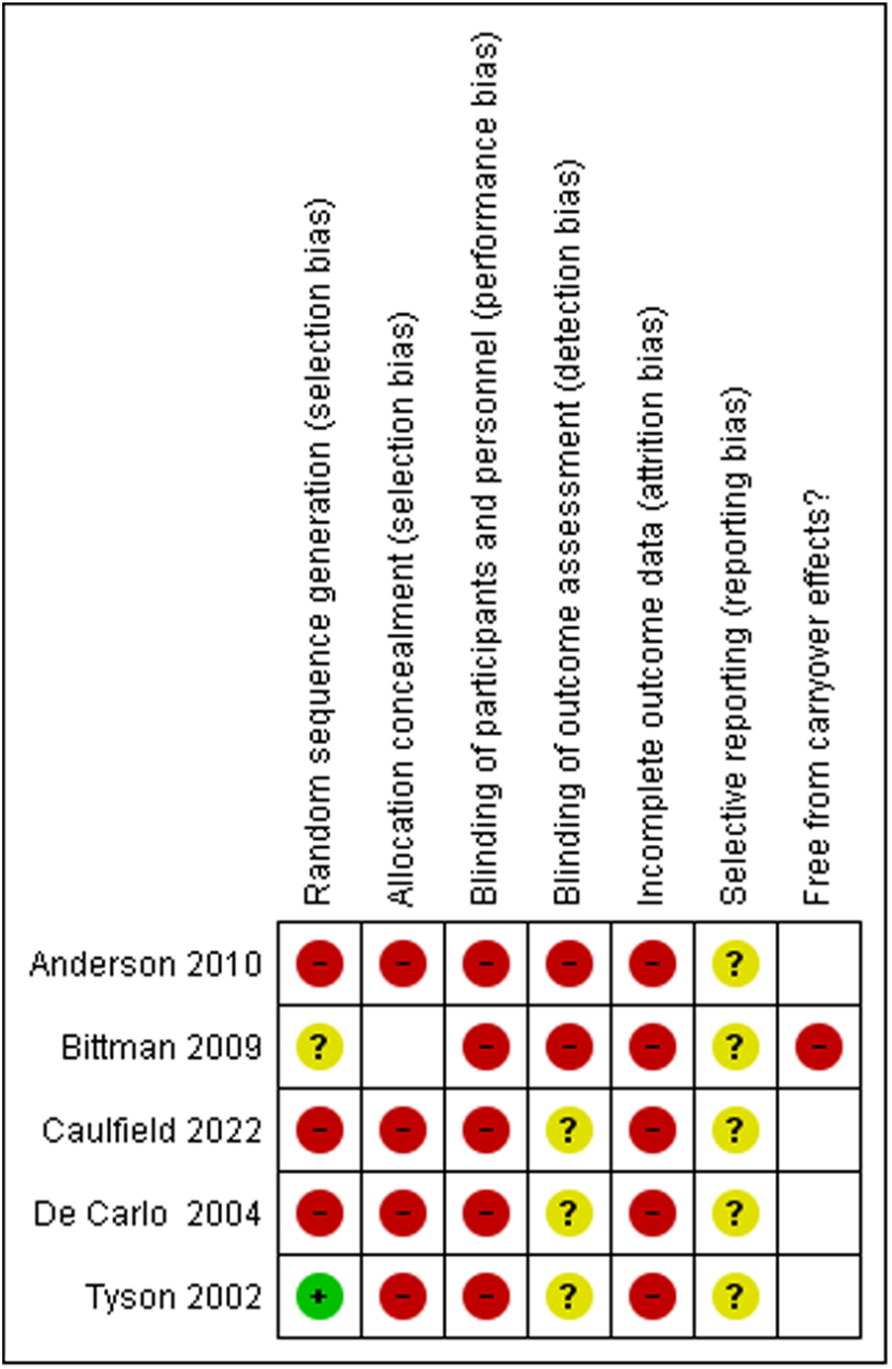
Risk of bias summary: Review authors' judgements about each risk of bias item for each included study.

**Figure 3:**
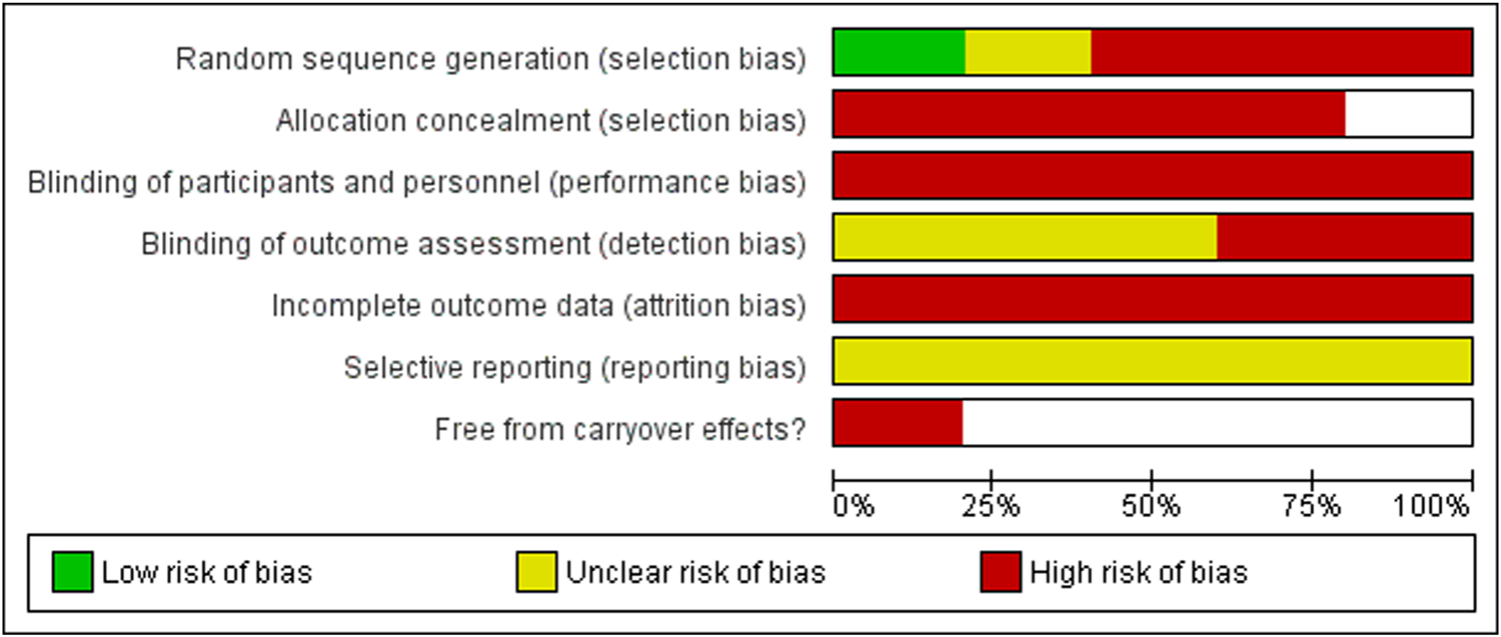
Risk of bias graph: Review authors' judgements about each risk of bias item presented as percentages across all included studies.

See Figures 2 and 3 for a summary of the risk of bias evaluation for quantitative studies. All 5 studies were rated at high risk of bias overall due to being at high risk of bias for multiple domains of the Cochrane Risk of Bias tool.

Only two studies (Bittman, 2009; Tyson, 2002) described a random process of allocation, of which one (Tyson, 2002) reported a clear method of randomisation and was rated at low risk of bias on this domain. The remaining studies were at unclear risk (Bittman, 2009), or at high risk as they were not randomised (Anderson, 2010; Caulfield, 2022; DeCarlo, 2004). Of the randomised studies neither reported a process for ensuring concealed allocation and were judged at high risk of bias on this domain.

None of the studies attempted to blind participants or practitioners and all were rated at high risk of bias for this domain. Two studies (Anderson, 2010; Bittman, 2009) clearly did not ensure blinded assessors and were rated at high risk, and three (Caulfield, 2022; DeCarlo, 2004; Tyson, 2002) did not clearly report blinding of assessors and were rated at unclear risk. All studies were rated at high risk of bias for incomplete outcome data due to attrition levels and a lack of accounting for attrition in the analyses. All 5 studies were rated at unclear risk of bias for selective outcome reporting due to the lack of registration records of published protocols.

The single cross‐over trial (Bittman, 2009) was rated at high risk of bias for carry‐over effects as no washout period was observed, no baseline adjustment was applied in the analysis and there were signs of potential baseline imbalance on one outcome.

#### 5.2.2 Qualitative studies – Quality appraisal

See Table 3 for CASP quality appraisal of individual qualitative studies and Table 4 for CERQual evidence profile for qualitative studies.

**Table 3:** CASP quality checklist scores for qualitative studies.

References		Is the research design appropriate for addressing the aims of the research?	Was the recruitment strategy appropriate to the aims of the research?	Was the data collected in a way that addressed the research issue?	Has the relationship between researcher and participants been adequately considered?	Have ethical issues been taken into consideration?	Was the data analysis sufficiently rigorous?	Is there a clear statement of findings?	Contribution of the research on youth arts and offending behaviour	Total score Max = 8
1. Anderson (2010) MM		CT	Y	Y	N	N	N	Y	Y	4
2. Atherton (2022)		Y	Y	Y	CT	CT	Y	Y	Y	6
3. Baker (2007)		Y	CT	CT	N	N	CT	Y	Y	3
4. Barrett (2012)		Y	Y	Y	N	Y	N	Y	Y	6
5. Barrett (2015)		Y	Y	Y	N	Y	Y	Y	Y	7
6. Bowey (2006)		Y	Y	Y	N	Y	N	N	N	4
7. Caulfield (2019)		Y	Y	Y	CT	Y	CT	Y	Y	6
8. Caulfield (2022) MM		Y	Y	Y	Y	CT	Y	Y	N	6
9. Cesar (2020)		Y	Y	Y	CT	Y	Y	Y	N	6
10. Chong (2020)		Y	Y	Y	Y	Y	Y	Y	N	7
11. Clennon (2015)		Y	Y	Y	Y	CT	CT	Y	Y	6
12. Daykin (2017)		Y	Y	Y	CT	Y	Y	Y	Y	7
13. De Roeper (2009)		Y	CT	Y	N	N	CT	Y	Y	3
14. Flores (2016)		Y	Y	Y	Y	Y	CT	Y	N	6
15. Fullchange (2018)		Y	Y	Y	N	N	N	Y	Y	5
16. Gann (2010) GL MM		Y	Y	Y	N	Y	N	N	CT	4
17. Gowland‐Pryde (2016) GL		Y	Y	Y	Y	Y	Y	Y	Y	8
18. Hadland (2010)		Y	CT	Y	N	Y	CT	Y	N	4
19. Hanrahan (2017)		Y	Y	Y	Y	Y	Y	Y	Y	8
20. Hickey (2018)		Y	CT	CT	N	N	Y	Y	Y	4
21. Howard (2019/2022)		Y	Y	Y	Y	Y	Y	Y	Y	8
22. Jordan (2015)		Y	Y	CT	Y	Y	N	Y	N	5
23. Lazzari (2005)		Y	Y	Y	Y	CT	Y	Y	Y	7
24. Lea (2019)		Y	Y	Y	Y	Y	Y	Y	Y	8
25. Lotter (2005)		Y	CT	CT	CT	Y	Y	CT	CT	3
26. Massó‐Guijarro (2020)		Y	CT	CT	N	N	N	Y	Y	3
27. Morgan (2020)		Y	CT	Y	N	N	Y	Y	N	4
28. Nicklin (2017)		Y	Y	CT	N	CT	Y	Y	Y	5
29. Parker (2018)		Y	Y	Y	N	N	Y	Y	Y	6
30. Podkalicka (2009)		Y	CT	Y	N	N	CT	Y	Y	4
31. Pope (2022)		Y	CT	Y	N	Y	CT	Y	Y	5
32. Ruggiero (2013)		Y	CT	Y	N	N	Y	Y	Y	5
33. Seroczynski (2011)		C	Y	Y	N	N	CT	Y	Y	4
34. Tett (2012)		Y	Y	Y	N	N	CT	Y	Y	5
35. Thompson (2015)		Y	CT	Y	Y	N	CT	Y	Y	5
36. Thompson (2022)		Y	Y	Y	Y	Y	CT	Y	Y	7
37. Varley (2019) PhD, GL		Y	Y	Y	Y	Y	Y	Y	Y	8
38. Winn (2010)		Y	CT	CT	N	N	CT	Y	Y	3
39. Winn (2011)		Y	CT	CT	N	N	CT	Y	Y	3
40. Zlotowitz (2016)		Y	Y	Y	Y	Y	Y	Y	Y	8

*Note*: Y, yes; N, no; CT, can't tell.

**Table 4:** CERQual qualitative evidence profile.

Review findings (intervention processes/mechanisms)	Studies contributing to the review finding	Methodological limitations component	Relevance component	Coherence component	Adequacy of data component	Overall CERQual assessment of confidence	Explanation of judgement
Micro level influences on experiences, barriers and facilitators for arts‐based interventions for at risk and offending CYP (population level complexity)	1,2,3,4,5,6,7,8,9 10,11,12,13, 14,15,16,17, 18,19,20,21,23, 25,26,27,28,29,30,31,33,34,35,36,37,38,39,40 *N* = 37	Major concerns about methodological limitations (18 studies several limitations, 13 studies moderate limitations, 6 studies maximum quality)	Moderate concerns about relevance (all studies examined arts‐based intervention for at risk or offending young people to some extent although some with limited scope in recruitment strategies, very limited numbers and a lack of rigour in analysis)	Major concerns about coherence (data type reasonably consistent within studies, low consistency across studies on activity type, activity delivery and context, findings not always well grounded in the data or convincing about the impact of arts‐based interventions on at risk and offending young people)	Major concerns about adequacy (25 studies thin data, 6 studies moderate richness of data)	**Very Low Confidence**	Graded as very low confidence due to major concerns with methodological limits, coherence and adequacy and moderate concerns about relevance.
Meso level processes influencing design and delivery of arts‐based interventions for at risk and offending CYP (contextual, intervention and implementation complexity)	1,2,3,6,7,11,13,17,20,21,22,23,24,25,26,27,29,30,31, 32,34, 35,37 *N* = 23	Major concerns about methodological limitations (14 studies several limitations, 4 studies moderate limitations, 5 studies maximum quality)	Moderate concerns about relevance (all studies examined arts‐based intervention for at risk or offending young people to some extent although some with limited scope in recruitment strategies, very limited numbers and a lack of rigour in analysis)	Major concerns about coherence (data type reasonably consistent within studies, low consistency across studies on activity type, activity delivery and context, findings not always well grounded in the data or convincing about the impact or arts‐based interventions on at risk and offending young people)	Major concerns about adequacy (17 studies thin data, 1 study moderate richness of data)	**Very Low Confidence**	Graded as very low confidence due to major concerns with methodological limits, coherence and adequacy and moderate concerns about relevance.
Macro level influences on experiences, barriers and facilitators for arts‐based interventions for at risk and offending CYP	7,9,10, 38 *N* = 4	Major concerns about methodological limitations (1 study several limitations, 3 studies moderate limitations, 0 studies maximum quality)	Moderate concerns about relevance (all studies examined arts‐based intervention for at risk or offending young people to some extent although some with limited scope in recruitment strategies, very limited numbers and a lack of rigour in analysis)	Major concerns about coherence (data type reasonably consistent within studies, low consistency across studies on activity type, activity delivery and context, findings not always well grounded in the data or convincing about the impact or arts‐based interventions on at risk and offending young people)	Major concerns about adequacy (3 studies thin data)	**Very Low Confidence**	Graded as very low confidence due to major concerns with methodological limits, coherence and adequacy and moderate concerns about relevance.

*Note*: Numbers for studies contributing to the review refer to numbered studies in Table 3 CASP quality checklist scores for qualitative studies.

For the qualitative studies, the most frequent methodological weaknesses within the studies were limited discussion of recruitment strategies, missing detail about the process of data collection, a lack of rigour in data analysis, inadequate discussion of relationships between participants and researcher and incomplete information regarding ethical procedures, approvals or issues. The results of the quality checklist for qualitative studies varied with the best scoring (meeting 8 out of 8 criteria) in six sources (Gowland‐Pryde, 2016; Hanrahan, 2017; Howard, 2022; Lea, 2019; Varley, 2019; Zlotowitz, 2016) and the worst scoring (meeting 3 out of 8 criteria) in six sources (Baker, 2007; de Roeper, 2009; Lotter, 2015; Massó‐Guijarro, 2020; Winn, 2010, 2011).

The use of the CERQual schema for judging the confidence in the findings from the synthesis of qualitative evidence resulted in a judgement of very low confidence about the evidence for understanding micro, meso and macro‐level processes influencing the successful design and delivery of arts‐based interventions for at‐risk and offending young people, and their impact on behavioural, psychosocial, cognitive and offending outcomes. Very low confidence judgements are due to major concerns with methodological limitations, coherence and adequacy, and moderate concerns about relevance.

### 5.3 Effects of interventions

There was no adequate quantitative data to allow for our planned meta‐analyses, subgroup or sensitivity analyses, or to draw the pre‐planned contrasts between study characteristics described in our plan for narrative synthesis for any comparison.

#### 5.3.1 Arts interventions versus no intervention or usual care

We requested additional data to allow the analysis of effect sizes from Anderson (2010); Bittman (2009); Caulfield (2022); DeCarlo (2004) but did not receive these data. Results for the primary outcomes for this comparison are presented in Summary of findings Table 1.

##### Offending behaviour

No studies reported results for the outcome ‘Offending Behaviour’.

##### Anti or prosocial behaviour

One study Anderson (2010) (*n* = 30) reported the number of behavioural incidents recorded in the Young Offenders Institution. Results were reported for the 14 participants who completed the intervention only (4 in the music group, 5 in the sculpture group and 5 in the control group). During the intervention period, there were 7 incidents in the music group, 6 in the sculpture group and 4 in the control group. In the 3‐month post‐intervention period there were 3 incidents in the music group, 4 in the sculpture group and 5 in the control group. Data were not reported in a complete enough format to allow computation of effect sizes.

Tyson (2002) reported effects on peer relations using the 0–100 Index of peer relations (higher scores = worse peer relations). Post‐intervention there was no clear evidence for an effect of the arts intervention MD −3.53, 95%CI (1 −8.72 to 1.66, Analysis 1.1).

The evidence for both comparisons was rated as very low certainty, downgraded twice due to serious limitations and once due to imprecision.

Caulfield (2022) measured attitude and behaviour using the Youth Music Attitude and Behaviour scale but did not report specific numeric data to allow analysis of effect sizes.

##### Participation/attendance at arts intervention

Two studies (Anderson, 2010, Caulfield, 2022) reported attendance at the intervention.

Anderson (2010) (*n* = 30) reported attendance for the 14 participants who completed the intervention period. During the project period, 3 men attended 7 classes in the music group, 5 men attended 15 classes in the sculpture group and 5 men attended 12 classes in the control group. In the 3‐month period after the formal project intervention finished 3 men attended 12 classes in the music group, 4 men attended 13 classes in the sculpture group and 3 men attended 7 classes in the control group. Data were not reported in adequate detail to allow computation of effect sizes.

Caulfield (2022) (*n* = 187) measured attendance but did not report specific numeric data to allow the analysis of effect sizes.

The evidence for both comparisons was rated as very low certainty, downgraded twice due to serious limitations and once due to imprecision.

##### Psychological and emotional wellbeing

Four studies investigated a variety of measures of psychological and emotional well‐being (Anderson, 2010; Bittman, 2009; Caulfield, 2022; Tyson, 2002) of which two studies (Anderson, 2010; Tyson, 2002) reported adequate outcomes to allow the estimation of effect sizes. See Analysis 1.2.

###### Self‐Esteem

At post‐intervention, Anderson (2010) reported no clear evidence for an effect of a music intervention on self‐esteem, measured using the Rosenberg Self‐Esteem scale (*n* for comparison = 9, MD 0.32, 95% CI −0.39 to 1.03) or a sculpture intervention (*n* for comparison = 10, MD 0.50, 95% CI −0.21 to 1.21). The evidence was rated as very low certainty, downgraded twice due to serious limitations and once due to imprecision.

###### Locus of control

At post‐intervention, Anderson (2010) reported no clear evidence for an effect of a music intervention on locus of control, measured using the Locus of control behaviour scale (*n* for comparison = 9, MD −0.26, 95% CI −1.37 to 0.85) or a sculpture intervention (*n* for comparison = 10, MD −0.16, 95% CI −1.05 to 0.73). The evidence was rated as very low certainty, downgraded twice due to serious limitations and once due to imprecision.

###### Self‐concept

Tyson (2002) reported no clear evidence for an effect of Hip Hop Therapy post‐intervention on self‐concept, measured using the self‐concept scale for children (*n* for comparison = 11, MD 2.73, 95% CI −6.29 to 11.75, Analysis 1.2). The evidence for this comparison was rated as very low certainty, downgraded twice due to serious limitations and once due to imprecision.

Bittman (2009) measured psychopathology, anger, and depression but was a cross‐over trial. First‐phase data were not available in the study report or made available upon request. Caulfield (2022) measured well‐being but did not report data in a format that allowed the calculation of effect sizes.

##### Costs and associated economic outcomes

No studies reported results for this outcome.

##### Workplace engagement

No studies reported results for the outcome.

##### Adverse events

No studies reported results for the outcome.

#### 5.3.2 Arts interventions versus other types of arts intervention

Results for the primary outcomes for this comparison are presented in Summary of findings Table 2.

The only outcome where there was evidence comparing one arts intervention to another was psychological and emotional well‐being.

##### Psychological and emotional well‐being

Anderson (2010) (*n* = 30) compared a group receiving a music‐based arts intervention with a group receiving a sculpture‐based intervention. They reported results for this comparison based on 9 participants who completed the interventions.

Post‐intervention there was no clear evidence for an effect on self‐esteem (MD −0.18, 95% CI −0.76 to 0.4) or locus of control (MD −0.10, 95% CI −1.01 to 0.81, Analysis 2.1). The evidence was rated as very low certainty, downgraded twice due to serious limitations and once due to imprecision.

#### 5.3.3 Arts interventions versus non‐arts intervention

Only one study compared an arts intervention to a non‐arts intervention. DeCarlo (2004) compared RAP therapy to group psycho‐educational therapy using the composite outcome, the RAP Therapy assessment scale. However, data were not reported in adequate detail to allow the analysis of effect sizes.

### 5.4 Synthesis of qualitative evidence

Qualitative studies exploring the experiences and perceptions of participants, offered insight into the barriers and facilitators associated with delivering and receiving arts interventions or provided findings about aspects of the process of intervention delivery from the perspectives of those delivering and participants in the intervention and/or their carers/family members or significant agents (e.g., probation officers).

We took a broadly thematic approach to analysing and synthesising data from qualitative studies including a line‐by‐line reading for extraction, preliminary coding by two independent reviewers (L. M. and N. D.), dual development of descriptive themes and refinement of analytical themes by two reviewers (L. M. and N. D.) (Thomas, 2008). As planned, we conducted our thematic analysis with attention to the complexity of arts interventions for CYP at‐risk or in the criminal justice system. We mapped preliminary themes from the findings of the qualitative studies to theoretical domains of complexity relating to the intervention itself, the population, the implementation of the intervention, and the specific context that may impact on the process of delivering and engaging with the interventions. Further review and reflection by two authors (L. M./N. D.)identified that the components of complexity mapped on three overarching sets of processes; micro‐level, meso‐level and macro‐level. We therefore synthesised the findings further into micro, meso and macro level processes by which the experience of taking part in arts interventions impacted on behavioural, psychosocial, cognitive and offending outcomes for at‐risk and offending CYP. We report where such processes help to explain potential barriers and facilitators associated with delivering arts interventions for at risk and offending CYP.

#### 5.4.1 Micro‐level experiences, barriers and facilitators

Micro‐level processes refer to findings reported about individual participants in their setting and reflect a population complexity domain by identifying the relationship between CYP at‐risk of offending or who have already offended, arts interventions, individual demographics, cultures, sociopsychological and cognitive factors.

The qualitative studies all reported that participants experienced a range of positive emotions through taking part in arts‐based interventions. Some specific emotions were identified including feelings of hope and aspiration (Atherton, 2022), enjoyment (Gowland‐Pryde, 2016), self‐confidence or courage (Barrett, 2015; Caulfield, 2019; Gann, 2010; Lotter, 2015), pride in successfully creating an art form or gratitude at having access to art‐based interventions (Daykin, 2017; Hadland, 2010; Seroczynski, 2011), and being valued, respected and/or praised for their involvement and creative outcomes (Bowey, 2006; Caulfield, 2022: Chong, 2020; Lazzari, 2005; Nicklin, 2017; Parker, 2018; Thompson, 2015, 2022). Positive emotional experiences of arts‐based interventions generally underpinned and served to foster a sense of possibility or hope for the future, including able to manage and change more negative emotions and traumatic experiences associated with being at risk, having offended and/or being in the justice system (Caulfield, 2019; Fullchange, 2018; Hanrahan, 2017; Pope, 2022; Varley, 2019; Winn, 2010, 2011). Positive emotional experiences were also reported as encouraging development of a more positive attitude to learning, employment and skill development (Cesar, 2020; Tett, 2012) and acceptance of the potential benefits of creative activity (Caulfield, 2019).

In some studies, the elicitation of positive emotions was reported as enhancing self‐reflection and a critical self‐awareness for CYP defined as at‐risk or offending. Participants were reported as being able to redefine a more (self) caring and accepting sense of identity that challenged and resisted established and negative mindsets through learning new skills and achieving successful arts‐related outcomes (Atherton, 2022; Clennon, 2015; Gann, 2010; Hanrahan, 2017; Massó‐Guijarro, 2020; Morgan, 2020; Nicklin, 2017; Parker, 2018; Tett, 2012; Thompson, 2015; Winn, 2010, 2011). It was suggested by study authors that such processes of self‐determination could enable experiences of autonomy and empowerment through participation in art activities (Hickey, 2018; Lotter, 2015; Pope, 2022). The creative process itself was cited in some studies as important in allowing for experiences of self‐expression and sometimes through the perception that participation in the arts allows for exploration through risk in a safe context (Atherton, 2022; Baker, 2007; Barrett, 2012; Cesar, 2020; de Roeper, 2009; Gowland‐Pryde, 2016; Hickey, 2018; Lazzari, 2005; Lotter, 2015; Podkalicka, 2009; Varley, 2019).

Studies reported that arts‐based interventions for CYP at‐risk of offending or who have offended, contributed to the development of positive personal relationships with other CYP and adults involved in the arts intervention work. Arts‐based interventions were reported to allow for supportive interactions that could lead to relationships of trust, reciprocity and care (Atherton, 2022; Caulfield, 2019; Cesar, 2020; Daykin, 2017; Hanrahan, 2017; Lazzari, 2005; Lea, 2019; Massó‐Guijarro, 2020; Morgan, 2020; Nicklin, 2017; Parker, 2018; Zlotowitz, 2016). Arts interventions were also described as fostering more collaborative relationships based on sharing and/or listening, features that can support help‐seeking behaviours. Arts‐based interventions for CYP in this population groups were reported as helping participants to develop a sense of belonging and positive awareness of others including those in the justice setting and more broadly with families and communities.

Micro‐level barriers to the successful implementation of arts interventions for CYP at‐risk of offending or who have offended were identified to some extent. Where participants felt that they had no choice about participation or the arts activities and where arts interventions were not culturally relevant to them, implementation was challenging (Howard, 2022). Achieving positive outcomes from arts interventions was made difficult in situations where there were high levels of anxiety amongst participants generally (Flores, 2016) and where anxiety levels could be exacerbated in the context of having to learn something or perform (Fullchange, 2018; Hickey, 2018) There is also recognition that positive personal experiences happen only in the moment of taking part in arts and may not necessarily spill over into wider life (Cesar, 2020).

#### 5.4.2 Meso‐level processes influencing design and delivery

Meso‐level processes refer to findings reported in the qualitative studies about community‐level experiences and impacts as well as design and implementation of arts intervention for CYP at‐risk of offending or who have offended. In the qualitative studies meso level findings also capture connections between micro and meso‐level processes and reflect three complexity domains; contextual (residential/setting status and involvement, family and community support), intervention (providers, type, youth‐focused, facilities and theoretical framing) and implementation (mode delivery, fidelity, adherence, local support structures).

Qualitative studies identified the significance of understanding the setting in which arts interventions were taking place for successful engagement and delivery and provided evidence of the contextual complexity of arts intervention work. Studies reported that successful arts interventions in prison settings were designed to recognise but disrupt or distract from the punitive/penal structures (Atherton, 2022; Baker, 2007; Hickey, 2018; Lazzari, 2005). Arts‐based interventions were also reported as offering a more flexible environment than that of formal education, allowing participants to be creative, learn, achieve and interact with others in positive, relatively informal ways. The suggestion that this led to supportive and trusting relationships was made for interventions both in custodial and community (Caulfield, 2022; Morgan, 2020; Podkalicka, 2009) and educational settings (Jordan, 2015; Parker, 2018). Studies identified further contextual complexity in terms of the importance of ensuring that arts‐based interventions included culturally relevant programmes. Culturally relevant features included art activities that reflected the interests and needs of CYP at‐risk or offending and were meaningful to them such as music technology (Clennon, 2015), writing, poetry and music (Lea, 2019), songwriting, lyric writing and video recording (Massó‐Guijarro, 2020) rap music composition (Baker, 2007; Hickey, 2018) and music making (Thompson, 2015). Studies also reported elements of implementation complexity as important in ensuring arts‐based interventions elicited positive emotional and behavioural outcomes including enabling creativity as self‐expression and/or risk‐taking in a non‐judgemental (safe) environment (Atherton, 2022; Baker, 2007; Barrett, 2012; Cesar, 2020; de Roeper, 2009; Gowland‐Pryde, 2016; Hickey, 2018; Lotter, 2015; Podkalicka, 2009; Varley, 2019) and ensuring participants felt a sense of ownership and belonging in the arts intervention space (Bowey, 2006; Clennon, 2015; de Roeper, 2009; Pope, 2022). Intervention complexity was reported in studies including the importance of facilitator characteristics, attitudes and behaviours, expert arts instructions, leadership or facilitation, by a relatable and/or experienced ‘artist’ who was able to work the CYPs to co‐produce art, take a position that respectfully challenging young people to be creative and supported them in their endeavours to engage in the process of producing art (Atherton, 2022; Caulfield, 2019; Clennon, 2015; Daykin, 2017; Gowland‐Pryde, 2016; Howard, 2022; Lazzari, 2005; Pope, 2022; Tett, 2012). Additionally, complexity in barriers to design and implementation were reported to include negative attitudes of prison staff to arts interventions (Daykin, 2017; Howard, 2022).

#### 5.4.3 Macro‐level influences on experience, barriers and facilitators

Macro‐level processes refer to findings reported about wider societal, economic and political drivers and impacts of arts interventions. Macro‐level findings capture the interconnections between societal contexts in which arts interventions are designed and implemented and local experience of participants. Studies did not always refer directly to macro‐level processes in reports of findings, but there was some, albeit limited commentary on the ways in which these shaped complexity in terms of experiences of CYP at‐risk of offending or who had offended, the design and delivery of arts intervention, and their potential to impact on behavioural, psychosocial, cognitive and offending outcomes. We note here that the evidence for macro level process was extremely limited and restricted to four studies. Therefore, clear conclusions about them are not possible. One qualitative study from the USA reported the arts intervention as a response to a lack of policy advocacy for the arts for CYP at risk of offending or who had offended and associated limits to funding streams which impact negatively on prioritising of arts programmes in the sector (Cesar, 2020). One study highlighted the need to develop interventions with external partners in policy and practice to implement sustainable and successful arts programmes for offending CYP connected to the justice system (Caulfield, 2019). One study from South Korea (Chong, 2020) and another from the USA (Winn, 2010) identified the need to connect arts‐based interventions to wider communities and national organisations for supporting CYP at‐risk of offending CYP. The study implied that partnership working between the justice system, and education, housing and healthcare organisations could serve to support more effective and sustainable design and delivery of arts programmes to address potential and actual offending behaviours.

## 6 DISCUSSION

### 6.1 Summary of main results

#### 6.1.1 Quantitative evidence

We found insufficient evidence from quantitative studies to support or refute the effectiveness of arts interventions for CYP at‐risk of offending or who have offended for any outcome. From the included studies there was only very low certainty evidence and no clear evidence of an effect of arts interventions for any of the included outcomes. We found no evidence for our primary outcome ‘Offending Behaviour’. The evidence base is poorly developed and there is no published evidence available.

#### 6.1.2 Qualitative

The qualitative studies in this review showed that micro‐level experiences of arts‐based interventions for CYP at‐risk of offending or who had offended which may influence the success of arts‐based interventions broadly reflected individual positive personal experiences. These included positive emotions, the development of a sense of self, successful engagement in creative processes and practices, and the development of positive personal relationships with people involved in the intervention (other participants, prison staff and delivery experts) and other people in their lives, for example, family, friends and communities. Meso‐level contextual processes influence the success of arts‐based interventions and include the need for accessible delivery sites which can offer flexibility in timing and access to, and support for using appropriate space and facilities in which arts‐based interventions can act as a creative alternative to restricted life in youth justice settings. Qualitative studies reported the need for arts‐based interventions to be designed as culturally relevant (reflecting race/ethnic, national identify, gender and age characteristics, for example), youth‐focused and theoretically framed with models or theories relevant to practice, consistent, regular and sustainable if they are to be successful in engaging CYP at‐risk of offending or those who have offended. In addition, the role of supportive staff, family and community members, and expert delivery by professional artists to whom participants could relate were also reported as meso‐level intervention processes important to the successful implementation of arts programmes for this population. Macro‐level barriers to the implementation of arts interventions in youth justice settings were reported in very few studies but lack of policy advocacy for arts in youth justice settings is highly likely to mean that funding is limited and that arts interventions are not prioritised. There was reference in one study to the need to connect arts interventions to wider support in relation to education, housing, and healthcare. This is because it is likely that the most successful and sustainable arts interventions for CYP at‐risk of offending or who have offended will be informed and supported by key personnel in schools, communities and healthcare.

### 6.2 Overall completeness and applicability of evidence

The review was based on a broad and inclusive search strategy agreed with the Advisory Board to ensure completeness and applicability of evidence. Most studies focused on music interventions with few examining a wider range of arts‐based practices. All studies included young people and a broad age range (7–25 years) providing evidence applicable to CYP at‐risk of offending or who have offending.

Quantitative studies were generally small, single‐centre studies with only short‐term follow‐up and were all conducted in the USA or the UK. Of these one was conducted in a school and the others were all conducted in young offenders or residential youth facilities. There was limited evidence available to inform conclusions for any outcome or to draw comparisons between different study characteristics.

Qualitative studies tended to focus on the experiences of individual participants with fewer explaining the processes by which interventions were successfully designed and implemented for offending and behavioural outcomes. Detail on participant demographics was limited beyond reports of sex/gender and simple descriptions of ethnicity. There was no detailed analysis of the impact of racial/ethnic characteristics on the success of arts interventions beyond generalised commentary on ensuring interventions were culturally relevant, that is, reflected race/ethnic, national identity, gender and age characteristics of the group.

### 6.3 Quality of the evidence

#### 6.3.1 Quantitative

All studies were small, conducted in a single centre, and at high risk of bias across multiple domains. Only two studies were described as randomised and, of those, only one described that process adequately. No study reported a process of allocation concealment. While blinding is clearly challenging in this field there was little detail reported on attrition or appropriate methods of analysis to account for it. No study was pre‐registered with an available protocol, and so there is a risk of selective outcome reporting and other post hoc changes in study conduct. The reported details of interventions was often quite superficial, creating challenges for understanding what was done, and subsequently for replication.

#### 6.3.2 Qualitative

Methodological weaknesses were found in most qualitative studies including limited discussion of recruitment strategies, scant detail about data collection, a lack of rigour in data analysis, no adequate discussion of relationships between participants and researcher, and a lack of information regarding ethical procedures, approvals or issues. The use of the CERQual schema for judging the confidence in the findings from the synthesis of qualitative evidence results in a judgement of very low confidence about the evidence for understanding micro, meso, and macro‐level processes influencing the successful design and delivery of arts‐based interventions for at‐risk and offending young people and their impact on behavioural, psychological, cognitive and offending outcomes. Very low confidence judgements are due to major concerns with methodological limitations, coherence and adequacy, and moderate concerns about relevance.

### 6.4 Potential biases in the review process

Potential biases in the review were mitigated through peer review and the publication of a review protocol before the commencement of the review. The review process included rigorous test searching and refinement of search strategies to reflect variability in database functionality in consultation with stakeholders. Non‐English studies were included. Grey literature sources were included as a way to reduce publication bias in the review and ensure a timely inclusion strategy. Our systematic search strategy ensured that this review represents a comprehensive summary of all existing eligible studies. We found that the CASP checklist for qualitative research could be interpreted as somewhat forgiving on quality appraisal items and may result in potential reporting bias of very low‐quality qualitative studies in this review.

We initially planned to only include first‐phase data from cross‐over studies due to the risk of carry‐over effects with this design. However, to avoid excluding what little evidence was available we included a crossover design and reported its findings. This allows for a complete description of the available evidence, and as we were not able to pool data, does not introduce an unaddressed bias or unit of analysis issue to any specific analyses.

### 6.5 Agreements and disagreements with other studies or reviews

A very small number of previous reviews have examined arts and young people in justice settings, although few have included qualitative research. Here we compare findings from a review of music in youth justice settings (Daykin, 2013) and a review of arts with offender populations more widely (Meekums, 2011). Consistent with this review, music is the most commonly reported intervention. There is some overlap in terms of reporting of experiences, barriers, and facilitators that map onto our micro, meso and macro framework. For instance, in their review of four studies Meekums (2011) report that arts can have a positive effect on mental health and can enhance emotional literacy, themes that reflect changes at the micro level of personal well‐being and development. From five qualitative studies reviewed by Daykin (2013) themes most strongly reflect micro and meso domains. These include personal processes of identity formation, expression, and empowerment as well as programme features including music genre and cultural relevance. The macro domain is reflected in the theme of sustainability and resources. Daykin (2013) identify potential barriers as well as facilitators including accessibility, organisational fit, short‐term planning, lack of resources and policy environments that constrain provision.

Both previous reviews recognise limitations in the application of qualitative methodologies that make it difficult to assess the credibility of research findings. Daykin (2013) identify specific limitations including poorly described interventions, lack of detailed reporting of sampling, recruitment and data collection, and weak or poorly described processes of analysis that often rely on face‐value reporting of programme benefits in language that is suggestive of outcomes rather than processes, which is not suited to qualitative research.

There are no major points of disagreement with previous qualitative reviews. What is notable is how little the field has moved on in the 10 or more years since these reviews were published, both in terms of the volume of studies produced and the methodological quality. This may be reflective of the wider context and policy environment, compounded by the COVID‐19 pandemic, which has seen a reduction in arts‐based provision and research in youth justice settings.

## 7 AUTHORS' CONCLUSIONS

### 7.1 Implications for practice and policy

The fact that the current evidence base is limited in quality and scope does not allow us to conclude that arts interventions work or do not work to address offending and behavioural outcomes for CYP at‐risk of offending behaviour or who have offended. However, there are some wider implications for practice and policy.

Some higher quality qualitative studies provided relatively rich data about the positive impact of arts‐based practice for this population including: pleasing emotional experiences, successful engagement in creative learning, development of positive personal relationships with others involved in the intervention and/or their families and communities, and sense of self. There is also some consensus in the qualitative evidence about the characteristics that seem to make arts practices acceptable to young people, such as cultural relevance (e.g., reflecting race/ethnicity, national identity, age and gender), and the importance of mediators including supportive staff with identified roles and appropriate workloads, and especially the attributes and practices of expert and professional artist facilitators. The qualitative data in this review point to some features of best practice even in the absence of outcomes evidence and the overall poor methodological quality in the studies. However, there is currently inadequate quantitative evidence to confirm or support the importance of these factors.

The policy and practice environment for arts interventions for CYP at‐risk of offending or those who have offended has not developed extensively since previous and earlier reviews on this topic. There is insufficient evidence and a dearth of high‐quality findings in this review on which to recommend which, if any, arts‐based interventions should be designed and implemented, in what ways, for which CYP at‐risk of offending or who have offended, and in what contexts. It should be noted that working with this population is extremely challenging in research terms due to the transient nature of their connection to the justice system and to a systemic lack of tracking individuals meaning long‐term follow‐up is often very difficult. However, this lack of development also suggests that there has been limited investment in research and development of arts‐based approaches to working with CYP in the justice sector in both custodial and community settings. This in turn could reflect wider shifts in arts, educational and penal policy, including a shift away from rehabilitation‐based approaches as well as a decline in arts education more generally. It is likely that funding is limited for arts interventions in youth justice contexts. The COVID‐19 pandemic will have exacerbated the lack of policy and practice advocacy and there is no evidence for post‐pandemic recovery.

### 7.2 Implications for research

Youth justice environments are complex settings in which there are many barriers to research including small‐scale interventions, transient populations, unpredictable environments and difficulties in tracking study participants in the longer term. This review found that practice in the field of arts and youth justice has not developed at any scale that would allow for rigorous research and evaluation.

For any future research to be successful there will be a need to carefully consider the multiple sources of complexity including those of the setting, the population, the culture and agents at play in the setting, the intervention and the outcomes. There are three broad recommendations for research:

1. Co‐production of interventions with all stakeholders is vital identify and develop the most relevant arts interventions that meet the complex needs of at‐risk and offending young people
2. Rigorous and systematic intervention development needs to be founded upon clear theoretical underpinning, identified objectives and a sound process of feasibility and acceptability testing
3. Attention to quality principles across a range of methods including mixed method is needed to ensure: (i) adequately powered controlled studies, designed to minimise avoidable sources of bias, including the routine adoption of pre‐registration and peer‐reviewed protocols and data sharing, reporting of processes and outcomes to established standards of best practice, and formal post‐trial implementation studies to evaluate the success of interventions in wider practice, and (ii) robust process evaluations using established guidance.

For this research to succeed, substantial investment and both cross‐sector and interdisciplinary collaboration will be required.

## CONTRIBUTIONS OF AUTHORS

Please give brief description of content and methodological expertise within the review team. The recommended optimal review team composition includes at least one person on the review team who has content expertise, at least one person who has methodological expertise and at least one person who has statistical expertise. It is also recommended to have one person with information retrieval expertise.

Who is responsible for the below areas? Please list their names:

Content: Louise Mansfield, Norma Daykin and Louise Forde

Systematic review methods: Neil O Connell

Statistical analysis: Neil O Connell and Daniel Bailey

Information retrieval: Robyn Smith, Jake Gifford and Garcia Ashdown‐Franks

## DECLARATIONS OF INTEREST

None

**Preliminary timeframe**

Approximate date for submission of the systematic review.

May 2023

**Plans for Updating this review**

Review will be updated if there is an identified need and funding available.

## DIFFERENCES BETWEEN PROTOCOL AND REVIEW

For cross‐over studies, we planned in our protocol to only include data from the first phase of the study, when they are available due to the risk of carryover effects. However, as first‐phase, or phase‐by‐phase data were not available for any of the included cross‐over studies we took the decision to extract effect sizes from these studies as they were reported.

## DATA AND ANALYSES

**1. Arts interventions versus no intervention/usual practice**

**Table:** 

Outcome or subgroup	Studies	Participants	Statistical method	Effect estimate
1.1 Antisocial or prosocial behaviour: peer relations [0–100]	1		Mean Difference (IV, Random, 95% CI [0–100])	No totals
1.2 Psychological and Emotional Wellbeing	2		Mean Difference (IV, Fixed, 95% CI)	No totals

**2 Arts intervention versus other Arts intervention**

**Table:** 

Outcome or subgroup	Studies	Participants	Statistical method	Effect estimate
2.1 Psychological and Emotional Wellbeing	1		Mean Difference (IV, Fixed, 95% CI)	No totals

## SOURCES OF SUPPORT

**Internal sources**

- Brunel University London Information Services, UK
  Support for database searching
**External sources**
- Youth Endowment Foundation, UK

Funding support and secretariat for Advisory Board

- Arts Council England, UK

Funding and Advisory Support

## SUMMARY OF FINDINGS

**Summary of findings tables**

Tables 1 and 2.

**Table 1:** Arts interventions versus no treatment/usual care.

Population: Children and young people (8–25 years) identified as at‐risk of offending behaviour or already in the criminal justice system. Intervention: interventions involving arts participation/Comparison: No intervention or usual care					
Outcomes	Probably outcome with the intervention/effect size	Probable outcome with no treatment/usual care	No. of participants (studies)	Certainty of the evidence (GRADE)	Comments
**Short‐term follow‐up (immediately post‐intervention to <3 months)**					
Offending behaviour	No data				
Anti‐social or pro‐social behaviours: No. of behavioural incidents	Music Intervention: 4 incidents Sculpture Intervention: 5 incidents	5 incidents	14 (1)	⊕⊝⊝⊝ very low^a^	
Anti‐social or pro‐social behaviours: behavioural incidents. Peer Relations (Index of Peer relations 0–100 scale, higher scores = worse peer relations)	Mean Difference (MD) −3.53, 95% CI (1 −8.72 to 1.66)	Post‐test score 40.33 (3.44)	11 (1)	⊕⊝⊝⊝ very low^a^	
**Medium‐term follow‐up (3 to <12 months post‐intervention)**					
Offending behaviour	No data				
Anti‐social or pro‐social behaviours	No data				
**Long‐term follow‐up (>1 year post‐intervention)**					
Offending behaviour	No data				
Anti‐social or pro‐social behaviours	No data				

^a^ Downgraded twice for serious study limitations and once for imprecision.

**Table 2:** Arts intervention versus other arts intervention.

Population: Children and young people (8–25 years) identified as at‐risk of offending behaviour or already in the criminal justice system. Intervention: interventions involving arts participation Comparison: other intewrvention involvoing arts participation					
Outcomes	Probably outcome with the intervention/effect size	Probable outcome with no treatment/usual care	No. of participants (studies)	Certainty of the evidence (GRADE)	Comments
**Short‐term follow‐up (immediately post‐intervention to <3 months)**					
Offending behaviour	No data				
Anti‐social or pro‐social behaviours	No data				
**Medium‐term follow‐up (3 to <12 months post‐intervention)**					
Offending behaviour	No data				
Anti‐social or pro‐social behaviours	No data				
**Long‐term follow‐up (>1 year post‐intervention)**					
Offending behaviour	No data				
Anti‐social or pro‐social behaviours	No data				

## Supporting information

Supporting information.

Supporting information.

## References

[cl21377-bib-0002] Anderson, K. , & Katie, O. (2010). Engaging Scottish young offenders in education through music and art. International Journal of Community Music, 3(1), 47–64.

[cl21377-bib-0003] Atherton, S. , Knight, V. , & van Barthold, B. C. (2022). Penal arts interventions and hope: Outcomes of arts‐based projects in prisons and community settings. The Prison Journal (Philadelphia, Pa.), 102(2), 217–236.

[cl21377-bib-0004] Baker, S. , & Homan, S. (2007). Rap, recidivism and the creative self: A popular music programme for young offenders in detention. Journal of Youth Studies, 10(4), 459–476.

[cl21377-bib-0005] Barrett, M. S. , & Baker, J. S. (2012). Developing learning identities in and through music: A case study of the outcomes of a music programme in an Australian juvenile detention centre. International Journal of Music Education, 30(3), 244–259.

[cl21377-bib-0006] Barrett, M. S. , & Nigel, B. (2015). Connecting through music: The contribution of a music programme to fostering positive youth development. Research Studies in Music Education, 37(1), 37–54.

[cl21377-bib-0007] Bittman, B. , Larry, D. , & Kim, C. (2009). Creative musical expression as a catalyst for quality‐of‐life improvement in inner‐city adolescents placed in a court‐referred residential treatment program. Advances in Mind Body Medicine, 24(1), 8–19. 20671333

[cl21377-bib-0008] Bowey, L. , & Alex, M. G. (2006). The youth crime reduction video project: An evaluation of a pilot intervention targeting young people at risk of crime and school exclusion. Howard Journal of Criminal Justice, 45(3), 268–283.

[cl21377-bib-0009] Caulfield, L. , Sojka, B. , & Massie, R. (2019). An evaluation of Sandwell Youth Offending Service – A creative approach to working with young people. Sandwell YOS – A creative approach to working with young people. University of Wolverhampton – Issue.

[cl21377-bib-0010] Caulfield, L. , Jolly, A. , Simpson, E. , & Devi‐McGleish, Y. (2022). ‘It's not just music, it helps you from inside’: Mixing methods to understand the impact of music on young people in contact with the criminal justice system. Youth Justice – An International Journal, 22(1), 67–84.

[cl21377-bib-0011] Cesar Gabriel, T. , & Decker Scott, H. (2020). ‘CPS sucks, but… I think I'm better off in the system’: Family, social support, & arts‐based mentorship in child protective services. Child and Youth Services Review, 118, 1–13.

[cl21377-bib-0012] Chong, H. J. , & Juri, Y. (2020). Music therapy for delinquency involved juveniles through tripartite collaboration: A mixed method study. Frontiers in Psychology, 11, 589431. 33192927 10.3389/fpsyg.2020.589431 PMC7645031

[cl21377-bib-0013] Clennon, O. D. (2015). ‘Holdin' on’: Using music technology as a tool of cultural liberation with respect to performing masculinities at a young offenders' institution. In Liberation practices (pp. 71–83). Routledge.

[cl21377-bib-0014] Daykin, N. , de Viggiani, N. , Yvonne, M. , & Paul, P. (2017). Music‐making for health and wellbeing in youth justice settings: Mediated affordances and the impact of context and social relations. Sociology of Health and Illness, 39(6), 941–958. 28332197 10.1111/1467-9566.12549

[cl21377-bib-0015] DeCarlo, A. , & Hockman, E. (2004). RAP therapy: A group work intervention method for urban adolescents. Social Work Groups, 26(3), 45–59.

[cl21377-bib-0016] de Roeper, J. , & Savelsberg Harry, J. (2009). Challenging the youth policy imperative: Engaging young people through the arts. Journal of Youth Studies, 12(2), 209–225.

[cl21377-bib-0017] Flores, K. , van Niekerk, C. , & le Roux, L. (2016). Drumming as a medium to promote emotional and social functioning of children in middle childhood in residential care. Music Education Research, 18(3), 254–268.

[cl21377-bib-0018] Fullchange, A. , & Sharkey, J. D. (2018). Experience and outcomes of a theatre intervention for youth on probation and their university peers. Appl Psychol Crim Justice, 14(1), 70–85.

[cl21377-bib-0019] Gann, E. (2010). *The effects of therapeutic hip hop activity groups on perception of self and social supports in at‐risk urban adolescents[PhD Dissertation]*, 71(5), 3355.

[cl21377-bib-0020] Gowland‐Pryde, R . (2016). Leaping forward: From ‘young offenders’ to ‘young artists’ (Doctoral dissertation, University of Southampton).

[cl21377-bib-0021] Hadland, R. , & Theodore, S. (2010). Community art project for excluded teenagers. Mental Health Practice, 13(6), 18–23.

[cl21377-bib-0022] Hanrahan, F. , & Robin, B. (2017). ‘It makes me feel alive’: The socio‐motivational impact of drama and theatre on marginalised young people. Emotional and Behavioural Difficulties, 22(1), 35–49.

[cl21377-bib-0023] Maud, H. (2018). ‘We all come together to learn about music’: A qualitative analysis of a 5‐year music program in a juvenile detention facility. International Journal of Offender Therapy and Comparative Criminology, 62(13), 4046–4066. 29562798 10.1177/0306624X18765367

[cl21377-bib-0024] Howard, F. (2022). Using and abusing the arts with ‘At‐Risk’ youth. Journal of Applied Youth Studies, 5(2), 101–116.

[cl21377-bib-0025] Howard, F. (2019). Artistic production and (re) production: Dis‐engaged young people's educational experiences of Arts Award programmes [PhD Dissertation], 1, 1–200. https://eprints.nottingham.ac.uk/55708/

[cl21377-bib-0026] Jordan, C. , & Jerry, D. (2015). Theatre‐in‐diversion: Evaluating an Arts‐Based approach to combating juvenile delinquency. Theatre Symposium, 23, 81–138.

[cl21377-bib-0027] Lazzari, M. M. , Amundson, K. A. , & Jackson, R. L. (2005). “We are more than jailbirds”: An arts program for incarcerated young women. AFfilia – Journal of Women and Social Work, 20(2), 169–185.

[cl21377-bib-0028] Lea, C. H. , Malorni, A. , & Jones, T. M. (2019). “Everybody is an artist”: Arts‐based education and formerly incarcerated young Black men's academic and social–emotional development in an alternative school. American Journal of Community Psychology, 64(3–4), 333–347. 31449678 10.1002/ajcp.12378

[cl21377-bib-0028a] Lotter, C. B. (2005). Circles of Courage: Music therapy with adolescents in conflict with the law at a community based setting (Doctoral dissertation, University of Pretoria).

[cl21377-bib-0029] Lotter, C. B . (2015). Circles of Courage: Music therapy with adolescents in conflict with the law at a community based setting [PhD Dissertation], 1, 1–450.

[cl21377-bib-0030] Massó‐Guijarro, B. , & Montes‐Rodríguez, R. (2020). Educational and social uses of music and video creation for a group of young offenders. International Journal of Interdisciplinary Cultural Studies, 16(1), 1–14.

[cl21377-bib-0031] Morgan, H. , Andrew, P. , & Naomi, M. (2020). Community‐based intervention and marginalised youth: Inclusion, social mobility and life‐course transition. Journal of Education and Work, 33(5–6), 327–342.

[cl21377-bib-0032] Nicklin, L. L. (2017). ‘Make not your prisons your prisons’: Participant‐percieved potential outcomes of a Shakespeare focussed alternative to juvenile incarceration in the USA. EMOTIONAL BEHAV DIFFICULTIES, 22(1), 2–17.

[cl21377-bib-0033] Parker, A. , Marturano, N. , O'Connor, G. , & Meek, R. (2018). Marginalised youth, criminal justice and performing arts: Young people's experiences of music‐making. Journal of Youth Studies, 21(8), 1061–1076.

[cl21377-bib-0034] Podkalicka, A. (2009). Young listening: An ethnography of YouthWorx media's radio project. Continuum – Journal of Media & Cultural Studies, 23(4), 561–572.

[cl21377-bib-0035] Pope, R. J. , & Jones, J. N. (2022). Creative and expressive arts in a young adult problem‐solving court and its influence on participant experience and outcomes. Youth & Society, 54(7), 1225–1246.

[cl21377-bib-0036] Dana, R. , de Hurtado Belen, G. , & Watson William, R. (2013). Juvenile offenders: Developing motivation, engagement, and meaning‐making through video game creation. International Journal of Game‐Based Learning, 3(2), 112–129.

[cl21377-bib-0037] Seroczynski, A. D. , Johnson Scott, P. , Lamb, K. , & Gustman, B. (2011). The hidden virtues of Harry Potter: Using J K Rowling's novels to facilitate character education with juvenile delinquents. Journal of Research in Character Education, 9(1), 1–24.

[cl21377-bib-0038] Tett, L. , Anderson, K. , Mcneill, F. , Overy, K. , & Sparks, R. (2012). Learning, rehabilitation and the arts in prisons: A Scottish case study. Studies in the Education of Adults, 44(2), 171–185.

[cl21377-bib-0039] Thompson, J. D. (2015). Towards cultural responsiveness in music instruction with Black detained youth: An analytic autoethnography. Music Education Research, 17(4), 421–436.

[cl21377-bib-0040] Thompson, B. U. (2022). Beyond the corner: Introducing a string music program to youth in detention. String Research Journal, 12(1), 61–83.

[cl21377-bib-0041] Tyson Edgar, H. (2002). Hip hop therapy: An exploratory study of a rap music intervention with at‐risk and delinquent youth. Journal of Poetry Therapy, 15(3), 131.

[cl21377-bib-0042] Varley, D. S (2019). Acting around in young offender rehabilitation: Investigating how psychological theory fused with drama techniques can create a model (The V² Model) for reducing crime when working with young offenders within the community [PhD Dissertation].

[cl21377-bib-0043] Winn, M. T. (2010). ‘Our side of the story’: Moving incarcerated youth voices from margins to center. Race Ethnicity and Education, 13(3), 313–325.

[cl21377-bib-0044] Winn, M. T. , & Jackson Chelsea, A. (2011). Toward a performance of possibilities: Resisting gendered (in) justice. International Journal of Qualitative Studies in Education, 24(5), 615–620.

[cl21377-bib-0045] Zlotowitz, S. , Chris, B. , Olive, M. , & Charlotte, H. (2016). Service users as the key to service change? The development of an innovative intervention for excluded young people. Child and Adolescent Mental Health, 21(2), 102–108. 32680374 10.1111/camh.12137

[cl21377-bib-0047] Ajayi, M. (2015). Feasibility of creative clinical intervention groups in reducing depression and anxiety in prisons…. In 39th annual conference and exhibition of the College of Occupational Therapists, Brighton and Sussex, England (p. 5).

[cl21377-bib-0048] Aleksienė, V. , & Ambrasaitė, R. (2014). Jaunimo kultūrų įvairovė: gatvės meno projektai kaip nusikalstamo elgesio prevencija. Jaunimo kultūrų įvairovė: gatvės meno projektai kaip nusikalstamo elgesio prevencija. Socialinis ugdymas, 2, 68–82.

[cl21377-bib-0049] Amitay, G. (2022). Capoeira clubs as inclusive and therapeutic communities for youth and young adults experiencing social exclusion. International Journal of Qualitative Studies in Education, 1, 1–18.

[cl21377-bib-0050] Argyle, E. , & Gillie, B. (2005). Art in the community for potentially vulnerable mental health groups. Health Education, 105(5), 340–354.

[cl21377-bib-0051] Arroyo, M. , Chiarini, C. , & Yamaoka, D. (2019). Music education and public policies for primary social protection and socio‐educational measures involving adolescents in the City of Sao Paulo. Opus, 1, 446–473.

[cl21377-bib-0052] Aventin, A. , French, R. , Young, H. , McDaid, L. , Lewis, R. , Warren, E. , McConnon, L. , & Lohan, M. (2019). Acceptability of an interactive film‐based intervention targeting adolescent boys to prevent sexual risk‐taking: Findings from the JACK cluster randomised controlled trial process evaluation. The Lancet, S5, 394.

[cl21377-bib-0053] Basto‐Pereira, M. , Rita, C. , Sofia, R. , & Ângela, M. (2015). Long‐term predictors of crime desistance in juvenile delinquents: A systematic review of longitudinal studies. Aggression and Violent Behavior, 25, 332–342.

[cl21377-bib-0054] Julie, B. , Marcela, O. , & Elizabeth, K. (2010). A community‐based hip‐hop dance program for youth in a disadvantaged community in Ottawa: Implementation findings. Health Promotion Practice, 11(3_Suppl.), 61S–69S. 20488970 10.1177/1524839909353738

[cl21377-bib-0055] Blau, I. , & Nurit, B. (2016). Can designing self‐representations through creative computing promote an incremental view of intelligence and enhance creativity among at‐risk youth? Interdisciplinary Journal of e‐skills and Lifelong Learning, 12, 267–278.

[cl21377-bib-0056] Bornmann Barbara, A. , & Crossman Angela, M. (2011). Playback theatre: Effects on students' views of aggression and empathy within a forensic context. The Arts in Psychotherapy, 38(3), 164–168.

[cl21377-bib-0057] Bramwell, R. (2018). Freedom within bars: Maximum security prisoners' negotiations of identity through rap. Identities, 25(4), 475–492.

[cl21377-bib-0058] Bravo, C. A. , & Urrutia, R. B. (2022). Mental health behind bars: Art as a therapeutic tool with young law offenders in Chile. CUHSO (Temuco), 32(1), 1–10.

[cl21377-bib-0059] Brooks, C. M. , Daschuk, M. D. , Poudrier, J. , & Almond, N. (2015). First nations youth redefine resilience: Listening to artistic productions of ‘Thug Life’ and hip‐hop. Journal of Youth Studies, 18(6), 706–725.

[cl21377-bib-0060] Bulgren Christopher, W. (2020). Jail guitar doors: A case study of guitar and songwriting instruction in Cook County jail. International Journal of Community Music, 13(3), 299–318.

[cl21377-bib-0061] Caló, F. , Artur, S. , Stephen, M. , & Simon, T. (2020). The impact of a community‐based music intervention on the health and well‐being of young people: A realist evaluation. Health & Social Care in the Community, 28(3), 988–997. 31876078 10.1111/hsc.12931 PMC7187212

[cl21377-bib-0062] Campbell, M. (2019). From youth engagement to creative industries incubators: Models of working with youth in community arts settings. Review of Education, Pedagogy, and Cultural Studies, 41(3), 164–192.

[cl21377-bib-0063] Carder Macaela, M. (2007). Book review: “Theatre in prison: Theory and practice”. Theatre Journal, 59(1), 155–156.

[cl21377-bib-0064] Carpenter, B. , & Victoria, K. (2018). Potential unlocked: Art in prison. Scene, 6(1), 7–12.

[cl21377-bib-0065] Cleaver, D. , & Stewart, R. (2014). Music as engaging, educational matrix: Exploring the case of marginalised students attending an “Alternative” music industry school. Research Studies in Music Education, 36(2), 245–256.

[cl21377-bib-0066] Cursley, J. , & Maruna, S. (2019). The music of recovery and desistance prison‐based musical tuition as a strengths‐based intervention (1, pp. 257–276). Routledge.

[cl21377-bib-0067] Dalgaard, N. T. , Bondebjerg, A. , Viinholt, B. C. A. , & Filges, T. (2022). The effects of inclusion on academic achievement, socioemotional development and wellbeing of children with special educational needs. Campbell Systematic Reviews, 18(4), e1291. 36908836 10.1002/cl2.1291 PMC9727566

[cl21377-bib-0068] Davis, S. K. (2020). Dancing in the street: Impacting at‐risk youths' lives through the arts. Sociological Perspectives, 63(3), 516–518.

[cl21377-bib-0069] Daykin, N. , de Viggiani, N. , Pilkington, P. , & Moriarty, Y. (2013). Music making for health, well‐being and behaviour change in youth justice settings: A systematic review. Health Promotion International, 1, 197–210. 10.1093/heapro/das005 22415559

[cl21377-bib-0070] Deuchar, R. , & Ellis, J. (2013). ‘It's helped me with my anger and I'm realising where I go in life’: The impact of a Scottish youth work/schools intervention on young people's responses to social strain and engagement with anti‐social behaviour and gang culture. Research in Post‐Compulsory Education, 18(1–2), 98–114.

[cl21377-bib-0071] Dodsley, T. , & Emily, G. (2021). Resistance and reproduction: An arts‐based investigation into young people's emotional responses to crime. The British Journal of Criminology, 61(2), 456–475.

[cl21377-bib-0072] Edmunds, K. , Ling, R. , Shakeshaft, A. , Doran, C. , & Searles, A. (2018). Systematic review of economic evaluations of interventions for high risk young people. BMC Health Services Research, 18(1), 1–10. 30139384 10.1186/s12913-018-3450-x PMC6108123

[cl21377-bib-0073] Evans‐Chase, M. , & Huiquan, Z. (2014). A systematic review of the juvenile justice intervention literature. Crime and delinquency, 60(3), 451–470.

[cl21377-bib-0074] Everett, C. , Chadwell, J. , & McChesney Jon, C. (2002). Successful programs for at‐risk youths. Journal of Physical Education, Recreation & Dance, 73(9), 38–43.

[cl21377-bib-0075] Gallagher, K. , & Rodricks Dirk, J. (2017). Performing to understand: Cultural wealth, precarity, and shelter‐dwelling youth. The Journal of Applied Theatre and Performance, 22(1), 7–21.

[cl21377-bib-0076] Geagea, A. , Lynette, V. , & Judith, M. C. (2019). Creative arts outreach initiatives in schools: Effects on university expectations and discussions about university with important socialisers. Higher Education Research and Development, 38(2), 250–265.

[cl21377-bib-0077] Harding, C. G. , Safer, L. A. , Kavanagh, J. , Bania, R. , Carty, H. , Lisnov, L. , & Wysockey, K. (1996). Using live theatre combined with role playing and discussion to examine what at‐risk adolescents think about substance abuse, its consequences, and prevention. Adolescence, 31(124), 783. 8970653

[cl21377-bib-0078] Harkins, L. , Cecilia, P. , Donna, H. , Andy, W. , & Beech Anthony, R. (2011). Evaluation of geese theatre's re‐connect program: Addressing resettlement issues in prison. International Journal of Offender Therapy and Comparative Criminology, 55(4), 546–566. 20472705 10.1177/0306624X10370452

[cl21377-bib-0079] Harkins, C. , Lisa, G. , Aileen, C. , & Carol, T. (2016). Hitting the right note for child and adolescent mental and emotional wellbeing: A formative qualitative evaluation of Sistema Scotland's “Big Noise” orchestral programme. Journal of Public Mental Health, 15(1), 25–36.

[cl21377-bib-0080] Harris, N. , Leigh, W. , & Donald, S. (2012). HYPEd‐up: Youth dance culture and health. Arts & Health, 4(3), 239–248.

[cl21377-bib-0081] Harris, D. A. , & Stephanie, M. (2014). A process evaluation of the art of yoga project mentor program for incarcerated teenage girls. International Journal of Yoga Therapy, 24, 97–108. 25858656

[cl21377-bib-0082] Head, G. , & Angela, J. (2015). “I've taken confidence away from this”: The experiences and impact of moving image education with young people who require more choices, more chances. Citizenship, Social and Economics Education, 14(3), 159–171.

[cl21377-bib-0083] Henley, J. (2015). Prisons and primary schools: Using CHAT to analyse the relationship between developing identity, developing musicianship and transformative processes. British Journal of Music Education, 32(2), 123–141.

[cl21377-bib-0084] Hess, J. (2019). Music education for social change: Constructing an activist music education (1, pp. 1–200). Routledge.

[cl21377-bib-0085] Hon, S. (2021). *The relevance of cultural arts practices to the psychosocial well‐being of adolescents affected by violence in Trinidad and Tobago [PhD Dissertation]*, 1, 1–179.

[cl21377-bib-0086] Hoogsteder Larissa, M. , Stams Geert‐Jan, J. M. , Schippers Eveline, E. , & Bonnes, D. (2018). Responsive aggression regulation therapy (Re‐ART): An evaluation study in a Dutch juvenile justice institution in terms of recidivism. International Journal of Offender Therapy and Comparative Criminology, 62(14), 4403–4424. 29504484 10.1177/0306624X18761267

[cl21377-bib-0087] Kallio Alexis, A. , & Heidi, W. (2016). The ethics of survival: Teaching the traditional arts to disadvantaged children in post‐conflict Cambodia. International Journal of Music Education, 34(1), 90–103.

[cl21377-bib-0088] Kallio, A. A. (2022). The transformative potentials and politics of music in juvenile justice settings. Music Education Research, 24(4), 405–416.

[cl21377-bib-0089] Kim, J. (2015). Music therapy for deprived children who are exposed to childhood abuse and absolute poverty. Nordic Journal of Music Therapy, 24(1), 27–43.

[cl21377-bib-0090] Lai, J. X. (Gene) (2020). Uṟumi Mēḷam: A musical sanctuary for at‐risk Tamil youth in Singapore. Ethnomusicology Forum, 29(3), 311–332.

[cl21377-bib-0091] La Porte, A. (2016). Efficacy of the arts in a transdisciplinary learning experience for culturally diverse fourth graders. International Electronic Journal of Elementary Education, 8(3), 467–480.

[cl21377-bib-0092] Lev‐Aladgem, S. (2010). Public theatre, community theatre, and collaboration: Two case studies. New Theatre Quarterly, 26(4), 369–382.

[cl21377-bib-0093] Logie, C. H. , Moses, O. , Odong, L. S. , Miranda, L. , Alyssa, M. A. , Maya, L. , Isha, B. , Nelson, K. , Simon, M. , Peter, K. , Stella, N. , Eusebius, S. , Moses, B. S. , & Joshua, M. (2021). Ngutulu Kagwero (agents of change): Study design of a participatory comic pilot study on sexual violence prevention and post‐rape clinical care with refugee youth in a humanitarian setting in Uganda. Global Health Action, 14(1), 1940763. 34402763 10.1080/16549716.2021.1940763 PMC8381980

[cl21377-bib-0094] McCann, R. , & Peters Cynthia, D. (1996). At‐risk youth: The Phoenix phenomenon. Journal of Physical Education, Recreation & Dance, 67(2), 38–40.

[cl21377-bib-0095] McCrary . (2019). Identifying various gang prevention and intervention program to reduce gang violence and understand why youth join gangs. Law Enforcement Executive Forum, 19(1), 30–34.

[cl21377-bib-0096] McCray, E. D. , Cecelia, R. , Holly, L. , Murphy Kristin, M. , Gagnon Joseph, C. , Houchins David, E. , & Lambert Richard, G. (2018). ‘As real as it gets’: A grounded theory study of a reading intervention in a juvenile correctional school. Child & Youth Care Forum, 47, 259–281.

[cl21377-bib-0097] Meekums, B. , & Jennifer, D. (2011). Arts with offenders: A literature synthesis. The Arts in Psychotherapy, 38(4), 229–238.

[cl21377-bib-0098] Milner, J. (2000). Arts impact: Improving the odds for at‐risk youth. Performing Arts & Entertainment in Canada, 33(2), 11.

[cl21377-bib-0099] Miner‐Romanoff, K. (2016). Voices from inside. Journal of Correctional Education (1974), 67(1), 58–74.

[cl21377-bib-0100] Mohler, C. E. (2012). How to turn “a bunch of gang‐bangin' criminals into big kids having fun”: Empowering incarcerated and at‐risk youth through ensemble theatre. Theatre Topics, 22(1), 89–102.

[cl21377-bib-0101] Morehouse, E. , & Tobler, N. S. (2000). Preventing and reducing substance use among institutionalized adolescents. Adolescence, 35, 137. 10841294

[cl21377-bib-0102] Moyer Jeffrey, S. , Warren Mark, R. , & King Andrew, R. (2020). “Our stories are powerful”: The use of youth storytelling in policy advocacy to combat the School‐to‐Prison pipeline. Harvard Educational Review, 90(2), 172–194.

[cl21377-bib-0103] Mukherjee, M. , & Nirban, M. (2021). Indian theatre and incarceration: Performing the transition from criminal to civic space. Research in Drama Education: The Journal of Applied Theatre and Performance, 26(3), 528–541.

[cl21377-bib-0104] Nelson Larry, P. , McMahan Sarah, K. , & Torres, T. (2012). The impact of a junior high school community intervention project: Moving beyond the testing juggernaut and into a community of creative learners. School Community Journal, 22(1), 125–144.

[cl21377-bib-0105] Nind, M. , Boorman, G. , & Clarke, G. (2012). Making schools fitting places for all: A creative approach for girls excluded from mainstream education. In Transforming troubled lives: Strategies and interventions for children with social, emotional and behavioural difficulties Emerald Group Publishing Limited (Vol. 2, pp. 289–307).

[cl21377-bib-0106] Oesterreich Heather, A. , & Flores Sara, M. N. (2009). Learning to C: Visual arts education as strengths based practice in juvenile correctional facilities. Journal of Correctional Education, 60(2), 146–162.

[cl21377-bib-0107] Oosthuizen, H. (2019). The potential of paradox: Chaos and order as interdependent resources within short‐term music therapy groups with young offenders in South Africa. Qualitative Inquiries in Music Therapy, 14(1), 1‐0_1.

[cl21377-bib-0108] Pane, D. M. (2015). The story of drama club: A contemporary counternarrative of a transformative culture of teaching and learning for disenfranchised Black youth in the school‐to‐prison pipeline. Remie – Multidisciplinary Journal Of Educational Research, 5(3), 242–267.

[cl21377-bib-0109] Payne, B. , Hobson, J. , & Lynch, K. (2021). ‘We just want to be treated with respect!’: Using restorative approaches and the dramatic arts to build positive relationships between the police and young people. Youth Justice – An International Journal, 21(3), 255–274.

[cl21377-bib-0110] Pyles, D. G. (2017). A social semiotic mapping of voice in youth media: The pitch in youth video production. Learning, Media and Technology, 42(1), 8–27.

[cl21377-bib-0111] Qiu, H.‐Z. , Ye, Z.‐J. , Liang, M.‐Z. , Huang, Y.‐Q. , Liu, W. , & Lu, Z.‐D. (2017). Effect of an art brut therapy program called go beyond the schizophrenia (GBTS) on prison inmates with schizophrenia in mainland China—A randomized, longitudinal, and controlled trial. Clinical psychology & psychotherapy, 24(5), 1069–1078. 28078741 10.1002/cpp.2069

[cl21377-bib-0112] Rapp‐Paglicci, L. , Stewart, C. , & Rowe William, S. (2009). Evaluating the effects of the prodigy cultural arts program on symptoms of mental health disorders in at‐risk and adjudicated youths. Best Practices in Mental Health, 5(1), 65–73.

[cl21377-bib-0113] Savage, J. , & Challis, M. (2002). A digital arts curriculum? Practical ways forward. Music Education Research, 4(1), 7–23.

[cl21377-bib-0114] Schaillée, H. , Theeboom, M. , & Skille, E. (2017). Adolescent girls’ experiences of urban dance programmes: A qualitative analysis of Flemish initiatives targeting disadvantaged youth. European Journal for Sport and Society, 14(1), 26–44.

[cl21377-bib-0115] Sheltzer Joshua, M. , & Consoli Andrés, J. (2019). Understanding the impact of an after‐school music program with engaged underserved youth. Journal of Community Psychology, 47(6), 1364–1379. 31017308 10.1002/jcop.22193

[cl21377-bib-0116] Sieber, T. , Cordeiro Graça, Í. , & Ferro, L. (2012). The neighborhood strikes back: Community murals by youth in Boston's communities of color the neighborhood strikes back: Community murals by youth in Boston's communities of color. City & Society, 24(3), 263–280.

[cl21377-bib-0117] Simmons, R. (2017). Employability, knowledge and the creative arts: Reflections from an ethnographic study of NEET young people on an entry to employment programme. Research in Post‐Compulsory Education, 21(1), 22–37.

[cl21377-bib-0118] Tam, H.‐L. , Wai‐yan, S. A. , & Siu‐ling, L. S. (2016). Using expressive arts in relapse prevention of young psychotropic substance abusers in Hong Kong. Children and Youth Services Review, 26(60), 88–100.

[cl21377-bib-0119] Thompson, I. , & Alice, T. (2017). Becoming other: Social and emotional development through the creative arts for young people with behavioural difficulties. Emotional & Behavioural Difficulties, 22(1), 18–34.

[cl21377-bib-0120] van Niekerk, C. , & Maria, T. (2012). STTEP by STEPP in the spirit of “Umuntu Ungumuntu Ngabantu”. British Journal of Music Education, 29(1), 75–89.

[cl21377-bib-0121] Watson, D. W. , Mouttapa, M. , Reiber, C. , McCuller William, J. , Arancibi, R. , Kavich Julia, A. , Nieves, E. , Novgrod, J. , Mai, N. , Bisesi, L. , & Sim, T. (2007). The life interventions for family effectiveness (LIFE) project: Preliminary findings on alternative school intervention for adolescents. Journal of Correctional Education (1974), 58(1), 57–68.

[cl21377-bib-0122] Wright, P. , Down, B. , & Davies, C. (2022). Learning, making and flourishing in non‐formal spaces: Participatory arts and social justice. Education, Citizenship and Social Justice, 17(1), 54–68.

[cl21377-bib-0123] ONGOING STUDIES

[cl21377-bib-0124] ZLOTOWITZ PhD 2010

[cl21377-bib-0125] OTHER REFERENCES

[cl21377-bib-0126] ADDITIONAL REFERENCES

[cl21377-bib-0127] Anderson, K. , & Overy, K. (2010). Engaging Scottish young offenders in education through music and art. International Journal of Community Music, 3(1), 47–64. 10.1386/ijcm.3.1.47/1

[cl21377-bib-0128] Campbell, M. , McKenzie, J. E. , Sowden, A. , Katikireddi, S. V. , Brennan, S. E. , Ellis, S. , Hartmann‐Boyce, J. , Ryan, R. , Shepperd, S. , Thomas, J. , Welch, V. , & Thomson, H. (2020). Synthesis without meta‐analysis (SWiM) in systematic reviews: Reporting guideline. BMJ, 368, l6890. 31948937 10.1136/bmj.l6890 PMC7190266

[cl21377-bib-0129] Chen, X. J. , Leith, H. , Aarø, L. E. , Manger, T. , & Gold, C. (2016). Music therapy for improving mental health problems of offenders in correctional settings: Systematic review and meta‐analysis. Journal of Experimental Criminology, 12, 209–228. 10.1007/s11292-015-9250-y

[cl21377-bib-0130] Canadian Health Libraries Association . (2022). Adolescents and young adults: Search filter. Retrieved 25 October 2009. https://extranet.santecom.qc.ca/wiki/!biblio3s/doku.php?id=concepts:adolescents-et-jeunes-adultes

[cl21377-bib-0131] Daykin, N. , De Viggiani, N. , Pilkington, P. , & Moriarty, Y. (2012). Music making for health, well‐being and behaviour change in youth justice settings: A systematic review. Health Promotion International, 28(2), 97–210. 10.1093/heapro/das005 22415559

[cl21377-bib-0132] Daykin, N. , De Viggiani, N. , Pilkington, P. , & Moriarty, Y. (2013). Music making for health, well‐being and behaviour change in youth justice settings: A systematic review. Health Promotion International, 1, 197–210. 10.1093/heapro/das005 22415559

[cl21377-bib-0133] Daykin, N. , DeViggiani, N. , Moriarty, Y. , & Pilkington, P. (2017). Music making for health and wellbeing in youth justice settings: Mediated affordances and the impact of context and social relations. Sociology of Health and Illness, 39(6), 941–958. 28332197 10.1111/1467-9566.12549

[cl21377-bib-0134] Deeks, J. J. , Higgins, J. P. T. , & Altman, D. G. (2020). Analysing data and undertaking meta‐analyses. In J. P. T. Higgins , J. Thomas , J. Chandler , M. Cumpston , T. Li , & M. J. Page , et al. (Eds.), Cochrane Handbook for Systematic Reviews of Interventions. 6.1 edition. Cochrane. http://www.training.cochrane.org/handbook

[cl21377-bib-0135] Frater, A. (2019). Unlock imagined: Arts in criminal justice. The International Journal of Forensic Psychotherapy, 1(2), 131–143.

[cl21377-bib-0136] HAQ Centre for Child Rights . Juvenile justice in different countries age of criminal responsibility and treatment of juvenile offenders. A compilation. Accessed February 1, 2022. https://www.slideshare.net/HAQCRCIndia/juvenile-justice-in-different-countries-age-of-criminal-responsibility-and-treatment-of-juvenile-offenders

[cl21377-bib-0137] Higgins, J. P. T. , Altman, D. G. , & Sterne, J. A. C. Chapter 8: Assessing risk of bias in included studies. In J. P. T. Higgins , & S. Green (Eds.), Cochrane Handbook for Systematic Reviews of Interventions Version 5.1.0. The Cochrane Collaboration.

[cl21377-bib-0138] Higgins, J. P. T. , Eldridge, S. , & Li, T. (2021). Including variants on randomized trials. In J. P. T. Higgins , S. Eldridge , & T. Li (Eds.), Chapter 23. Cochrane Handbook for Systematic Reviews of Interventions. 6.2 edition. Cochrane. http://www.training.cochrane.org/handbook

[cl21377-bib-0139] Hoffmann, T. C. , Glasziou, P. P. , Boutron, I. , Milne, R. , Perera, R. , Moher, D. , Altman, D. G. , Barbour, V. , Macdonald, H. , Johnston, M. , & Lamb, S. E. (2014). Better reporting of interventions: Template for intervention description and replication (TIDieR) checklist and guide. BMJ, 7, 348. 10.1136/bmj.g1687 24609605

[cl21377-bib-0140] Kugley, S. , Wade, A. , Thomas, J. , Mahood, Q. , Jørgensen, A. M. , Hammerstrøm, K. , & Sathe, N. (2016). Searching for studies: A guide to information retrieval for Campbell. Campbell Systematic Reviews. Searching for studies: Guidelines on information retrieval for Campbell Systematic Reviews (campbellcollaboration.org). 10.1002/cl2.1433 PMC11386270 39258215

[cl21377-bib-0141] Lewin, S. , Glenton, C. , Munthe‐Kaas, H. , Carlsen, B. , Colvin, C. J. , Gülmezoglu, M. , Noyes, J. , Booth, A. , Garside, R. , & Rashidian, A. (2015). Using qualitative evidence in decision making for health and social interventions: An approach to assess confidence in findings from qualitative evidence syntheses (GRADE‐CERQual). PLoS Medicine, 12(10), e1001895. 10.1371/journal.pmed.1001895 26506244 PMC4624425

[cl21377-bib-0142] Mansfield, L. , Daykin, N. , O'Connell, N. E. , Bailey, D. , Forde, L. , Smith, R. , & Gifford, J. (2023). PROTOCOL: A mixed methods systematic review on the effects of arts interventions for at‐risk and offending children and young people on behavioural, psychosocial, cognitive and offending outcomes [Protocol]. Cochrane Database of Systematic Reviews, 19(1), e1298. 10.1002/cl2.1298 PMC9831279 36911860

[cl21377-bib-0143] Marttunen, M. (2004). Finland/The basis of Finnish juvenile criminal justice. Revue internationale de droit pénal, 75, 315–335.

[cl21377-bib-0144] Meekums, B. , & Daniel, J. (2011). Arts with offenders: A literature synthesis. The Arts in Psychotherapy, 38(4), 229–238.

[cl21377-bib-0145] Moore, G. F. , Audrey, S. , Barker, M. , Bond, L. , Bonell, C. , Hardeman, W. , Moore, L. , O'Cathain, A. , Tinati, T. , Wight, D. , & Baird, J. (2015). Process evaluation of complex interventions: Medical Research Council guidance. BMJ, 19, 350. 10.1136/bmj.h1258 PMC4366184 25791983

[cl21377-bib-0146] Noyes, J. , Booth, A. , Flemming, K. , Garside, R. , Harden, A. , Lewin, S. , Pantoja, T. , Hannes, K. , Cargo, M. , & Thomas, J. (2018). Cochrane Qualitative and Implementation Methods Group guidance series—paper 3: Methods for assessing methodological limitations, data extraction and synthesis, and confidence in synthesized qualitative findings. Journal of Clinical Epidemiology, 97, 49–58. 10.1016/j.jclinepi.2017.06.020 29247700

[cl21377-bib-0147] Schünemann, H. J. , Higgins, J. P. , Vist, G. E. , Glasziou, P. , Akl, E. A. , & Skoetz, N. , on behalf of the Cochrane GRADEing Methods Group (formerly Applicability and Recommendations Methods Group) and the Cochrane Statistical Methods Group . (2020). Chapter 14. ‘Summary of findings’ tables and grading the certainty of the evidence. In J. P. Higgins , J. Thomas , J. Chandler , M. Cumpston , T. Li , & M. J. Page (Eds.), Cochrane Handbook for Systematic Reviews of Interventions version 6.1 (updated September 2020). Cochrane. http://www.training.cochrane.org/handbook

[cl21377-bib-0148] Slade, S. C. , Dionne, C. E. , Underwood, M. , Buchbinder, R. , Beck, B. , Bennell, K. , Brosseau, L. , Costa, L. , Cramp, F. , Cup, E. , & Feehan, L. (2016). Consensus on exercise reporting template (CERT): Modified Delphi study. Physical Therapy, 96(10), 1514–1524. 10.2522/ptj.20150668 27149962

[cl21377-bib-0149] Thomas, J. , & Harden, A. (2008). Methods for the thematic synthesis of qualitative research in systematic reviews. BMC Medical Research Methodology, 8(1), 1–10. 18616818 10.1186/1471-2288-8-45 PMC2478656

[cl21377-bib-0150] University of Texas School of Public Health . (2022). Search filters for various databases: OVID MEDLINE qualitative studies. Retrieved 25 October 2022. https://libguides.sph.uth.tmc.edu/search_filters/ovid_medline_filters

[cl21377-bib-0151] Waffenschmidt, S. , Navarro‐Ruan, T. , Hobson, N. , Hausner, E. , Sauerland, S. , & Haynes, R. B. (2020). Development and validation of study filters for identifying controlled non‐randomized studies in PubMed and Ovid MEDLINE. Research Synthesis Methods, 11(5), 617–626. 32472632 10.1002/jrsm.1425

[cl21377-bib-0152] Wagner, M. , Rosumeck, S. , Küffmeier, C. , Döring, K. , & Euler, U. (2020). A validation study revealed differences in design and performance of MEDLINE search filters for qualitative research. Journal of Clinical Epidemiology, 2020(120), 17–24. 10.1016/j.jclinepi.2019.12.008 31862229

[cl21377-bib-0153] World Health Organisation . (2015). Preventing Youth Violence: An Overview of the Evidence. WHO Report 2015. https://www.who.int/publications/i/item/preventing-youth-violence-an-overview-of-the-evidence

[cl21377-bib-0154] Youth Justice Board/Ministry of Justice . (2021). Youth Justice Statistics 2019/20 England and Wales. Youth Justice statistics. UK Government Report 2019. GOV.UK (www.gov.uk).

[cl21377-bib-0155] Other published versions of this review

[cl21377-bib-0156] Classification pending references

