# Supplementary material for: A mixed methods systematic review on the effects of arts interventions for children and young people at‐risk of offending, or who have offended on behavioural, psychosocial, cognitive and offending outcomes: A systematic review
Source: Campbell Syst Rev. 2024 Jan 3;20(1):e1377. doi: 10.1002/cl2.1377 (PMC10765125; doi:10.1002/cl2.1377)
Supplement: Supplementary file 2 — Supporting information. [file CL2-20-e1377-s001.docx]

# Published notes

# Characteristics of studies

## Characteristics of included studies

### Anderson 2010

| **Methods** | Non randomised controlled trial with a qualitative component.  Qualitative data collection: Structured interviews 13 participants post intervention  Analysis/ Theoretical perspective: Not reported. |
| --- | --- |
| **Participants** | Young males in YOI N=30 (14 completers) |
| **Interventions and comparisons (where applicable)** | Intervention group:  Music classes N=4,  Art classes (sculpture) N=5  Aim of the sessions was to engage the men in creating music and art within a group setting – combination of individual and group practice.  8 weeks duration.  Comparison group:  Educational control. Usual practice. Classes in Numeracy & Maths or Communication & Literacy N=5 |
| **Outcomes** | Behaviour incidents  Engagement with education  Self Esteem  Locus of control  Emotion |
| **Study Setting** | Young offenders institution (YOI), UK |
| **Notes** |  |

#### Risk of bias table

| **Item** | **Authors' judgement** | **Support for judgement** |
| --- | --- | --- |
| Random sequence generation | High risk | Allocation not randomised |
| Allocation concealment | High risk | Allocation not concealed |
| Blinding of participants and practitioners | High risk | Participants and practitioners not blinded |
| Blinding of assessors | High risk | Assessors not blinded |
| Incomplete outcome data | High risk | 53% of participants lost to follow-up. Not accounted for in analysis |
| Selective outcome reporting | Unclear risk | No protocol or registration record |
| Are the data free of carry-over effects? | Unclear risk |  |

### Atherton 2022

| **Methods** | Study Design: Qualitative  Data Collection: Semi-structured interviews  Analysis/Theoretical Perspective: Thematic Analysis |
| --- | --- |
| **Participants** | N = 16 Arts students (N= 10men in the prison and N= 6 men and women in the community) |
| **Interventions and comparisons (where applicable)** | Intervention:  Soft Touch Arts based workshops called *Unlocked* in prison and community settings.  The workshops adopt social prescribing. Lead artists facilitate the participants to shape their own learning and create their own meaningful artworks.  The program followed the principles of Leamy et al.’s (2011) CHIME framework  In the prison setting, weekly sessions dovetailed with another project which focused on music production.  Comparison: None - qualitative study |
| **Outcomes** | Outcomes: N/A Qualitative Study  Key Processes:  Positive emotions (hope and aspiration) through choice/capability approach  Learning new skills  Redefining sense of self and success and self-care  Attention to prison environment, flexibility, skills facilitators  Social interaction for shared learning, relief from boredom  Focus on creativity as meaning and purpose  Focus on creative practice as a means of community change; supporting reintegration; considering employment |
| **Study Setting** | Two adult male closed prisons in the UK and a community setting based at the arts project headquarters (UK) |
| **Notes** |  |

#### Risk of bias table

| **Item** | **Authors' judgement** | **Support for judgement** |
| --- | --- | --- |
| Random sequence generation | Unclear risk |  |
| Allocation concealment | Unclear risk |  |
| Blinding of participants and practitioners | Unclear risk |  |
| Blinding of assessors | Unclear risk |  |
| Incomplete outcome data | Unclear risk |  |
| Selective outcome reporting | Unclear risk |  |
| Are the data free of carry-over effects? | Unclear risk |  |

### Baker 2007

| **Methods** | Study Design: Qualitative  Data Collection: Observations  Method of Analysis or theoretical perspective : Not reported |
| --- | --- |
| **Participants** | Male (predominantly black) juvenile offenders who had been sentenced to a short-term secure treatment centre. |
| **Interventions and comparisons (where applicable)** | Intervention:  Music programme aimed to teach musical composition and computer-based music sequencing to incarcerated youth as a way to assist in the development of their ability to ‘positive life decisions’. Lessons with individuals/small groups.  Comparison:  None - qualitative |
| **Outcomes** | Outcomes: N/A Qualitative Study  Key Processes:  Positive reflection, skill development, rap perceived as culturally relevant and authentic by participants, critical engagement of expert facilitator |
| **Study Setting** | Short-term secure detention/treatment centre (USA) |
| **Notes** |  |

#### Risk of bias table

| **Item** | **Authors' judgement** | **Support for judgement** |
| --- | --- | --- |
| Random sequence generation | Unclear risk |  |
| Allocation concealment | Unclear risk |  |
| Blinding of participants and practitioners | Unclear risk |  |
| Blinding of assessors | Unclear risk |  |
| Incomplete outcome data | Unclear risk |  |
| Selective outcome reporting | Unclear risk |  |
| Are the data free of carry-over effects? | Unclear risk |  |

### Barrett 2012

| **Methods** | Study design: Qualitative  Data collection: 3 x Individual and group interviews with 17 students (last interview was an artefact-elicited interview)  3 x Individual interviews with 3 key stake- holders (pre- programme, during, and after)  Observations of music lessons  Analysis/Theoretical Perspective: Iterative analysis, member checking with participants. No further detail reported. |
| --- | --- |
| **Participants** | Residents of an Australian Juvenile Detention Centre. N=17  Age range 14-18  Period in detention range 1-13 months  Length of intervention participantion range: 0.5- 5 months.  3 stakeholders -ACMF music teacher. AJDC principal, programme manager at AJDC. |
| **Interventions and comparisons (where applicable)** | Music programme delivered in the Detention Centrethrough the Australia Children’s Music Foundation.  Weekly & small group music sessions. |
| **Outcomes** | N/A Qualitative Study |
| **Study Setting** | Australian juvenile detention centre: AJDC is a small facility in a rural setting in Australia. AJDC serves as both a remand and detention facility. |
| **Notes** |  |

#### Risk of bias table

| **Item** | **Authors' judgement** | **Support for judgement** |
| --- | --- | --- |
| Random sequence generation | Unclear risk |  |
| Allocation concealment | Unclear risk |  |
| Blinding of participants and practitioners | Unclear risk |  |
| Blinding of assessors | Unclear risk |  |
| Incomplete outcome data | Unclear risk |  |
| Selective outcome reporting | Unclear risk |  |
| Are the data free of carry-over effects? | Unclear risk |  |

### Barrett 2015

| **Methods** | Study design: Qualitative  Data Collection: 9-month collective case study  3 visits to each school over a 3 -month period  Interviews with parents, participants and adults and participant observation pre project, during and after  Analysis/ Theoretical Perspective: Inductive then deductive analysis using the Five C’s Positive Youth Development Framework: Competence, Confidence,Connection, Character and Caring. |
| --- | --- |
| **Participants** | Australian Children Music Foundation music programmes delivered in 4 schools in NSW, Australia.  School A. Ethnically diverse school in a disadvantaged community. (N=10 students, N=2 adults, N=2 parents).  School B. Regional school. Pts aged from Kindergarden to year 6 (N=10 students, N=3 adults, N=2 parents).  School C: Students years 5-10. (N=4 students, N=3 adults).  School D:  Urban comprehensive school which provides classes for students with intellectual difficulties. (N=4 students, N=3 adults)  Total: N=28 Students, N=9 staff, N=4 participant parents |
| **Interventions and comparisons (where applicable)** | Australian Children Music Foundation music programmes delivered in 4 schools in NSW, Australia.  School A: weekly music lessons which include: singing, drumming, performing instrumental accompaniments to vocal works, musical literacy and auditory perception games, and movement experiences.  School B: Intensive music lessons two weeks each term. In between visits students can access lesson plans and resources. Music lessons include rhythm and musical memory games, reading simple musical notation, instrument practice, classroom singing & drumming.  School C: Informal small group music classes  School D: Weekly lunchtime song writing class with 5-10 students. |
| **Outcomes** | N/A Qualitative Study |
| **Study Setting** | Australian Children Music Foundation music programmes delivered in 4 schools in NSW, Australia.  School A. Ethnically diverse school in a disadvantaged community.  School B. Regional school. Pts aged from Kindergarden to year 6  School C: Reintegration school for students with emotional disturbances and behavioural difficulties who previously attended mainstream schools. Students years 5-10  School D:  Urban comprehensive school which provides classes for students with intellectual difficulties. |
| **Notes** |  |

#### Risk of bias table

| **Item** | **Authors' judgement** | **Support for judgement** |
| --- | --- | --- |
| Random sequence generation | Unclear risk |  |
| Allocation concealment | Unclear risk |  |
| Blinding of participants and practitioners | Unclear risk |  |
| Blinding of assessors | Unclear risk |  |
| Incomplete outcome data | Unclear risk |  |
| Selective outcome reporting | Unclear risk |  |
| Are the data free of carry-over effects? | Unclear risk |  |

### Bittman 2009

| **Methods** | Randomised cross-over study |
| --- | --- |
| **Participants** | "At risk" adolescent and teen residents.  N=52  30 F, 22 M |
| **Interventions and comparisons (where applicable)** | Intervention condition:  Recreational Music Making, 6 weeks, x1 weekly  Control condition:  Usual practice. "normal structured routines that included therapeutic and educational programs focusing on current events, independent living, housing, social skills, grief and loss, health, drug and alcohol use, employment, sexual abuse/sexuality, anger management, and conflict resolution." |
| **Outcomes** | Level of functioning  Psychopathology  Anger  Depression |
| **Study Setting** | Bethesda childrens home. Secure residential treatment facility. USA |
| **Notes** |  |

#### Risk of bias table

| **Item** | **Authors' judgement** | **Support for judgement** |
| --- | --- | --- |
| Random sequence generation | Unclear risk | Method of randomisation not reported. |
| Allocation concealment | Unclear risk |  |
| Blinding of participants and practitioners | High risk | Participants and practitioners not blinded |
| Blinding of assessors | High risk | Assessors not blinded |
| Incomplete outcome data | High risk | Per protocol analysis, 13% attrition due to non-attendance resulting from illness, discharge, or physician appointments. |
| Selective outcome reporting | High risk | No protocol or registration record available. |
| Are the data free of carry-over effects? | High risk | No washout period observed. No baseline adjustment. signs of some baseline imbalance on CAFAS. |

### Bowey 2006

| **Methods** | Study design: Qualitative  Data collection: Individual interviews with N=11 young people within one week of the intervention.  Analysis/ Theoretical Perspective: Not reported. |
| --- | --- |
| **Participants** | 18 YP recruited and commenced the intervention.  Full data sets for 11 YP, year nine pupils who met the criteria of being excluded or at risk of being excluded, and/or having offended or showing the potential for offending. |
| **Interventions and comparisons (where applicable)** | Video pilot intervention feasibility study in Nottingham, UK Aimed to improve attitudes to crime and the police, to reduce exclusion, and to develop self-esteem in young people at risk of offending and/or school exclusion.  The intervention was run over six days. The project focused mainly on issues around crime, and resulted in each group producing a video. |
| **Outcomes** | N.A Qualitative Study |
| **Study Setting** | School based. UK |
| **Notes** |  |

#### Risk of bias table

| **Item** | **Authors' judgement** | **Support for judgement** |
| --- | --- | --- |
| Random sequence generation | Unclear risk |  |
| Allocation concealment | Unclear risk |  |
| Blinding of participants and practitioners | Unclear risk |  |
| Blinding of assessors | Unclear risk |  |
| Incomplete outcome data | Unclear risk |  |
| Selective outcome reporting | Unclear risk |  |
| Are the data free of carry-over effects? | Unclear risk |  |

### Caulfield 2019

| **Methods** | Study Design: Qualitative (Mixed Methods; grey literature)  Data Collection: Semi structured interviews  Analysis/Theoretical Perspective: Thematic analysis (details of approach not reported) |
| --- | --- |
| **Participants** | N=8 young people (demographic details of sample not reported |
| **Interventions and comparisons (where applicable)** | Intervention  Multiple arts interventions as part of Sandwell UK services taken up by young people as choice including drawing, photography, pottery, nail art, up cycling graffiti (for carers), media project, music programme and bespoke activities e.g. quilt making, spoken word and painting.  Comparison: None - qualitative study |
| **Outcomes** | Outcomes: N/A Qualitative Study  Key Processes:  Facilitation engagement of YP (practicalities, person centred breaking down barriers)  Staff involvement (engagement, workloads, sustainability, internal communication)  External partnerships (development)  Personal impact (confidence, wellbeing, attitude to cerative activity)  Skill development (creative and communications)  Better relationships (staff and peers)  New opportunities and aspirations (aspirations and plans for future) |
| **Study Setting** | Local community |
| **Notes** |  |

#### Risk of bias table

| **Item** | **Authors' judgement** | **Support for judgement** |
| --- | --- | --- |
| Random sequence generation | Unclear risk |  |
| Allocation concealment | Unclear risk |  |
| Blinding of participants and practitioners | Unclear risk |  |
| Blinding of assessors | Unclear risk |  |
| Incomplete outcome data | Unclear risk |  |
| Selective outcome reporting | Unclear risk |  |
| Are the data free of carry-over effects? | Unclear risk |  |

### Caulfield 2022

| **Methods** | Non randomised comparative study with qualitative component.  Qualitative methods:  Data Collection: Semi-structured interviews with N=23 participants.  Analysis/ Theoretical Perspective: Thematic analysis. |
| --- | --- |
| **Participants** | Young offenders.  participants were referred by their YOT caseworker. N=42  3 F, 39 M |
| **Interventions and comparisons (where applicable)** | Intervention group:  Music programme incl. production skills and composition to performance skills and music tuition  12 weeks, x1 weekly  Comparison group:  Cohort of children who did not attend programme. N=145 |
| **Outcomes** | Attitude and behaviour  Wellbeing  Attendance |
| **Study Setting** | Young offenders service. UK |
| **Notes** |  |

#### Risk of bias table

| **Item** | **Authors' judgement** | **Support for judgement** |
| --- | --- | --- |
| Random sequence generation | High risk | Allocation not randomised |
| Allocation concealment | High risk | Allocation not concealed |
| Blinding of participants and practitioners | High risk | Participants and Practitioners not blinded |
| Blinding of assessors | Unclear risk | Blinding of assessors not reported |
| Incomplete outcome data | Unclear risk | Attrition not clearly reported. |
| Selective outcome reporting | High risk | No protocol or registration record available. Not between group comparison reported for key outcomes. |
| Are the data free of carry-over effects? | Unclear risk |  |

### Cesar 2020

| **Methods** | Study design: Qualitative  Data collection: Interviews  Analysis/Theoretical Perspective: Two cycle thematic analysis. First-cycle “theory coding” identified narratives about social support: family, role models, mentorship, the arts mentorship organization (AMO), and plans for the future. The second cycle consisted of “pattern analysis” within each theory code to identify emergent themes that characterized the narratives. |
| --- | --- |
| **Participants** | Children in child protection system placements.  N=7 male, N=26 female  Mean (SD) age 14.9 (1.32 ) years |
| **Interventions and comparisons (where applicable)** | Intervention: Theatre Camp involved active participation, requiring youth to tell their stories and cooperate in creating, staging, and performing dances, poems, and songs. Delivered by a local arts mentorship organization.  Comparison: None - qualitative study |
| **Outcomes** | Outcomes: N/A Qualitative Study  Key processes: friendship and community, environment of care, programme fills a gap left by state funded services, development of brave spaces, creativity, development of soft skills in the camp but not a protective factor for life. |
| **Study Setting** | Theater camp (USA) |
| **Notes** |  |

#### Risk of bias table

| **Item** | **Authors' judgement** | **Support for judgement** |
| --- | --- | --- |
| Random sequence generation | Unclear risk |  |
| Allocation concealment | Unclear risk |  |
| Blinding of participants and practitioners | Unclear risk |  |
| Blinding of assessors | Unclear risk |  |
| Incomplete outcome data | Unclear risk |  |
| Selective outcome reporting | Unclear risk |  |
| Are the data free of carry-over effects? | Unclear risk |  |

### Chong 2020

| **Methods** | Study Design: Qualitative  Data Collection: Interviews  Analysis/Theoretical Perspective: Content analysis |
| --- | --- |
| **Participants** | 178 adolescents involved with the juvenile justice system: 115 on suspension of indictment and 63 under supervised probation. 163 were male and 15 were female. Average age was 17.5 years.  Interviews took place with n=20 participants. |
| **Interventions and comparisons (where applicable)** | Intervention: Young & Great Music Project. 15-session program that is delivered in four stages: Developing relationship with music and others and increasing self-awareness through the music experience (Sessions 1 through 3); Identifying one’s resources and strengths (Sessions 4 through 8),; Identifying challenges and learning to use strategic resources (Sessions 9 through 12),; Empowering one’s own inner strength through self-actualization experiences in music (Session 13 through 15).  Comparison: None - qualitative study |
| **Outcomes** | Outcomes: N/A Qualitative Study  Key Processes: relationship building, programme conceptualises at-risk youth beyond the individual/family and instead as belonging to the wider community of regional partners who can provide guidance and support to help young people be contributing members of society. |
| **Study Setting** | South Korea |
| **Notes** |  |

#### Risk of bias table

| **Item** | **Authors' judgement** | **Support for judgement** |
| --- | --- | --- |
| Random sequence generation | Unclear risk |  |
| Allocation concealment | Unclear risk |  |
| Blinding of participants and practitioners | Unclear risk |  |
| Blinding of assessors | Unclear risk |  |
| Incomplete outcome data | Unclear risk |  |
| Selective outcome reporting | Unclear risk |  |
| Are the data free of carry-over effects? | Unclear risk |  |

### Clennon 2015

| **Methods** | Study Design: Qualitative  Data Collection: Performance ethnography, Participatory tools (writing, performing and recording MC (rap) tracks)  Analysis/Theoretical Perspective: Thematic analysis of lyric sketches and session notes. |
| --- | --- |
| **Participants** | N = 15 young men, aged between 16 and 18, working in groups of eight.  N = 7 core group engaged over 20 weeks. |
| **Interventions and comparisons (where applicable)** | Intervention:  A 20-week music technology project: delivered between 2006 and 2007 by a multidisciplinary staff team of artists, researchers and a prison education officer, who integrated the work with the institution’s education programme.   Participants undertook creative writing, mind mapping and team- based music technology activities in which they played out ‘roles’ such as artist, engineer, producer, songwriter and mentor.   Discussions focused on beliefs and attitudes towards issues such as gun crime and ideological and lyrical thinking. The programme included an activity in which trainees planned their futures upon leaving custody by constructing resettlement packages comprising contacts for follow- up projects or educational opportunities.  Comparison: None - qualitative study |
| **Outcomes** | Outcomes: N/A Qualitative Study  Key Processes:  Personal Reflexive process (conscientization) of the self from resistance narratives of masculinity, blackness, aggression to recovery narratives of family and connection.  Relational: practitioner encouraged dialogue and exploration.  Project design fostered a sense of ownership.  Particular activities e.g. creative writing fostered dialogue and reflection. |
| **Study Setting** | Her Majesty’s Prison Youth OffendingInstitution (HMPYOI) Werrington (UK) |
| **Notes** |  |

#### Risk of bias table

| **Item** | **Authors' judgement** | **Support for judgement** |
| --- | --- | --- |
| Random sequence generation | Unclear risk |  |
| Allocation concealment | Unclear risk |  |
| Blinding of participants and practitioners | Unclear risk |  |
| Blinding of assessors | Unclear risk |  |
| Incomplete outcome data | Unclear risk |  |
| Selective outcome reporting | Unclear risk |  |
| Are the data free of carry-over effects? | Unclear risk |  |

### Daykin 2017

| **Methods** | Study Design: Qualitative  Data Collection: Participant observation, interviews and focus groups  Analysis/Theoretical Perspective: Thematic analysis guided by principles of analytic induction, using the constant comparison method, being alert to deviant cases and endeavouring to treat the data comprehensively. |
| --- | --- |
| **Participants** | N=118 young people (N=81 male and N=37 female) aged between 13 and 21 years (mean age of 16.64 years) took part in the intervention.  Interviews and focus groups were with the participants, musicians and staff (N=31). Follow-up interviews with five participants took place three to six months later. |
| **Interventions and comparisons (where applicable)** | Intervention: Music programme led by a national UK arts charity. Fifteen projects were delivered across eight youth justice sites. Each project was facilitated by two or three young professional musicians who typically provided weekly sessions of ninety minutes to three hours for four to ten participants over a six-week period.  Comparison: None - qualitative study |
| **Outcomes** | Outcomes: N/A Qualitative Study  Key Processes: role of professional musicians in creating structure and order, building positive relationships and active learning, balance self-expression and responsiveness to time, managing group dynamics, cultural relevance, challenges in existing relationships with staff/other participants and when staff do not value the music project. |
| **Study Setting** | Youth justice setting (UK) |
| **Notes** |  |

#### Risk of bias table

| **Item** | **Authors' judgement** | **Support for judgement** |
| --- | --- | --- |
| Random sequence generation | Unclear risk |  |
| Allocation concealment | Unclear risk |  |
| Blinding of participants and practitioners | Unclear risk |  |
| Blinding of assessors | Unclear risk |  |
| Incomplete outcome data | Unclear risk |  |
| Selective outcome reporting | Unclear risk |  |
| Are the data free of carry-over effects? | Unclear risk |  |

### de Roeper 2009

| **Methods** | Study Design: Qualitative  Data Collection: Ethnographic participant observation, informal interviews  Analysis/Theoretical Perspective: Reflective thematic analysis, community cultural development (CCD) approach |
| --- | --- |
| **Participants** | N=22 young people aged 14-15 years started programme, most had problematic family situations in which drug usage, violence, abuse and/or mental or physical illness were commonplace.  N=12 (equal gender representation) participated in the final performance, a one-hour show entitled Skool Warz |
| **Interventions and comparisons (where applicable)** | Intervention:  The Makin’ it Peachy Programme delivered in a local community centre during school hours for two full days each week over 5 months and culminating in a performance during a local youth arts festival. Creative activities, mostly based on hip-hop culture, including break and freestyle dancing, digital music and rapping, and spray painting. Tutors in all activities were present at every session and the 20 participants (roughly equal representation of both genders) chose activities.  Public performance at the end of the programme - Skool Warz - featured music and rap songs composed and sung by one group of participants and break-dancing and hip hop choreo- graphed and performed by another group, on a set designed and painted by a third group  N=8 chose to participate in the subsequent 1:1 programme. They worked individually with community arts workers to develop and undertake a programme of activities that would enable them to pursue their chosen creative activity. Some attended dance classes, others attended recording sessions and worked with music tutors, and one attended acting classes and was taken onto the books of an acting agency.  Comparison: None - qualitative study |
| **Outcomes** | Outcomes: N/A Qualitative Study  Key Processes:  Culturally relevant arts, sense of ownership by participants, need for continuity and sustainable delivery |
| **Study Setting** | Local community centre during school hours for two full days each week over 5 months (Australia) |
| **Notes** |  |

#### Risk of bias table

| **Item** | **Authors' judgement** | **Support for judgement** |
| --- | --- | --- |
| Random sequence generation | Unclear risk |  |
| Allocation concealment | Unclear risk |  |
| Blinding of participants and practitioners | Unclear risk |  |
| Blinding of assessors | Unclear risk |  |
| Incomplete outcome data | Unclear risk |  |
| Selective outcome reporting | Unclear risk |  |
| Are the data free of carry-over effects? | Unclear risk |  |

### DeCarlo 2004

| **Methods** | Non randomised comparative study |
| --- | --- |
| **Participants** | African American adolescents with a history of offending.  N=21  All male |
| **Interventions and comparisons (where applicable)** | Intervention group:  Using rap music to develop prosocial skills. 6 weeks, x2 weekly  Comparison group:  Traditional psychoeducational group therapy |
| **Outcomes** | RAP therapy assessment scale (RTAS) (multidimensional) |
| **Study Setting** | Classroom environment, urban midwestern American city. |
| **Notes** |  |

#### Risk of bias table

| **Item** | **Authors' judgement** | **Support for judgement** |
| --- | --- | --- |
| Random sequence generation | High risk | No detail provided but allocation probably not randomised. |
| Allocation concealment | High risk | No detail provided but allocation probably not concealed. |
| Blinding of participants and practitioners | High risk | Blinding of participants and practitioners not reported but highly unlikely |
| Blinding of assessors | Unclear risk | Blinding of assessors not reported |
| Incomplete outcome data | High risk | No information reported regarding attrition |
| Selective outcome reporting | Unclear risk | No protocol or registration record available |
| Are the data free of carry-over effects? | Unclear risk |  |

### Flores 2016

| **Methods** | Study Design: Qualitative - Collective case study design.  Data Collection: Focused observation of workshop video recordings. Focus group interviews.  Analysis/Theoretical Perspective: Thematic analysis. |
| --- | --- |
| **Participants** | Nine boys and seven girls aged 7– 12 years from a child and youth care centre who presented with the highest levels of depression, anxiety, anger and/or difficulties with social interaction. |
| **Interventions and comparisons (where applicable)** | Intervention: Four months of weekly group drumming sessions each 45 minutes long. Sessions were designed and facilitated by the primary researcher, who had a background in both music education and Gestalt play therapy. Earlier exercises were more structured and facilitator-led, whereas later activities depended upon an interactive participant structure allowing participants to develop a sense of joint ownership over the music created. Groups of participants worked towards preparing an item for the centre’s annual Christmas concert.  Comparison: None - qualitative study |
| **Outcomes** | Outcomes: N/A Qualitative Study  Key Processes: enjoyment, awareness of others, contact, positive interaction, teamwork skills and leadership skills; regulation of social interaction, limits and boundaries, appropriate permissiveness, social flexibility and inclusion. |
| **Study Setting** | Child and youth care centre (UK) |
| **Notes** |  |

#### Risk of bias table

| **Item** | **Authors' judgement** | **Support for judgement** |
| --- | --- | --- |
| Random sequence generation | Unclear risk |  |
| Allocation concealment | Unclear risk |  |
| Blinding of participants and practitioners | Unclear risk |  |
| Blinding of assessors | Unclear risk |  |
| Incomplete outcome data | Unclear risk |  |
| Selective outcome reporting | Unclear risk |  |
| Are the data free of carry-over effects? | Unclear risk |  |

### Fullchange 2018

| **Methods** | Study Design: Qualitative  Data Collection: Interviews and online survey  Analysis/Theoretical Perspective: Two researchers independently coded responses for themes met to refine themes. |
| --- | --- |
| **Participants** | N=7 near-college-age youths (17-18 years) at a boys’ probation camp. N=5 boys identified as Latino, N=1 as Black, and N=1 as American Indian and White  N=7 University students enrolled in The Odyssey Project (TOP) as part of a summer session course. |
| **Interventions and comparisons (where applicable)** | Intervention: The Odyssey Project is a theater intervention bringing teens at a local probation camp together with University undergraduate student peers, culminating in a public theater performance in downtown Santa Barbara of an adaptation of the Homeric epic poem The Odyssey.  Comparison: None - qualitative study |
| **Outcomes** | Outcomes: N/A Qualitative Study  Key Processes: creating a psychologically safe space, positive relationships/interactions |
| **Study Setting** | University of California, Santa Barbara (USA) |
| **Notes** |  |

#### Risk of bias table

| **Item** | **Authors' judgement** | **Support for judgement** |
| --- | --- | --- |
| Random sequence generation | Unclear risk |  |
| Allocation concealment | Unclear risk |  |
| Blinding of participants and practitioners | Unclear risk |  |
| Blinding of assessors | Unclear risk |  |
| Incomplete outcome data | Unclear risk |  |
| Selective outcome reporting | Unclear risk |  |
| Are the data free of carry-over effects? | Unclear risk |  |

### Gann 2010

| **Methods** | Study design: Multimethod and mixed methods- Qualitative component (Grey literature-PhD thesis)  Data collection: Video taped interviews and a series of qualitative observations  Analysis/Theoretical perspective: Informed by pscyholaltyic thoereies and thematic analysis.  Deductive and inductive coding strategies. In addition, content analysis was used to determine what practices or factors would be the most useful for social work |
| --- | --- |
| **Participants** | Students were at-risk urban adolescents and were referred through the Berkeley High School health center  Student participants, all from lower socioeconomic backgrounds, consisted of three African-American participants and two Caucasian participants, ranging in age from 15-18 years |
| **Interventions and comparisons (where applicable)** | Intervention: *The Beats, Rhymes and Life Program (BLR)*  Drawing from Rap Therapy, Hip Hop Therapy, Group Therapy and Group Work frameworks, the BRL program serves as a therapeutic Hip Hop activity group which aims to create opportunities for creative expression, positive peer interaction and skills development for its youth participants.  The program took place over a six-week period, during which groups met once a week after school, for two hours at a time.  Comparison: None-qualitative study |
| **Outcomes** | Outcomes: N/A Qualitative Study  Key Processes: Individual development of positive attitude, communication skills, self confidence, coping, developing sense of self.  Cultural relevance/connection to hip hop  Group approach central to group functioning and individual development |
| **Study Setting** | Students from Oakland and Berkley high school groups (USA) |
| **Notes** |  |

#### Risk of bias table

| **Item** | **Authors' judgement** | **Support for judgement** |
| --- | --- | --- |
| Random sequence generation | Unclear risk |  |
| Allocation concealment | Unclear risk |  |
| Blinding of participants and practitioners | Unclear risk |  |
| Blinding of assessors | Unclear risk |  |
| Incomplete outcome data | Unclear risk |  |
| Selective outcome reporting | Unclear risk |  |
| Are the data free of carry-over effects? | Unclear risk |  |

### Gowland-Pryde 2017

| **Methods** | Study Design: QUAL  Semi structured interviews with young people and staff  5 sessions observations  Artist- Educator reflexive journals  Associated visual data  Analysis: Thematic analysis following Dillon typology structured around the four themes/areas of the typology: Buildings/Places, People/Bodies, Photograph/Artworks and Objects/Things |
| --- | --- |
| **Participants** | 6 YP, 3 in each of the two strands:  Strand 1: 2 male, 1 female  Strand 2: 2 male, 1 female . Ten young people, aged twelve to sixteen participated in the programme. Eight male and two female.5 had experienced custody on one or more occasion, one waiting to start first custodial sentence.3 from ethnically diverse backgrounds  9 staff - Artist educators, Youth Offending Service Workers:  Strand 1: 2 artist educators, 1 youth offending worker, 1 youth arts co-ordinator  Strand 2: 3 artist educators, 4 youth offending service |
| **Interventions and comparisons (where applicable)** | Gallery-supported arts awards programme delivered in partnership with the Wessex Youth Offending Team to support young people who have offended.  Strand 1: Summer Arts Colleges/Arts Award programme  Strand 2: weekly programme scheduled over nine months in 2012/2013 |
| **Outcomes** | Outcomes N/A QUAL study  Key processes:  Strand 1  Peer to peer relationships  Enjoyment  Strand 2  Longer term and consistent programme  Personalised learning approach  Motivation of an award  Supportive, non judgemental environment  New and unique activities |
| **Study Setting** | Art programme delivered at an art Gallery in Wessex, UK. |
| **Notes** |  |

#### Risk of bias table

| **Item** | **Authors' judgement** | **Support for judgement** |
| --- | --- | --- |
| Random sequence generation | Unclear risk |  |
| Allocation concealment | Unclear risk |  |
| Blinding of participants and practitioners | Unclear risk |  |
| Blinding of assessors | Unclear risk |  |
| Incomplete outcome data | Unclear risk |  |
| Selective outcome reporting | Unclear risk |  |
| Are the data free of carry-over effects? | Unclear risk |  |

### Hadland 2010

| **Methods** | QUAL phenomenology  Methods: Unstructured interviews  Analysis: Each sentence containing significant statements recorded on index cards. Repeated or overlapping statements written on the same index cards. Similar cards were grouped together under similar themes, which were then collated into broader themes. This process was repeated until two main themes were identified |
| --- | --- |
| **Participants** | 4 young people who participated in the community art project and permantely excluded from school-two males, aged 15 and 16 years, and two females, aged 14 and 15 years old. |
| **Interventions and comparisons (where applicable)** | Community art project delivered by City Arts Nottingham in an inner city area.  Engages participants in a series of creative activities to give them a sense of belonging and offer opportunities for self-expression. |
| **Outcomes** | Outcomes N/A QUAL  Key processes:  Sense of escape, freedom, emphasising achievment, new activities, inclusivity, peer relationships |
| **Study Setting** | Community art project delivered in inner city Nottingham |
| **Notes** |  |

#### Risk of bias table

| **Item** | **Authors' judgement** | **Support for judgement** |
| --- | --- | --- |
| Random sequence generation | Unclear risk |  |
| Allocation concealment | Unclear risk |  |
| Blinding of participants and practitioners | Unclear risk |  |
| Blinding of assessors | Unclear risk |  |
| Incomplete outcome data | Unclear risk |  |
| Selective outcome reporting | Unclear risk |  |
| Are the data free of carry-over effects? | Unclear risk |  |

### Hanrahan 2017

| **Methods** | Study Design: Qualitative  Data collection: Longitudinal design; in-depth individual interviews. Each participant was interviewed by the first author at three time points over 22 months  Analysis/Theoretical Perspective: IPA (Smith et al. 2009). The analysis process involved a number of stages which were adapted from Smith, Flowers, and Larkin(2009) and Smith and Osborn (2007) for longitudinal analysis. |
| --- | --- |
| **Participants** | 4 young people (15–21 years of age; M = 18.25, SD = 2.75) who had experienced school exclusion and additional challenging life experiences, such as unstable home environments, poverty, domestic violence, substance misuse, and involvement with the criminal justice system.  Three females; one male. All British with a mixed ethnic profile: two were mixed race, two were Black.  All pts had some amateur experience of taking part in drama and theatre workshops previously |
| **Interventions and comparisons (where applicable)** | Intervention: Drama and theatre project for young people run by a charitable theatre company with the aim to create a theatre production based on the life experiences of marginalised young people, with parts acted by the young people.  6-month period of weekly or biweekly drama workshops. then 12-week phase of intense rehearsing of a newly scripted version of the production. This work culminated in a 3-week run of the production  Comparison: None-Qualitative study |
| **Outcomes** | Outcomes: N/A Qualitative Study  Key Processes: Self expression, Self exploration. Potential of change behaviour and attitudes  Nurturing trust, support, encouragement, and belonging  Clear boundaries for positive relationships |
| **Study Setting** | Drama and theatre project for marginalised young people, run by charity (England) |
| **Notes** |  |

#### Risk of bias table

| **Item** | **Authors' judgement** | **Support for judgement** |
| --- | --- | --- |
| Random sequence generation | Unclear risk |  |
| Allocation concealment | Unclear risk |  |
| Blinding of participants and practitioners | Unclear risk |  |
| Blinding of assessors | Unclear risk |  |
| Incomplete outcome data | Unclear risk |  |
| Selective outcome reporting | Unclear risk |  |
| Are the data free of carry-over effects? | Unclear risk |  |

### Hickey 2018

| **Methods** | Study Design: Qualitative  Data Collection: Structured feedback sheets, Focus groups (N=12) and interviews with participants (N=4) and staff (N=3) over a 5 year period. Data also included field notes and reflective journaling by the project leader/evaluator.  Analysis/Theoretical Perspective: Computer assisted qualitative content analysis, Self-determination theory |
| --- | --- |
| **Participants** | N = 717 court-detained juveniles aged 13-18 years (average 15.8 years). |
| **Interventions and comparisons (where applicable)** | Intervention:  A music composition programme based mostly on rap music designed in accordance with self-determination theory, comprising up to 10 weekly sessions with 90 minutes of taught and self-directed project work using computer technology.  Comparison: None - qualitative study |
| **Outcomes** | Outcomes: N/A Qualitative Study  Key Processes:  Programme design offered creativity in contrast to controlled justice environment. This allowed for autonomy and relatedness.  Self-determination processes. Connection to music, learning something new, enjoyment and success.  Particular activities: no evidence regarding qualities of particular music genre. |
| **Study Setting** | Chicago, Cook County Juvenile Temporary Juvenile Detention Center (JTDC) (US) |
| **Notes** |  |

#### Risk of bias table

| **Item** | **Authors' judgement** | **Support for judgement** |
| --- | --- | --- |
| Random sequence generation | Unclear risk |  |
| Allocation concealment | Unclear risk |  |
| Blinding of participants and practitioners | Unclear risk |  |
| Blinding of assessors | Unclear risk |  |
| Incomplete outcome data | Unclear risk |  |
| Selective outcome reporting | Unclear risk |  |
| Are the data free of carry-over effects? | Unclear risk |  |

### Howard 2022

| **Methods** | Study Design: Qualitative  Data Collection: Multisite ethnography, participant observation, one-to-one interviews with participants and staff, and video recording.  Analysis/Theoretical Perspective: Grounded theory analysis |
| --- | --- |
| **Participants** | N = 46 aged between 14 and 23, categorised as ‘at-risk’ by reasons of school exclusion, behaviour, disability family and home environment. |
| **Interventions and comparisons (where applicable)** | Intervention:  The Arts Award is an accredited art learning framework for young people who are unlikely to engage in the arts or benefit from existing cultural provision.   The study covered various arts activities (dance, music, visual arts, and digital media)   Young people can gain an award by working with artists and arts professionals in a range of artforms including visual arts to music production, dance and drama.   Young people are required to create a portfolio by collecting ‘evidence’ of their journey and skills development. Attendances varies between being compulsory and open access.   50 different interviews were undertaken, but some participants had up to five interviews. 432 hours were spent undertaking fieldwork. Participants were also shadowed at community arts and performance events. Data include photos, artwork, drawings, music tracks, radio production and social media.  Comparison: None - qualitative study |
| **Outcomes** | Outcomes: N/A Qualitative Study  Key Processes:  Looks like this did not work? Because it did not give choice, cultural context and it was deficit orientated and didactic (these are process to avoid)  Programme design influenced by political agendas? Top down approach. |
| **Study Setting** | Three alternative education and two youth programmes based in the EastMidlands of England (UK) |
| **Notes** |  |

#### Risk of bias table

| **Item** | **Authors' judgement** | **Support for judgement** |
| --- | --- | --- |
| Random sequence generation | Unclear risk |  |
| Allocation concealment | Unclear risk |  |
| Blinding of participants and practitioners | Unclear risk |  |
| Blinding of assessors | Unclear risk |  |
| Incomplete outcome data | Unclear risk |  |
| Selective outcome reporting | Unclear risk |  |
| Are the data free of carry-over effects? | Unclear risk |  |

### Jordan 2015

| **Methods** | Study Design: Qualitative  Data Collection: Informal evaluation, In depth interviews (around 25-30 minutes) with diverted youth and parents  Analysis/Theoretical Perspective: NOT REPORTED |
| --- | --- |
| **Participants** | N = 21 participants in the programme  N = 14 interviewees were three males and 11 females aged 13-17.  N = 8 parents (seven mothers and one father) agreed to take part in a telephone interview |
| **Interventions and comparisons (where applicable)** | Intervention:  A University led Theatre in Diversion Programme, which offers prevention, education, accountability and treatment if appropriate, to juvenile offenders. Activities over 15 weeks are led by staff and students who receive academic credit.  Participants are diverted youth who attend 20 hours on campus, meeting three times a week over 10 weeks, to learn theatre skills, writing and staging one act plays in a final performance.   Comparison: None - qualitative study |
| **Outcomes** | Outcomes: N/A Qualitative Study  Key Processes:  Programme design was attractive to participants, hence creation of positive interaction |
| **Study Setting** | Western Kentucky University (US) |
| **Notes** |  |

#### Risk of bias table

| **Item** | **Authors' judgement** | **Support for judgement** |
| --- | --- | --- |
| Random sequence generation | Unclear risk |  |
| Allocation concealment | Unclear risk |  |
| Blinding of participants and practitioners | Unclear risk |  |
| Blinding of assessors | Unclear risk |  |
| Incomplete outcome data | Unclear risk |  |
| Selective outcome reporting | Unclear risk |  |
| Are the data free of carry-over effects? | Unclear risk |  |

### Lazzari 2005

| **Methods** | Study Design: Qualitative  Data Collection: Semi-structured interviews  Analysis/Theoretical Perspective: Grounded theory -conceptual categories and processes. |
| --- | --- |
| **Participants** | N = 31 were females aged 11 to 17 (average 15). They described themselves as multi-ethnic and cited reasons for incarceration including violation of parole or probation, failure to appear for court, and other offences including violence and theft. |
| **Interventions and comparisons (where applicable)** | Intervention:  A collaboration between a major regional art museum and a county juvenile detention centre in the western United States.  Regular art sessions aligned with education programming are led by a professional artist and involve the production of individual and collaborative paintings, sculptures, poems, and other artworks, which are displayed in public to draw attention to the incarceration of young women in the juvenile justice system.  The number of art sessions in which study participants participated ranged from 1 to 16.  Comparison: None - qualitative study |
| **Outcomes** | Outcomes: N/A Qualitative Study  Key Processes:  Building relationships, experiencing success and pride.  Practitioner attributes and behaviours. Role of the artist in creating environment that is trustworthy, caring, polite, stable, kind, nice, patient, and respectful.  Programme design allows based on trust relationships and recognises the value of exploration, expression (creativity) and achievement. |
| **Study Setting** | A county juvenile detention center in the western United States (US) |
| **Notes** |  |

#### Risk of bias table

| **Item** | **Authors' judgement** | **Support for judgement** |
| --- | --- | --- |
| Random sequence generation | Unclear risk |  |
| Allocation concealment | Unclear risk |  |
| Blinding of participants and practitioners | Unclear risk |  |
| Blinding of assessors | Unclear risk |  |
| Incomplete outcome data | Unclear risk |  |
| Selective outcome reporting | Unclear risk |  |
| Are the data free of carry-over effects? | Unclear risk |  |

### Lea 2019

| **Methods** | Study design: embedded qualitative case study. Data collection: Participant observation and semi structured interviews over 12 months (2016-2017). Data analysis: Thematic analysis of observational field notes and transcribed interview data assisted by computer software. The procedure included coding (initial, focused, axial), constant comparisons; and memoing. To ensure rigor and trustworthiness, several strategies were used including prolonged engagement, triangulation, peer debriefing, audit trail, reflexivity, and thick descriptions. |
| --- | --- |
| **Participants** | 117 students were enrolled during the study period including 66.7% males. 53.8% described themselves as African American/Black while 42.7% described themselves as Hispanic/Latino. The majority had previous criminal justice involvement and over half reported difficulties with education. Traumatic life events were also reported. Observed sessions typically included 14 students, five formerly incarcerated young Black men and five school personnel. Interviews were with incarcerated emerging adult Black men aged 18-25 (n=8) and school personnel (n=3). Participants reported a range of living situations: one was homeless, four were unemployed at the time of their first interview, and three reported current involvement with a gang. |
| **Interventions and comparisons (where applicable)** | The VOICES programme, a writing, poetry, and music program offered to incarcerated youth that uses a project-based learning curriculum where the emphasis is on expression. Students are taught the structure of poetry and learn how to write their own poems, create musical “beats,” and record and perform their poetry to the music created.  Comparisons not applicable (qualitative study). |
| **Outcomes** | Not applicable, qualitative study.  Processes  Culturally relevant curriculum. Creative activities reportedly helped to motivate and engage participants, aligning with their identities and strengths, offering meaningful activity and fostering a sense of belonging, mastery and safety.  Self-expression and reflection helped participants cope, gain peace and self-awareness, to build caring relationships and positive connections.  Programme design and practitioners' approaches foster trust, reflection and care. |
| **Study Setting** | An alternative school in which arts are viewed as a vehicle for promoting literacy, emotional skills, self-confidence and empowerment on order to foster positive life choices (US). |
| **Notes** |  |

#### Risk of bias table

| **Item** | **Authors' judgement** | **Support for judgement** |
| --- | --- | --- |
| Random sequence generation | Unclear risk |  |
| Allocation concealment | Unclear risk |  |
| Blinding of participants and practitioners | Unclear risk |  |
| Blinding of assessors | Unclear risk |  |
| Incomplete outcome data | Unclear risk |  |
| Selective outcome reporting | Unclear risk |  |
| Are the data free of carry-over effects? | Unclear risk |  |

### Lotter 2015

| **Methods** | Study Design: Qualitative  Data collection: Semi structured interview with 3 staff from the organization  Clinical session notes and video recorded excerpts from two music therapy sessions with 1 client  Analysis/Theoretical perspective: Miles & Huberman qual data analysis: 3 stages - segmentation, coding, organizing |
| --- | --- |
| **Participants** | 1 adolescents therapy client, Themba, a 17 years old school drop out, referred to YDO by the courts. Arrested for assault & referred to music therapy because of violent and aggressive tendencies.  3 staff from the organization: General Manager, Administrative and Arts Manager and thirdly the ADP Manager |
| **Interventions and comparisons (where applicable)** | Intervention: National Youth Development Outreach (YDO) in Eersterust, Pretoria who work w/ adolescents in conflict with the law, referred by the courts. YDO offers Adolescent Development Programme as a means of social rehabilitation.  13 individual music therapy sessions over 6 months included: drumming, pre-recorded music, improvisations on drum and piano, instrumental circles, guitar skills, song writing  Open group music therapy sessions. Weekly sessions of 30 minutes  Comparison: None-qualitative study |
| **Outcomes** | Outcomes: N/A Qualitative Study  Key Processes: Building sense of courage  Developing life skills  Taking responsibility  Self expression  Creative (musical) skill development  Communication  Sharing and helping  Cultural relevance connected to disadvantage  Social rehabilitation  Local community central to success  Ethos of rehabilitation within community |
| **Study Setting** | National Youth Development Outreach- Youth Development Programme (South Africa) |
| **Notes** |  |

#### Risk of bias table

| **Item** | **Authors' judgement** | **Support for judgement** |
| --- | --- | --- |
| Random sequence generation | Unclear risk |  |
| Allocation concealment | Unclear risk |  |
| Blinding of participants and practitioners | Unclear risk |  |
| Blinding of assessors | Unclear risk |  |
| Incomplete outcome data | Unclear risk |  |
| Selective outcome reporting | Unclear risk |  |
| Are the data free of carry-over effects? | Unclear risk |  |

### Massó-Guijarro 2020

| **Methods** | Study design: qualitative ethnography. Data collection: Participant observations, field notes, photographic and video recordings, informal conversations and open questionnaire. Details of analysis not provided. |
| --- | --- |
| **Participants** | 10 teenagers: males (8), the majority white except for 1 participant from the Roma community. |
| **Interventions and comparisons (where applicable)** | The initial intention to deliver a theatre based workshop was revised in favour of music (rap, dance, and the audio-visual media), which participants preferred. The intervention delivered by student facilitators. Participants were asked to write a song, eventually producing lyrics with themes of drug addiction and social prejudices about their group. A video clip was recorded to illustrate the passions of each teenager and objects chosen by participants were used to create a simple dramaturgy that gave structure and content to the video clip. Certain people preferred not to appear in the video but were able to collaborate in other tasks related to its production. Finally, an open exhibition of the video was organized for the family and friends of the participants. Despite the technical problems, the video was very well received and generated attitudes of excitement and surprise in adolescents. |
| **Outcomes** | None: Qualitative study.  Key Processes: The use of art forms preferred by young people, such as rap music, which denounces social injustices, was key to overcoming deficit labels and affirming the capacities of participants. The project was culturally relevant and, rather than being perceived as boring, stimilated a desire for self improvement among participants. Music allowed expressive possibilities of a vulnerable group, creating space for the analysis and reconstruction of the images and imagined futures that young people held about themselves. The audio-visual medium fostered cooperation, allowing social links to be established and renewed. The facilitators were close in age to the participants and therefore relatable. They were able to foster trust, critical thinking and reflection.  Comparison: none, qualitative study. |
| **Study Setting** | A compliance centre in a city in Northern Spain offering a social education project using arts to address drug misuse. The project is delivered over two months, with attendance compulsory as part of a requirement to fulfil legal measures. |
| **Notes** |  |

#### Risk of bias table

| **Item** | **Authors' judgement** | **Support for judgement** |
| --- | --- | --- |
| Random sequence generation | Unclear risk |  |
| Allocation concealment | Unclear risk |  |
| Blinding of participants and practitioners | Unclear risk |  |
| Blinding of assessors | Unclear risk |  |
| Incomplete outcome data | Unclear risk |  |
| Selective outcome reporting | Unclear risk |  |
| Are the data free of carry-over effects? | Unclear risk |  |

### Morgan 2020

| **Methods** | Qualitative. Data collection: Observations, focus groups and semi-structured interviews. Data analysis: Grounded theory with open, axial and selective coding and a four stage analysis process to draw out generic themes. |
| --- | --- |
| **Participants** | Interviews were undertaken with 12 participants drawn from a related survey of 74 participants aged 13-25. Just over half of survey participants described themselves as White males, and almost a third were living with their parent/s. Just over a third said they had been in trouble with the police and a small number had spent time in custody. In addition, interviews were undertaken with stakeholders/partners (N=3), and the project lead. A focus group was undertaken with the project team (N=6). |
| **Interventions and comparisons (where applicable)** | Intervention.  Participants choose between a variety of variety of sports, media and arts activities designed to progress them into employment, education and/or training. Future Stars works with corporate/business partners and statutory agencies and utilises the expertise of highly skilled and well-respected individuals from across the sports, arts, entertainments, education and youth and community sectors. Arts interventions are not described in detail.  Comparison: None - qualitative study. |
| **Outcomes** | Outcomes: N/A - qualitative study.  Processes  Recognition and acceptance as a foundation for social inclusion emerged as a key theme. The project helped young people to overcome barriers by fostering strong interpersonal relationships, through which young people could be recognised for the qualities they brought to the project.Informal rather than formal interactions between leaders and participants allowed a sense of acceptance and trust to develop. The project provided access to social capital including practical employment support and development opportunities. |
| **Study Setting** | A UK based charitable organisation catering for young people aged 11-25 years, many of whom are within the care system or involved (or termed ‘at-risk’ of involvement) in youth and/or violent gang-related crime. |
| **Notes** |  |

#### Risk of bias table

| **Item** | **Authors' judgement** | **Support for judgement** |
| --- | --- | --- |
| Random sequence generation | Unclear risk |  |
| Allocation concealment | Unclear risk |  |
| Blinding of participants and practitioners | Unclear risk |  |
| Blinding of assessors | Unclear risk |  |
| Incomplete outcome data | Unclear risk |  |
| Selective outcome reporting | Unclear risk |  |
| Are the data free of carry-over effects? | Unclear risk |  |

### Nicklin 2017

| **Methods** | Qualitative. Data collection: Participant observations in 12 sessions during 2015, interviews and feedback. Data analysis: Thematic analysis of excerpts of observation data from research diaries and feedback. |
| --- | --- |
| **Participants** | 56 participants were involved in feedback while participant observation was undertaken with 6 participants. Participants are all involved in non-violent, anti-social, negative behavioural or criminal action and all living within an area of low socioeconomic status. |
| **Interventions and comparisons (where applicable)** | Intervention:  A Shakespeare-based alternative after-school education programme using games and activities to explore issues, build skills, and enhance confidence across 10 weeks. Every workshop is bookended by a ‘check-in’ and ‘reinforcement’ session. Attendance is compulsory.  Comparison: None- qualitative study. |
| **Outcomes** | Outcomes: N/A - qualitative study.  Processes  The programme allows participants live normal lives, with minimal disruption to school attendance. Activities such as compulsory check ins, while initially resisted, eventually encourage reflection and critical thinking. Shakespearean works include an extensive character range that are widely relatable. All participant contributions and ideas are valued by facilitators. The activities require collective working, allowing supportive relationships of trust and collective commitment to shared standards of behaviour. Participants are required to make decisions and interpret complex texts with no use of the internet. This fostered independent thought and pride in having succesfully engaged with material that is perceived as difficult. |
| **Study Setting** | An alternative after-school education programme for young people within the criminal justice system, US. |
| **Notes** |  |

#### Risk of bias table

| **Item** | **Authors' judgement** | **Support for judgement** |
| --- | --- | --- |
| Random sequence generation | Unclear risk |  |
| Allocation concealment | Unclear risk |  |
| Blinding of participants and practitioners | Unclear risk |  |
| Blinding of assessors | Unclear risk |  |
| Incomplete outcome data | Unclear risk |  |
| Selective outcome reporting | Unclear risk |  |
| Are the data free of carry-over effects? | Unclear risk |  |

### Parker 2018

| **Methods** | Qualitative. Data collection: Interviews of 15 - 30 min and were audio recorded and transcribed in full. Data analysis: Thematic and axial coding were used to analyze the data in four stages. |
| --- | --- |
| **Participants** | 32 pupils (28 male and 4 female) aged between 13 and 16 years participated in the intervention, some of whom were considered "at risk." |
| **Interventions and comparisons (where applicable)** | Intervention:  A school-based mentoring intervention for pupils engaged in, or at risk of, delinquency both within and outside of the school environment. A 10-week music-based program supported by tutors and provided by mental health charity. Sessions involved lyric writing, composing beats, recording, and/or performing music.  Comparison  None- qualitative study. |
| **Outcomes** | Outcomes: N/A - qualitative study.  Processes  The use of music afforded a sense of escapism and relaxation, participants seemed strongly motivated to attend. Participants were able to express positive and negative feelings and receive positive feedback and praise from others. The project fostered self reflection, examining beliefs and attitudes. Facilitators listened to participants and showing interest in them, building positive relationships through mentoring based on respect and one to one support. |
| **Study Setting** | A UK based school based mentoring programme for young people 'at risk'. |
| **Notes** |  |

#### Risk of bias table

| **Item** | **Authors' judgement** | **Support for judgement** |
| --- | --- | --- |
| Random sequence generation | Unclear risk |  |
| Allocation concealment | Unclear risk |  |
| Blinding of participants and practitioners | Unclear risk |  |
| Blinding of assessors | Unclear risk |  |
| Incomplete outcome data | Unclear risk |  |
| Selective outcome reporting | Unclear risk |  |
| Are the data free of carry-over effects? | Unclear risk |  |

### Podkalicka 2009

| **Methods** | Study Design: Qualitative  Data Collection: Ethnographic Observations  Analysis/Theoretical Perspective: Analysis of reflexive listening moments, model of social listening and social change in a broader sense of deliberative democracy. |
| --- | --- |
| **Participants** | ‘Youth at risk’ - young people who are either in residential or foster care, long-term disengaged from mainstream education, with a low literacy level, and/or drug, alcohol or juvenile justice issues. N=100 taking part at YouthWorx media programme recuriting social agencies. |
| **Interventions and comparisons (where applicable)** | Intervention:  Young people are encouraged and supported to express themselves by cocreating personal digital stories, recording original songs, producing artworks and radio content. Produce their own live-to-air radio program.  Comparison: None - Qualitative study |
| **Outcomes** | Outcomes: N/A Qualitative Study  Key Processes:  Reflexive process of ‘listening to oneself’, collaborative listening to others, empowerment and responsibility of being listened to |
| **Study Setting** | Community/social organisation - YouthWorx, an open access and independent media program (Australia) |
| **Notes** |  |

#### Risk of bias table

| **Item** | **Authors' judgement** | **Support for judgement** |
| --- | --- | --- |
| Random sequence generation | Unclear risk |  |
| Allocation concealment | Unclear risk |  |
| Blinding of participants and practitioners | Unclear risk |  |
| Blinding of assessors | Unclear risk |  |
| Incomplete outcome data | Unclear risk |  |
| Selective outcome reporting | Unclear risk |  |
| Are the data free of carry-over effects? | Unclear risk |  |

### Pope 2022

| **Methods** | Study Design: Qualitative  Data Collection: Ethnographic observations and interpretive interviews (20 months)  Analysis/Theoretical Perspective: Thematic analysis, informed by interpretive interactionism to examine the lived experience of participants. |
| --- | --- |
| **Participants** | Young adult participants involved in non-violent crime, informal conversations N=20; formal interviews N=12 |
| **Interventions and comparisons (where applicable)** | Intervention:  Spoken word as a modality to document and perform stories of struggle and overcoming challenges. Stories shared prior to court involvement to describe imagined futures. Utilized two creative arts experts from the community to facilitate programming through local non-profit spoken-word organization. Programming nights involved the facilitators engaging participants in a group discussion by introducing a topic or model poem. Following group discussion, young adults participated in writing assignments, which allowed them to reflect on the chosen topic. Then had the option to share insights with the group, and perform short written works  Comparison: None - qualitative study |
| **Outcomes** | Outcomes: N/A Qualitative Study  Key Processes:  Owndership/empowerment of voice, caring/sharing staff, self expression/managing emotion, expert facilitation. |
| **Study Setting** | Young adult problem-solving court (USA) |
| **Notes** |  |

#### Risk of bias table

| **Item** | **Authors' judgement** | **Support for judgement** |
| --- | --- | --- |
| Random sequence generation | Unclear risk |  |
| Allocation concealment | Unclear risk |  |
| Blinding of participants and practitioners | Unclear risk |  |
| Blinding of assessors | Unclear risk |  |
| Incomplete outcome data | Unclear risk |  |
| Selective outcome reporting | Unclear risk |  |
| Are the data free of carry-over effects? | Unclear risk |  |

### Ruggiero 2013

| **Methods** | Study Design: Qualitative  Data Collection: Interviews and journaling  Analysis/Theoretical Perspective: Inductive textual analysis and content analysis, cooperative learning model of video games development |
| --- | --- |
| **Participants** | Juvenile youth offenders |
| **Interventions and comparisons (where applicable)** | Intervention:  Research-based educational pilot program to teach socially responsible serious game development. Learners attended a two-week game camp and then attended bi-weekly design sessions for 12 months. The learning procedures include: (a) self-led learning, in which students and educators learn individually through their own independent game development process, (b) peer-to-peer learning, in which learners work with each other on game creation, and (c) expert-guided learning, in which graduate students help scaffold learning and solve problems on demand  Comparison: None - qualitative study |
| **Outcomes** | Outcomes: N/A Qualitative Study  Key Processes:  Self-led learning, creative immersion and discovery, interactive relationships, meaning making. |
| **Study Setting** | Video game development camp (USA) |
| **Notes** |  |

#### Risk of bias table

| **Item** | **Authors' judgement** | **Support for judgement** |
| --- | --- | --- |
| Random sequence generation | Unclear risk |  |
| Allocation concealment | Unclear risk |  |
| Blinding of participants and practitioners | Unclear risk |  |
| Blinding of assessors | Unclear risk |  |
| Incomplete outcome data | Unclear risk |  |
| Selective outcome reporting | Unclear risk |  |
| Are the data free of carry-over effects? | Unclear risk |  |

### Seroczynski 2011

| **Methods** | Study Design: Qualitative (Mixed Methods)  Data Collection: Reading, journaling, discussion  Analysis/Theoretical Perspective: Thematic analysis, |
| --- | --- |
| **Participants** | N= 29 participants were in seventh, eighth, and ninth grade moderate offenders who selected for the JJC’s Central Academy - academic day-treatment program, based on their potential for social, emotional, and academic success.  N=22 male (75.9%) and N=7 female participants (24.1%) . Average age 16.5 years (SD= 1.0); the youngest was 14 and the oldest 18 years. Participants’ mean family size was 4.69people (SD= 1.42; range = 2-7). Most lived with either biological mother alone with siblings(24.1%;N= 7) or biological mother and stepfather or boyfriend and siblings (27.6%;n= 8);13.8% (N= 4) added grandparents to the previous constellation; 10.3% (N= 3) lived with both biological parents, 13.8% (N= 4) lived with other relatives (e.g., grandparent, aunt), and6.9% (N= 2) lived with foster parents. Median family income was $16,050 (range = $0 to 58,000). 41.4% (N= 12) Caucasian American, 37.9% (N= 11) African American,6.9% (N= 2); Hispanic American, and13.8% (N= 4) multiracial. Offense for admission into Central Academy included burglary/theft (27.6%;N= 8), battery (24.1%;N=7), truancy (20.7%;N= 6), and running away(6.9%;n= 2). N=6 others (20.7%) were admit-ted under a variety of offenses including false informing and resisting law enforcement. Some students also had drug convictions. |
| **Interventions and comparisons (where applicable)** | Intervention:  Reading aloud, journaling and discussion of Harry Potter novels as a way to foster character development. Undergraduates facilitated a weekly “story hour”. After about two weeks, participants began to volunteer for “parts”. Each week participants were given five to seven questions to think and write about in a spiral-bound journal. Questions were designed to address moral and psychological issues found in the novels. Both principal investigators met with the students 90 minutes/week to discuss the chapters, review student responses to questions, and engage in hands-on activities designed to facilitate more thematic discussions.  Comparison: N/A for qualitative com |
| **Outcomes** | Outcomes: N/A for qualiative component  Key Processes:  Building positive relationships, fostering imagination regarding future self and compassion for others, expert practioner behaviours |
| **Study Setting** | Academic day-treatment programme, education setting. |
| **Notes** |  |

#### Risk of bias table

| **Item** | **Authors' judgement** | **Support for judgement** |
| --- | --- | --- |
| Random sequence generation | Unclear risk |  |
| Allocation concealment | Unclear risk |  |
| Blinding of participants and practitioners | Unclear risk |  |
| Blinding of assessors | Unclear risk |  |
| Incomplete outcome data | Unclear risk |  |
| Selective outcome reporting | Unclear risk |  |
| Are the data free of carry-over effects? | Unclear risk |  |

### Tett 2012

| **Methods** | Study Design: Qualitative  Data Collection: Qualitative Focus Groups (before and after intervention)  Analysis/Theoretical Perspective: Thematic Analysis |
| --- | --- |
| **Participants** | Project initially involved 25 young men who worked alongside the Scottish Ensemble’s Artist in Residence and the music tutor at the Young Offenders Institute.  15 prisoners (young offender’s institution) performed with members of the Scottish Ensemble and a second performance with the entire Scottish Ensemble. |
| **Interventions and comparisons (where applicable)** | Intervention: Scottish Opera and the Scottish Chamber Orchestra, and the Citizens Theatre. The Scottish Ensemble’s Music for Change project initially involved 25 young men who worked alongside the Scottish Ensemble’s Artist in Residence and the music tutor at the Young Offenders Institute in learning how to play and record music over four months.  Comparison: None - qualitative study |
| **Outcomes** | Outcomes: N/A Qualitative Study  Key Processes: changing attitudes, creating culture of positive learning  Relationships, collaborative working, programme design emphasises creative arts rather than employment skills. Experiencing success, cultural competence and critical consciousness. Programme design emphasises cultural relevance, practitioner competencies regarding offender settings and culturally diverse music. |
| **Study Setting** | Young Offenders Insitute; Scotland UK |
| **Notes** |  |

#### Risk of bias table

| **Item** | **Authors' judgement** | **Support for judgement** |
| --- | --- | --- |
| Random sequence generation | Unclear risk |  |
| Allocation concealment | Unclear risk |  |
| Blinding of participants and practitioners | Unclear risk |  |
| Blinding of assessors | Unclear risk |  |
| Incomplete outcome data | Unclear risk |  |
| Selective outcome reporting | Unclear risk |  |
| Are the data free of carry-over effects? | Unclear risk |  |

### Thompson 2015

| **Methods** | Study Design: Qualitative  Data Collection: Analytical Autoethnography  Analysis/Theoretical Perspective: Autoethnography analysed in terms of three pillars for culturally relevant pedagogies as (1) experiences that help students achieve academic success, (2) experiences that validate students’ cultural competence, and (3) experiences that assist students in developing critical consciousness. |
| --- | --- |
| **Participants** | Author was graduate assistant for a music composition project with incarcerated youth focus on teaching more creative aspects of music making and music composition.  Participants were in two groups. The first group was in temporary detention, having committed minor offences. As a result, students in this group were highly transient, often detained from two days to two weeks or more. Those in the second group were called ‘Automatic Transfers’ because they had committed more egregious crimes and were awaiting transfer to a more permanent facility when they turned 17 years old and would no longer be considered juveniles. |
| **Interventions and comparisons (where applicable)** | Intervention: a music composition project with incarcerated youth focus on teaching more creative aspects of music making and music composition.  Comparison: N/A |
| **Outcomes** | Experiencing success, cultural competence and critical consciousness.  Programme design emphasises cultural relevance, practitioner competencies regarding offender settings and culturally diverse music. |
| **Study Setting** | USA, young adult prison |
| **Notes** |  |

#### Risk of bias table

| **Item** | **Authors' judgement** | **Support for judgement** |
| --- | --- | --- |
| Random sequence generation | Unclear risk |  |
| Allocation concealment | Unclear risk |  |
| Blinding of participants and practitioners | Unclear risk |  |
| Blinding of assessors | Unclear risk |  |
| Incomplete outcome data | Unclear risk |  |
| Selective outcome reporting | Unclear risk |  |
| Are the data free of carry-over effects? | Unclear risk |  |

### Thompson 2022

| **Methods** | Study Design: Qualitative  Data collection: Qualitative bounded intrinsic case study (16 months).  Individual interviews (10-20 minutes) with students and staff members, a focus group discussion, and observational field notes  Analysis/Theoretical Perspective: Grounded Theory |
| --- | --- |
| **Participants** | 8/22 eight students volunteered to participate in this study. None had prior experience learning a string instrument. 5 young women and 3 young men r 13-17 years. |
| **Interventions and comparisons (where applicable)** | Intervention: Assigned an available instrument if possible, the kind most interesting to them. Taught how to play violin, viola, and cello in weekly group classes, and the youth performed in a chamber ensemble. The group gave approximately seven concerts inside and five concerts outside the facility. Guest musicians came on concert day to help with concert logistics, interact with the youth, and perform as part of the ensemble.  Comparisons: N/A |
| **Outcomes** | Outcomes: N/A Qualitative Study  Key Processes: exposure to new experiences, enjoyment, opportunity.  Feelings of pride and recognition (positive feedback)  Personal development and expression, releasing emotion, and interpersonal development, developing teamwork and communication skills.  Programme designed to expose participants to musical forms that they hadn’t previously encountered. |
| **Study Setting** | USA, youth justice system |
| **Notes** |  |

#### Risk of bias table

| **Item** | **Authors' judgement** | **Support for judgement** |
| --- | --- | --- |
| Random sequence generation | Unclear risk |  |
| Allocation concealment | Unclear risk |  |
| Blinding of participants and practitioners | Unclear risk |  |
| Blinding of assessors | Unclear risk |  |
| Incomplete outcome data | Unclear risk |  |
| Selective outcome reporting | Unclear risk |  |
| Are the data free of carry-over effects? | Unclear risk |  |

### Tyson 2002

| **Methods** | Randomised controlled trial |
| --- | --- |
| **Participants** | Runaway, abused, abandoned, neglected, truant, and youth who are otherwise homeless. N=14 |
| **Interventions and comparisons (where applicable)** | Hip-Hop therapy.  Historical teaching, listening to rap music and group discussion of relevant themes.  X3 weekly for 4 non-consecutive weeks |
| **Outcomes** | Self-concept  Peer relations |
| **Study Setting** | Miami Youth Services Center. Residential facility. |
| **Notes** |  |

#### Risk of bias table

| **Item** | **Authors' judgement** | **Support for judgement** |
| --- | --- | --- |
| Random sequence generation | Low risk | Randomisation by random numbers table |
| Allocation concealment | High risk | No concealment of allocation |
| Blinding of participants and practitioners | High risk | No blinding of participants and practitioners |
| Blinding of assessors | Unclear risk | No mention of blinding of assessors |
| Incomplete outcome data | High risk | 3/14 (21%) lost to follow-up. Analysis excludes those participants. |
| Selective outcome reporting | Unclear risk | No protocol or registration record available. |
| Are the data free of carry-over effects? | Unclear risk |  |

### Varley 2019

| **Methods** | Study Design: Qualitative (Grey literature; PhD Thesis)  Data Collection: Qualitative semi-structured interviews were undertaken with a sample of 10 offenders, asking them for their views on the V2 intervention programme at the three months follow-up stage.  Analysis/Theoretical Perspective: Thematic Analysis |
| --- | --- |
| **Participants** | For the interviews n=10 post interventions male young offenders aged 10-18 years of age (mean age 15 years, SD = 1.66), referred by Birmingham Youth Offending Service over a period of 18 months, to the Recre8 company, which used the V2 method of drama-based intervention. |
| **Interventions and comparisons (where applicable)** | Intervention: Drama Intervention based on RECRE8. The Recre8 interventions address previous antisocial behaviour; antisocial cognition through the use of a central character(s); influence of peers; positive relationships; encouraging educational performance and leisure activities by encouraging and enhance involvement and encourage participation through drama techniques, with an aim to reduce the risk of reoffending.  Comparison: N/A |
| **Outcomes** | Outcomes: N/A Qualitative Study  Key Processes: programme distinctiveness, Relatable content, safe for learning. Practitioner attributes: going above and beyond.  Commitment by supporters e.g. facilitators, peer mentors, familial structure in groups  Art form specific: drama techniques central (creativity/self expression)  Personal development, cognitive development, hope for the future. |
| **Study Setting** | UK; Young offenders serving community sentences and referred by Birmingam Youth Offending Service |
| **Notes** |  |

#### Risk of bias table

| **Item** | **Authors' judgement** | **Support for judgement** |
| --- | --- | --- |
| Random sequence generation | Unclear risk |  |
| Allocation concealment | Unclear risk |  |
| Blinding of participants and practitioners | Unclear risk |  |
| Blinding of assessors | Unclear risk |  |
| Incomplete outcome data | Unclear risk |  |
| Selective outcome reporting | Unclear risk |  |
| Are the data free of carry-over effects? | Unclear risk |  |

### Winn 2010

| **Methods** | Study Design: Qualitative  Data collection: Three-year multi-sited ethnography journeying through regional youth detention centers (RYDCs). Summer programme interviews with formerly incarcerated girls about the role of playwriting and performance in their lives as well as the tools they use from these experiences.  Analysis/Theoretical perspective: No detail |
| --- | --- |
| **Participants** | Female Black incarcerated or formerly incarcerated youth |
| **Interventions and comparisons (where applicable)** | Intervention: Girl Time conducts two-day playwriting workshops in the RYDCs in which girls learn how to build-ensemble, write plays, and put a play on. After two-day workshop, girls perform these plays written by their peers in front of other incarcerated youth, junior correctional officers RYDC administrators, and their families who all participate in a community dialogue about the plays, characters, themes, and issues around incarceration or related topics. During the summer, Girl Time invites formerly incarcerated girls to prepare a summer performance in which 8–10 plays written in the detention centers are staged in a public theatre.  Comparisons: N/A |
| **Outcomes** | Outcomes: N/A Qualitative Study  Key Processes: Personal reflections, making sense of life, Confronting difficulty  Programme philosophy, Pedagogy of empowerment.  Political connections, citizenship. |
| **Study Setting** | USA; Regional Youth Detention Centres |
| **Notes** |  |

#### Risk of bias table

| **Item** | **Authors' judgement** | **Support for judgement** |
| --- | --- | --- |
| Random sequence generation | Unclear risk |  |
| Allocation concealment | Unclear risk |  |
| Blinding of participants and practitioners | Unclear risk |  |
| Blinding of assessors | Unclear risk |  |
| Incomplete outcome data | Unclear risk |  |
| Selective outcome reporting | Unclear risk |  |
| Are the data free of carry-over effects? | Unclear risk |  |

### Winn 2011

| **Methods** | Study Design: Qualitative  Data collection: multi site ethnography – findings from N=2 participants  Analysis/Theoretical perspective: No detail |
| --- | --- |
| **Participants** | Two Black females, formerly incarcerated engaged in Girl Time, a theater program orchestrated by a women-focused theater company in the urban southeast. Nia and Sanaa use theatre as a way to re-introduce themselves to the world. Performance is followed by a “talk back” in which boundaries between the audience and performers are blurred in order to create a forum to discuss issues that have impacted the lives of girls. Encouraged to confront, heal, and transform the reality of past and current circumstances by telling their stories and the stories of their peers. |
| **Interventions and comparisons (where applicable)** | Intervention: Girl Time, a theater program orchestrated by a women-focused theater company in the urban southeast.Performance is followed by a “talk back” in which boundaries between the audience and performers are blurred in order to create a forum to discuss issues that have impacted the lives of girls. Encouraged to confront, heal, and transform the reality of past and current circumstances by telling their stories and the stories of their peers. |
| **Outcomes** | N/A Qualitative Study  Key Processes: Performance of possibility  Radical healing processes in story telling / re-telling story as possibility for future |
| **Study Setting** | USA; program for formerly incarcerated black girls seeking re-entry to their schools, communities, and families |
| **Notes** |  |

#### Risk of bias table

| **Item** | **Authors' judgement** | **Support for judgement** |
| --- | --- | --- |
| Random sequence generation | Unclear risk |  |
| Allocation concealment | Unclear risk |  |
| Blinding of participants and practitioners | Unclear risk |  |
| Blinding of assessors | Unclear risk |  |
| Incomplete outcome data | Unclear risk |  |
| Selective outcome reporting | Unclear risk |  |
| Are the data free of carry-over effects? | Unclear risk |  |

### Zlotowitz 2016

| **Methods** | Study Design: Qualitative  Data collection: Qualitative ethnography (field notes)  Analysis/Theoretical Perspective: Thematic Analysis to focus on perceptions of delivery |
| --- | --- |
| **Participants** | In an inner-city, high-density housing estate in the United Kingdom, with approximately 500 apartments. 60% of residents were white and 40% from black and ethnic minority groups. Area fell within the 14% most deprived similar-sized areas in England.  25 young people over 2 years. Intervention focused on a core group of 15 young people who were informally identified as the most in need. Of the core group, the majority were male (n = 13), two females. Most were of white British ethnicity, with a minority of Eastern European origin, Asian or black British. This pattern of ethnicity approximated that of the local community. Median participant age was 19; range 16–22. Exact demographic data were not known for all young people because they reported that such data collection was a barrier to attendance |
| **Interventions and comparisons (where applicable)** | Intervention: Coproduction of ‘Music and Change’ led by young people, it became centred on using contemporary music skills (e.g. DJ-ing and lyric writing) as a vehicle for building relationships and over time helping the young people in ways they requested, including supporting their mental health.  Comparisons: N/A |
| **Outcomes** | Outcomes: N/A for qualitative  Key processes: programme fosters trust - Trusted relationships (long-term and consistent, non-judgemental). Programme is responsive, flexible, relevant. Local and safe, non stigmatising. Peer and youth -led, holistic. Positive experiences, development of trusting relationships, overcoming barriers to help- seeking behaviours. |
| **Study Setting** | In an inner-city, high density housing estate in the UK |
| **Notes** |  |

#### Risk of bias table

| **Item** | **Authors' judgement** | **Support for judgement** |
| --- | --- | --- |
| Random sequence generation | Unclear risk |  |
| Allocation concealment | Unclear risk |  |
| Blinding of participants and practitioners | Unclear risk |  |
| Blinding of assessors | Unclear risk |  |
| Incomplete outcome data | Unclear risk |  |
| Selective outcome reporting | Unclear risk |  |
| Are the data free of carry-over effects? | Unclear risk |  |

###### Footnotes

## Characteristics of excluded studies

### Ajayi 2015

| **Reason for exclusion** | Wrong population |
| --- | --- |

### Aleksienė 2014

| **Reason for exclusion** | Not an evaluation of specific intervention |
| --- | --- |

### Amitay 2022

| **Reason for exclusion** | Population |
| --- | --- |

### Argyle 2005

| **Reason for exclusion** | Wrong population |
| --- | --- |

### Arroyo 2019

| **Reason for exclusion** | Wrong study design |
| --- | --- |

### Aventin 2019

| **Reason for exclusion** | Wrong population |
| --- | --- |

### Basto-Pereira 2015

| **Reason for exclusion** | Study Design (Review) |
| --- | --- |

### Beaulac 2010

| **Reason for exclusion** | Wrong population |
| --- | --- |

### Blau 2016

| **Reason for exclusion** | Intervention |
| --- | --- |

### Bornmann 2011

| **Reason for exclusion** | Wrong population |
| --- | --- |

### Bramwell 2018

| **Reason for exclusion** | Wrong population |
| --- | --- |

### Bravo 2022

| **Reason for exclusion** | Wrong study design |
| --- | --- |

### Brooks 2015

| **Reason for exclusion** | Wrong study design |
| --- | --- |

### Bulgren 2020

| **Reason for exclusion** | Wrong population |
| --- | --- |

### Caló 2019

| **Reason for exclusion** | Wrong population |
| --- | --- |

### Campbell 2019

| **Reason for exclusion** | Population |
| --- | --- |

### Carder 2007

| **Reason for exclusion** | Wrong study design |
| --- | --- |

### Carpenter 2018

| **Reason for exclusion** | Study design |
| --- | --- |

### Cleaver 2014

| **Reason for exclusion** | Population |
| --- | --- |

### Cursley 2019

| **Reason for exclusion** | Wrong population |
| --- | --- |

### Dalgaard 2022

| **Reason for exclusion** | Study design (review) & population |
| --- | --- |

### Davis 2020

| **Reason for exclusion** | Wrong study design |
| --- | --- |

### Daykin 2013

| **Reason for exclusion** | Wrong study design |
| --- | --- |

### Deuchar 2013

| **Reason for exclusion** | Wrong intervention |
| --- | --- |

### Dodsley 2021

| **Reason for exclusion** | Intervention |
| --- | --- |

### Edmunds 2018

| **Reason for exclusion** | Wrong study design |
| --- | --- |

### Evans-Chase 2014

| **Reason for exclusion** | Study Design (Review) |
| --- | --- |

### Everett 2022

| **Reason for exclusion** | Wrong study design |
| --- | --- |

### Gallagher 2017

| **Reason for exclusion** | Not an evaluation of a specific intervention |
| --- | --- |

### Geagea 2019

| **Reason for exclusion** | Population & outcome |
| --- | --- |

### Harding 1996

| **Reason for exclusion** | Not an evaluation of a specific intervention |
| --- | --- |

### Harkins 2011

| **Reason for exclusion** | Wrong population |
| --- | --- |

### Harkins 2016

| **Reason for exclusion** | Population |
| --- | --- |

### Harris 2012

| **Reason for exclusion** | Wrong population |
| --- | --- |

### Harris 2014

| **Reason for exclusion** | Intervention |
| --- | --- |

### Head 2015

| **Reason for exclusion** | Not an evaluation of a specific intervention |
| --- | --- |

### Henley 2015

| **Reason for exclusion** | Study design |
| --- | --- |

### Hess 2019

| **Reason for exclusion** | Not an evaluation of a specific intervention |
| --- | --- |

### Hon 2021

| **Reason for exclusion** | Population |
| --- | --- |

### Hoogsteder 2018

| **Reason for exclusion** | Wrong comparator |
| --- | --- |

### Kallio 2016

| **Reason for exclusion** | Wrong population |
| --- | --- |

### Kallio 2022

| **Reason for exclusion** | Wrong study design |
| --- | --- |

### Kim 2015

| **Reason for exclusion** | Wrong population |
| --- | --- |

### La Porte 2016

| **Reason for exclusion** | Population |
| --- | --- |

### Lai 2020

| **Reason for exclusion** | Study design/intervention |
| --- | --- |

### Lev-Aladgem 2010

| **Reason for exclusion** | Wrong population |
| --- | --- |

### Logie  2021

| **Reason for exclusion** | Wrong population |
| --- | --- |

### McCann 1996

| **Reason for exclusion** | Not an evaluation of a specific intervention |
| --- | --- |

### McCrary 2019

| **Reason for exclusion** | Review & intervention |
| --- | --- |

### McCray 2017

| **Reason for exclusion** | Wrong intervention |
| --- | --- |

### Meekums 2011

| **Reason for exclusion** | Wrong study design |
| --- | --- |

### Milner 2000

| **Reason for exclusion** | Wrong study design |
| --- | --- |

### Miner-Romanoff 2016

| **Reason for exclusion** | Intervention |
| --- | --- |

### Mohler 2012

| **Reason for exclusion** | Wrong study design |
| --- | --- |

### Morehouse 2000

| **Reason for exclusion** | Wrong intervention |
| --- | --- |

### Moyer 2020

| **Reason for exclusion** | Not an evaluation of a specific intervention |
| --- | --- |

### Mukherjee 2021

| **Reason for exclusion** | Wrong population |
| --- | --- |

### Nelson 2012

| **Reason for exclusion** | Wrong population |
| --- | --- |

### Nind 2012

| **Reason for exclusion** | Wrong population |
| --- | --- |

### Oesterreich 2009

| **Reason for exclusion** | Study design |
| --- | --- |

### Oosthuizen 2019

| **Reason for exclusion** | Intervention |
| --- | --- |

### Pane 2015

| **Reason for exclusion** | Study design |
| --- | --- |

### Payne 2021

| **Reason for exclusion** | Intervention/population |
| --- | --- |

### Pyles 2017

| **Reason for exclusion** | Population |
| --- | --- |

### Qiu 2016

| **Reason for exclusion** | Wrong population |
| --- | --- |

### Rapp-Paglicci 2009

| **Reason for exclusion** | Wrong study design |
| --- | --- |

### Savage 2002

| **Reason for exclusion** | Intervention |
| --- | --- |

### Schaillée 2017

| **Reason for exclusion** | Wrong population |
| --- | --- |

### Sheltzer 2019

| **Reason for exclusion** | Population |
| --- | --- |

### SIEBER 2012

| **Reason for exclusion** | Intervention |
| --- | --- |

### Simmons 2017

| **Reason for exclusion** | Wrong study design |
| --- | --- |

### Tam 2016

| **Reason for exclusion** | Wrong population |
| --- | --- |

### Thompson 2017

| **Reason for exclusion** | Population |
| --- | --- |

### van Niekerk 2012

| **Reason for exclusion** | Wrong population |
| --- | --- |

### Watson 2007

| **Reason for exclusion** | Intervention |
| --- | --- |

### Wright 2022

| **Reason for exclusion** | Study design |
| --- | --- |

###### Footnotes

## Characteristics of studies awaiting classification

###### Footnotes

## Characteristics of ongoing studies

### Zlotowitz PhD 2010

| **Study name** | *Grime not crime: the psychological impact of a community-based music project for marginalized young people* |
| --- | --- |
| **Starting date** | Unknown |
| **Contact information** | Doctoral dissertation, UCL; University College London |
| **Notes** | Requested for interlibrary loan. BL requested from UCL but not received. |

###### Footnotes
